# Selenium in Action: Exploring the Biological Wonders of Hydroselenite Salts

**DOI:** 10.3390/molecules30081714

**Published:** 2025-04-11

**Authors:** Cristina Morán-Serradilla, Daniel Plano, Yadira Pastor, Iñigo Navarro-Blasco, Asif Raza, Arun K. Sharma, Carmen Sanmartín

**Affiliations:** 1 Department of Pharmaceutical Sciences, Universidad de Navarra, 31008 Pamplona, Spain; cmoran.3@alumni.unav.es (C.M.-S.); sanmartin@unav.es (C.S.); 2 Department of Microbiology and Parasitology, University of Navarra, 31008 Pamplona, Spain; ypastor@unav.es; 3 Department of Chemistry, Universidad de Navarra, 31008 Pamplona, Spain; inavarro@unav.es; 4 Department of Molecular and Precision Medicine, Penn State Cancer Institute, Penn State College of Medicine, 500 University Drive, Hershey, PA 17033, USA; mraza@pennstatehealth.psu.edu (A.R.); asharma1@pennstathealth.psu.edu (A.K.S.)

**Keywords:** bacteria, cancer, selenium, salt

## Abstract

Despite the wealth of data related to the advantages of formulating a wide range of compounds as salts to ameliorate their biological properties, there is scant information regarding the therapeutic potential of selenium (Se) salts. In this work, we have formulated six antibiotics as hydroselenite salts in order to compare their in vitro antibacterial and anticancer effects and evaluate if this approach could enhance their water solubility. In this regard, in almost all the cases, their solubility was increased by one order of magnitude. All the compounds were screened against a panel of three Gram-positive and three Gram-negative bacteria. Likewise, their antiproliferative activity was evaluated in breast, prostate, glioblastoma, and pancreatic cancer cell lines. Normal human dermal fibroblasts (NHDF) were used to determine their selectivity indexes (SI). Additionally, these novel hydroselenite salts were submitted to the National Cancer Institute (NCI) to study their antitumoral potential. Compounds **SLT-2** and **SLT-6** showed potent cytotoxicity against the glioblastoma cancer cell line, and their ability to induce apoptosis and reactive oxygen species (ROS) was further assessed. To conclude, we have demonstrated that the formulation of several antibiotics as hydroselenite salts could be a feasible approach to obtain biologically active compounds with an enhanced effect.

## 1. Introduction

Cancer is the leading cause of death and a major public health problem worldwide [1]. It is a well-known fact that the current therapeutic options for this disease are still not satisfactory. Over the years, ample evidence has indicated that the efficacy of many drugs in the treatment of distinct tumors is often hindered by the intrinsic or developed drug resistance of these malignancies [2]. Therefore, novel therapeutic agents with enhanced clinical efficacy and reduced side effects must urgently be identified and developed.

Antimicrobial resistance (AMR) to commercially available medicines is rising to dangerously high levels and is considered a global health challenge [3]. Thus, the emergence and spread of multidrug-resistant (MDR) bacteria and biofilms highlight the urgent need for new therapeutic strategies. Currently, one of the possible strategies to overcome these challenges is the direct manipulation of the structure of known antibacterial drugs with the prime aim of enhancing their potency.

Selenium (Se) is known to be an essential micronutrient that plays a crucial role in maintaining human health. It has been reported that the levels of this trace element are related to innate and adaptive immune responses to numerous infections and play a pivotal role in preventing tumor incidence and blocking cancer metastasis [4,5,6]. Emerging evidence supports the notion that the addition of Se moieties into a vast number of drugs has yielded novel derivatives with pronounced activities in several pathologies. Even if these Se-bearing compounds showcase an interesting biological profile, their actual clinical exploitability could be hindered by several limitations, such as their low aqueous solubility, which can result in an inadequate therapeutically relevant dose in blood [7].

Although it has been widely reported that salt formation is a feasible approach to improve the physicochemical properties (i.e., hygroscopicity, aqueous solubility, and physical stability) and biological activity of several drugs, studies of the therapeutic potential of Se salts are scarce in the literature [8,9,10,11,12,13,14,15,16,17,18,19]. In 2013, several pyrido[2,3-*d*]pyrimidines were formulated as hydroselenite salts in order to enhance their low water solubility and bioavailability. Remarkably, some of them were more potent than two standard agents (methylseleninic acid and topotecan) against the tumoral prostate PC-3 cell line [9]. Likewise, various fused isoselenazolium salts were able to inhibit human breast adenocarcinoma MCF-7 and mouse carcinoma 4T1 proliferation at nanomolar to low micromolar concentrations by increasing the mitochondrial reactive oxygen species (ROS) production and inhibiting the pyruvate-dependent metabolism [16]. It should also be noted that a series of fused selenazolinium derivatives demonstrated great in vitro antimicrobial activities against several ESKAPE-pathogen strains [17]. In the past four years, the synthesis and antibacterial activity of several polyselenium salts have been reported. Of note, one of them exhibited good biofilm eradication activity with greater values than its corresponding polysulfonium salt analog. It has been hypothesized that this could be a result of the higher effective charge of the Se cation, which increases its ability to absorb negatively charged bacterial membranes. Additionally, this salt exhibited low cytotoxicity toward the mouse epithelioid fibroblast (L929) cell line and a high selectivity index (SI) [11,12,13,14].

In light of this, herein we report six new hydroselenite salts (**SLT1-6**) of different available antibiotics (ciprofloxacin, trimethoprim, dapsone, sulfadiazine, isoniazid, and gentamicin), aiming to compare their antibacterial and antitumoral properties and evaluate the possible advantages of these forming hydroselenite salts. For almost all the antibiotics, their formulation as hydroselenite salts improved their water solubility. Additionally, all the reported compounds were screened against a panel of six bacterial strains. To the best of our knowledge, this is the first time that the antibacterial activity of a series of hydroselenite salt derivatives has been reported. Likewise, their anticancer effect was assessed against four different cancer cell lines. The selectivity of these novel derivatives has also been determined. A single dose (10 μM) of the reported hydroselenite salts was used by the National Cancer Institute (NCI) 60 cell lines panel assay. From the data obtained in this study, it seems clear that formulating all the antibiotics, except ciprofloxacin, as hydroselenite salts yielded outstanding cytotoxic agents towards the central nervous system (CNS) cancer cell lines. **SLT-2** and **SLT-6** were selected to investigate their ability to induce apoptosis and ROS in U251 cells. Taken together, these hydroselenite salt candidates could provide promising new lead derivatives for further potential antibacterial and antitumoral drug development.

## 2. Results and Discussion

### 2.1. Chemistry and Characterization

**Figure 1:**
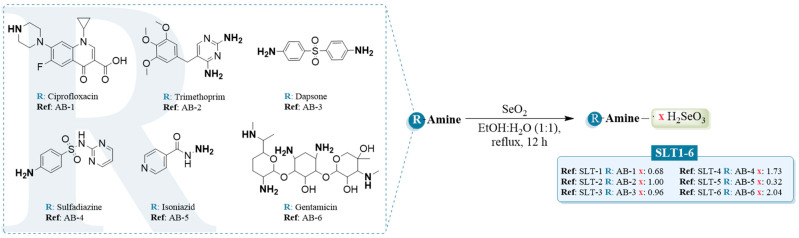
General procedure for the synthesis of the novel hydroselenite salts (SLT-1-6).

The hydroselenite salts (**SLT1-6**) were obtained in a one-pot synthesis by treating the corresponding antibiotics (ciprofloxacin, trimethoprim, dapsone, sulfadiazine, isoniazid, and gentamicin) with selenium dioxide (SeO_2_) in a mixture of ethanol:water (1:1) under heating as depicted in Figure 1.

Focusing on the chemical reaction process to obtain the hydroselenite salts, there are several positive aspects, such as the short reaction sequence, good yields of title salts (89–93%), as well as their ready isolation without further purification. Additionally, all the reported derivatives synthesized were stable at room temperature (r.t.) for at least 6 months. Their purity and structures were confirmed by ^1^H, ^13^C, and ^77^Se nuclear magnetic resonance (NMR) as reported in the Material and Methods section. The most relevant spectroscopic information for the hydroselenite salts was found in both ^13^C and ^77^Se spectra. Inspection of ^77^Se NMR spectra of all the hydroselenite salts revealed that a Se peak appeared as one sharp peak in the range of 1300–1320 ppm, confirming the presence of a Se atom in the structure of the new derivatives. The full spectra of all the hydroselenite salts are available in the Supplementary Materials (Figures S1–S18). To further corroborate the Se content and determine its accurate amount in the novel hydroselenite salts, we performed the atomic absorption spectroscopy (AAS) as described in the material and methods section.

### 2.2. Water Solubility Studies

The differences between the solubilities of the parent antibiotics and their corresponding hydroselenite salts were assessed by quantitative proton NMR (^1^H-qNMR). AB-6 and SLT-6 were not evaluated given that the parent antibiotic presents a very high water solubility, and we considered that the potential increase in this solubility with the salt formulation is not as interesting as in the other cases. The results are outlined in Table 1, and the spectra of all the compounds tested are available in the Supplementary Materials (Figures S19–S28).

From the above data, it seems clear that the formulation of all the studied antibiotics as hydroselenite salts enhanced their water solubility by one order of magnitude in almost all cases. Thus, this chemical modification might be a novel strategy to increase the solubility of several therapeutic agents.

### 2.3. Biological Evaluation

#### 2.3.1. Antibacterial Activity of the Reported Compounds

As a first approach to assess the antibacterial activity of the reported novel salts and the corresponding antibiotics, their minimum inhibitory concentration (MIC) and minimum bactericidal concentration (MBC) values were determined in a panel of six bacterial strains by the broth micro-dilution method (Table 2). Thus, they were assessed against the Gram-positive bacteria *Staphylococcus aureus* (*S. aureus*), *Staphylococcus epidermidis* (*S. epidermidis*), and *Bacillus sphaericus* (*B. sphaericus*) and the Gram-negative bacteria *Escherichia coli* (*E. coli*), *Klebsiella pneumoniae* (*K. pneumoniae*), and *Pseudomonas aeruginosa* (*P. aeruginosa*). As the antibacterial activity of H_2_SeO_3_ has not been previously reported, this compound was used in this study for comparison purposes. The compounds having MIC values higher than 200 µg/mL were considered inactive.

According to data depicted in Table 2, it can be observed that the Gram-positive bacteria strains are more sensitive to the hydroselenite salts. This difference might be attributed to the charge of the cell envelopes. As a result, Gram-negative bacteria would repel the hydroselenite salts more effectively than the cell envelopes of the Gram-positive ones, leading to a decreased inhibition of their bacterial growth [20]. An interesting point is that several salts (SLT-3-6) improved the activity of their corresponding parent antibiotic in at least one bacterial strain. Amidst them, *S. aureus* was more susceptible to the hydroselenite salts. Only compound SLT-1 was less potent than the corresponding antibiotic. Conversely, the formulation of dapsone, sulfadiazine, and gentamicin as hydroselenite salts (**SLT-3**, **SLT-4**, and **SLT-6**) furnished more active compounds. Notably, dapsone, sulfadiazine, and isoniazid were completely inactive against *S. epidermidis,* and no MIC and MBC values could be determined, whereas their corresponding hydroselenite salts (**SLT-3**, **SLT-4**, and **SLT-6**) displayed some activity with MIC values ranging from 50 to 200 μg/mL. Although *B. sphaericus* was the most resistant strain, it should be highlighted that **SLT-1** presented the same MIC and MBC values as ciprofloxacin. Even if **SLT-6** had less potent inhibitory effects than gentamicin, it presents a higher bactericidal potential with an MBC value of 0.39 μg/mL.

Concerning the results obtained for the Gram-negative bacteria, it can be observed that **SLT-1**, **SLT-2,** and **SLT-6** exhibited an antibacterial effect. Regarding the data obtained for *E. coli*, **SLT-2** and **SLT-6** maintained the activity of the unmodified drugs, while **SLT-1** enhanced the bactericidal effect of ciprofloxacin. Remarkably, this compound also resulted in an increased activity of the unmodified antibiotic towards *K. pneumoniae* with MIC and MBC values of 0.05 μg/mL. **SLT-2** and **SLT-6** were demonstrated to be as effective as their corresponding parent antibiotics (dapsone and gentamicin, respectively) against *P. aeruginosa*.

It should also be highlighted that H_2_SeO_3_ was almost devoid of activity in all the bacterial strains herein evaluated (Table 2). Thus, it can be concluded that the release of the Se salt of the molecules is not responsible for these activities. Taken together, the results displayed in Table 2 showed that the formulation of antibiotics as hydroselenite salts can be a feasible approach to obtain novel agents with an interesting effect against several bacterial strains. Based on these data, further studies would be required to assess the therapeutic potential of hydroselenite salts.

#### 2.3.2. Evaluation of the Antiproliferative Activities of the Antibiotics and Their Corresponding Salts

**Figure 2:**
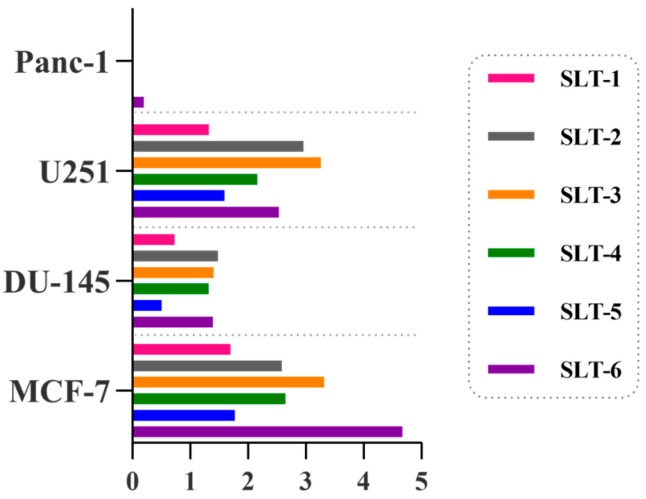
SI of all the hydroselenite salts. The SI was calculated as the ratio of the IC_50_ values determined for the non-malignant NHDF cells and the tumor cells.

To compare the antitumoral properties of the novel salts and their corresponding antibiotics, their antiproliferative activity was evaluated in vitro against four tumor cell lines derived from various human cancer types: MCF-7 (human breast adenocarcinoma cells), DU-145 (prostate cancer), U251 (glioblastoma cancer), and Panc-1 (pancreatic cancer). In vitro evaluation of their anticancer efficacy was determined by the 3-(4,5-dimethylthiazol-2-yl)-2,5-diphenyltetrazolium bromide (MTT) assay as previously described [21]. As the lack of selectivity is linked with undesired side effects, normal human dermal fibroblasts (NHDF) were used to determine the potential selectivity of all the reported molecules. These cell lines were treated for 48 h with each compound at seven concentrations ranging from 1 to 100 µM. The half-maximal inhibitory concentration (IC_50_) values are depicted in Table 3. The SI was determined as the ratio between the IC_50_ values obtained for the NHDF cells and the ones obtained for each cancer cell line (Figure 2).

An overview analysis of the IC_50_ values obtained and summarized in Table 3 showed that the selected cancer cell lines exhibited different sensitivity profiles, the breast and glioblastoma cells being the most sensitive ones to the Se salts. Among the tested compounds, SLT-6 stood out as it presented outstanding antitumoral activity in both cell lines, with an IC_50_ value of 0.58 and 1.07 µM on MCF-7 and U251 cells, respectively. This derivative also was fairly selective in the breast cancer cell line with an SI value of around 5. In addition, derivatives SLT-1-4 exhibited potent antiproliferative activity in these cell lines, with IC_50_ values below 5 µM. Another interesting point from the data depicted in Table 3 is the strong inhibitory activity of all the hydroselenite salts, except SLT-5, on the cell growth of DU-145, presenting IC_50_ values below 10 µM. Conversely, all the compounds herein reported were devoid of activity in Panc-1 cells except **SLT-6,** which displayed significant effectiveness against the pancreatic cancer cell line, exhibiting an IC_50_ value of 13.85 µM. Nevertheless, it should be highlighted that the potency of all the salts surpassed the one obtained for their corresponding parent antibiotics in the rest of the cell lines tested.

The striking difference in the potency of Se salts as compared to their corresponding parent compounds raises a question: Could the release of the Se salt part of the molecules be solely responsible for these activities? Nevertheless, after thoroughly analyzing the in vitro antiproliferative activities and the structure of the reported compounds, our initial hypothesis is that the antitumor activity of the new selenium salts might not be due to the release of the Se inorganic, as evinced by the following four experimental outcomes: (1) All the salt compounds should have shown similar efficacy at the same molar concentrations if the toxicity was solely due to the release of the Se salt part. For example, the 5- to 10-fold difference in the IC_50_ values of **SLT-5** and **SLT-6** against different cancer cell lines (Table 3) shows that the toxicity should not be due to the release of the Se moiety but the newly acquired properties of the intact individual compound; (2) the amount of Se at the IC_50_ values demonstrated by the Se salts is way lower than the reported values of sodium selenite [22,23,24]; (3) sodium selenite has been reported in several experimental conditions to possess IC_50_ values in the low micromolar range in Panc-1 cells, although 5 out the 6 Se salts did not present cytotoxic activity towards this cell line in our experiments even up to 100 µM [25,26]; (4) the NMR spectra (Supplementary Materials) have been registered in D_2_O and it can be observed that the hydroselenite is still attached to the drug scaffold and not released to the solvent, as it should be required for Se salt part to exert the anticancer activity. To confirm this hypothesis and due to the scarce number of reported antitumor activities of selenous acid, we evaluated H_2_SeO_3_ in the U251 cell line in our experimental setup. The results (Figure S60) showed that the selenous acid presents an IC_50_ value of 1.04 µM in this cell line. Since the IC_50_ value of selenous acid is in the range of what is observed for SLT-1-6 (Table 3), the increased antiproliferative activity of the salts might or might not be linked to the release of the Se salt fragment of the molecule. Although unlikely, if the former mechanism is followed, the extent of release possibly varies depending on the overall structure of the compound and the cell line used since each SLT compound exhibits a different IC_50_ value (Table 3). Further studies are needed to fully characterize and understand the behavior of these selenous salts.

Overall, the inclusion of Se in the structure of these antibiotics by formulating them as hydroselenite salts was a determinant for the antiproliferative activity, yielding novel molecules with an interesting and potent cytotoxic profile.

#### 2.3.3. The Hydroselenite Salts Inhibited the Cell Viability of U251 Cancer Cells

**Figure 3:**
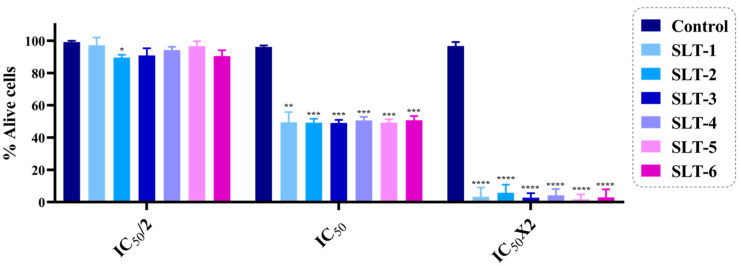
Effect of the reported hydroselenite salts on the inhibition of cell viability. Data are expressed as the mean ± SD of three independent experiments. **** *p* < 0.0001, *** *p* < 0.001, ** *p* < 0.01, and * *p* < 0.05 when comparing compounds with the control.

To corroborate the antiproliferative results obtained for all the novel hydroselenite salts in the MTT assay, we performed the trypan blue dye exclusion assay in U251 cells as previously described [27]. This method allows one to visually quantify the number of viable cells present in a cell suspension. Cells that present a blue cytoplasm are considered nonviable as they do not present an intact cell membrane that can exclude the dye. The cell viability was determined as the number of viable cells divided by the total number of cells counted on the hemacytometer. The results are expressed as the percentage of live cells (Figure 3). Cells were treated with three concentrations of each derivative [twice the IC_50_ (2 × IC_50_), IC_50_, and half the IC_50_ (IC_50_/2)], and the population of dead and alive cells was calculated after 48 h of treatment. From the data, it seems clear that the results were in concordance with the abovementioned ones obtained with the MTT assay.

#### 2.3.4. NCI-60 Analysis of the Hydroselenite Salts

**Figure 4:**
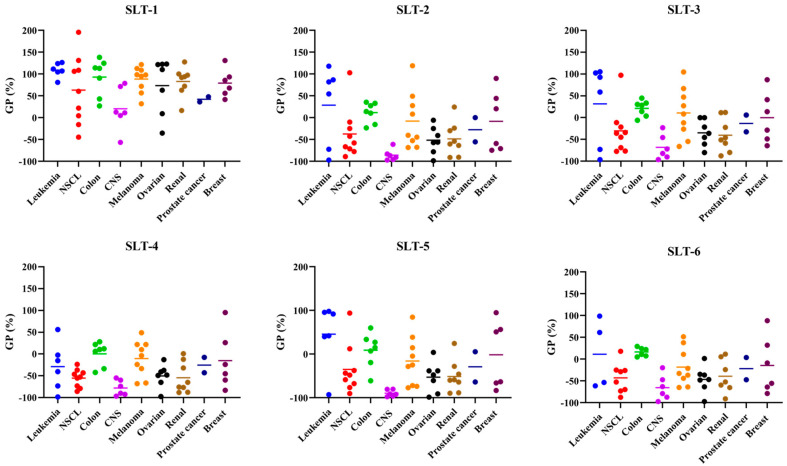
GP (%) of the NCI-60 human cancer cell lines after treatment with a single dose of 10 µM of **SLT-1** (NSC 854102), **SLT-2** (NSC 854103), **SLT-3** (NSC 854104), **SLT-4** (NSC 854105), **SLT-5** (NSC 854106), and **SLT-6** (NSC 854107).

**Figure 5:**
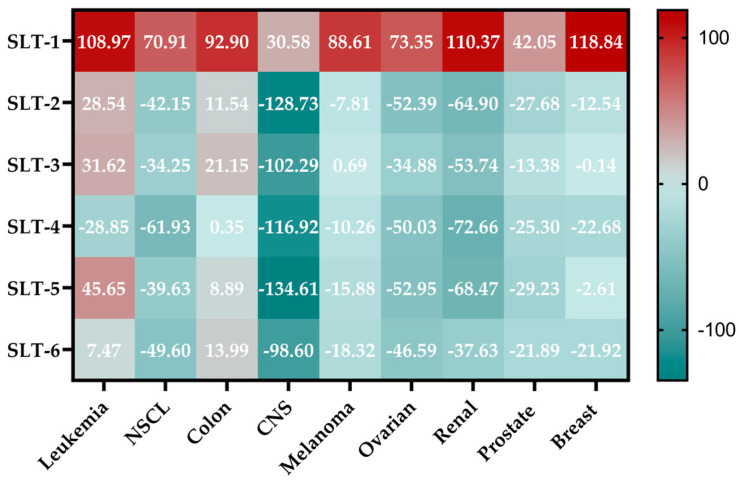
Summary of the average GP values (%) obtained for each cancer type in the NCI-60 cytotoxicity screening at 10 µM of all the novel reported hydroselenite salts.

In order to further study the antiproliferative activity of these novel hydroselenite salts, all the compounds were submitted to the Developmental Therapeutics Program (DTP) of NCI. They were screened at 10 μM for 48 h against a panel of 60 cancer cell lines, including leukemia, non-small cell lung cancer (NSCL), colon cancer, CNS cancer, melanoma, ovarian cancer, renal cancer, prostate cancer, and breast cancer cell lines. The results are reported as the growth percentage (GP) of the treated cells with each hydroselenite salt compared to the untreated controls. The data of the screening are summarized in Figure 4, and the mean GP values of the NCI-60 cancer cell line panel are depicted in Figure 5. The full reported data are gathered in the Supplementary Materials.

As shown in Figure 4, the different cell line panels presented different sensitivity profiles to the action of these derivatives. Surprisingly, the ciprofloxacin derivative (**SLT-1**) exhibited low cytotoxic activity, with an average cell growth of 75.44%. In sharp contrast, the other hydroselenite salts depicted a considerable and myriad spectrum of activity towards several cancer cell lines, with mean cell growth values ranging from −14.07 to −35.89%. From the above data (Figure 5), it seems clear that the CNS panel was the most sensitive and the leukemia and colon cell lines the most resistant ones. Nevertheless, close examination of the data revealed that the compounds **SLT-2-5** demonstrated potent cytotoxic activity against HCC-2998 cells, the most resistant cell line from the colon panel, with GP values ranging from −6.04 to −33.75%. Likewise, derivatives **SLT-2-6** revealed cytotoxic effects against other resistant cancer cell lines from the NCI cell panel, including SNB-19 (CNS cancer), SK-OV-3 and OVCAR-5 (ovarian cancer), TK-10 (renal cancer), NCI-H322M (NSCL), and SK-MEL-28 (melanoma cancer). It is also noteworthy that all the compounds, except **SLT-1**, presented outstanding cytotoxic effects towards the U251 cell line. Regarding the prostate panel, it can be observed that **SLT-4** exhibits a cytotoxic activity towards the DU-145, whereas compounds **SLT-2-6** displayed an antiproliferative effect against this cell line. Moreover, these hydroselenite salts (**SLT-2-6**) exhibited potent cytotoxic activity against the PC-3 cells. Additionally, they showed selectivity towards the breast cancer subpanel, as in half of the cell lines tested (MCF-7, HS T-47D, and MDA-MB-468), they were devoid of a cytotoxic effect.

#### 2.3.5. Five-Dose Assay

Compounds **SLT-2-6** were selected for further testing in a five-dose (0.01, 0.1, 1, 10, and 100 μM) screen since they fulfilled the set NCI one-dose screening parameters for threshold inhibition. The results are represented in three response parameters: (a) 50% growth inhibitory concentration (GI_50_), which is the concentration of a compound that leads to a 50% reduction in cell growth; (b) total growth inhibition (TGI), which represents the dose that completely inhibits cell growth; and (c) 50% lethal concentration (LC_50_), which indicates the concentration of a compound that causes a 50% death of initial cells at the end of the incubation period of 48 h. The outcomes of the five-dose analysis of all the hydroselenite salts are summarized in Table 4, Table 5 and Table 6, and the detailed results are presented in the Supplementary Materials. All the tested compounds exhibited potent anticancer activity against all the CNS cancer cell lines with mean GI_50_ values between 0.97 μM and 1.80 μM (Table 4). Amidst them, three of these hydroselenite salts (**SLT-3**, **SLT-4**, and **SLT-6**) prompted inhibition of cell growth with GI_50_ values in the low micromolar range in at least one cell line from this subpanel. As shown in Figures S43 and S55, **SLT-4** and **SLT-6** displayed remarkable inhibitory activity towards the most resistant CNS cell line, SNB-19, with a GI_50_ value of 0.80 μM and 0.64 μM, respectively. Likewise, all the tested hydroselenite salts exhibited GI_50_ values below 1 μM in at least one of the resistant cell lines from the NSCL subpanel (NCI-H322M, EKV, NCI-H226, A549, and HOP-62). Strikingly, **SLT-4** and **SLT-6** displayed outstanding inhibitory activity against NCI-H322M, the most resistant cell line, as they exhibited the same GI_50_ value (0.32 μM) and TGI values of 0.78 μM and 0.95 μM, respectively. Another important aspect perceived from Figure S55 is that SLT-6 presented a GI_50_ value of 0.09 μM towards the resistant breast cancer cell line HS 578T. According to the data summarized in Table 5, it can be observed that all the hydroselenite salts exhibited mean TGI values below 10 μM in both CNS and prostate subpanels. Regarding the LC_50_ values (Table 6), it should be pointed out that the colon and melanoma subpanels were the most resistant to all the evaluated compounds, whereas the CNS subpanel was the most susceptible. Inspection of the data related to the latter revealed that **SLT-2** and **SLT-4** displayed the overall most potent cytotoxic activities.

Glioblastoma multiforme is one of the most common and aggressive primary brain tumors. Regarding the poor prognosis and few recent therapeutic advances made for the treatment of this disease [28], we decided to further investigate the compounds that displayed a high selectivity and anticancer activity in U251 cells along with a great water solubility and results from the NCI assays. Thus, **SLT-2** and **SLT-6** were selected for further biological studies.

#### 2.3.6. SLT-2 and SLT-6 Induced Apoptosis in U251 Cancer Cells

**Figure 6:**
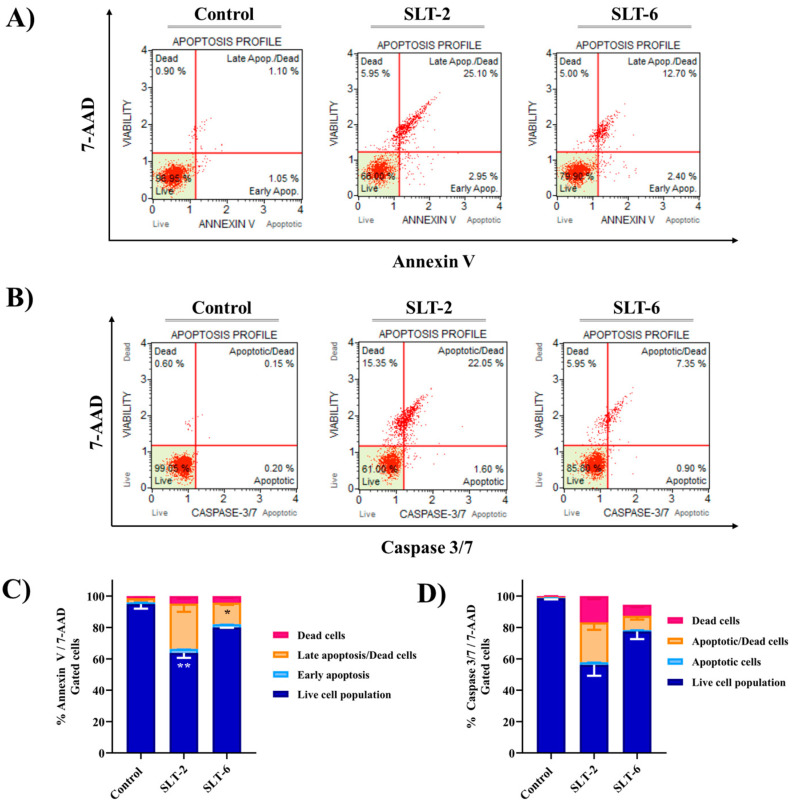
**SLT-2** and **SLT-6** induced apoptotic cell death in U251 cells. (**A**) Cells were treated with 1 μM of each derivative and examined on a Muse™ automated cell analyzer (Tokyo, Japan) with the Annexin V and Dead Cell apoptosis assay. (**B**) An analogous independent experiment was performed with the caspase 3/7 apoptosis assay. (**C**,**D**) Quantification of the cell population obtained in both experiments. Data are presented as the mean ± SD of two independent experiments. ** *p* < 0.01, * *p* < 0.05 when comparing control and compounds.

The cell viability assays abovementioned evinced that **SLT-2** and **SLT-6** exhibited antiproliferative activity. To further confirm if these hydroselenite salts could induce cell death through apoptosis, we performed the Annexin V and Dead Cell and caspase 3/7 apoptosis assays. Currently, Annexin V is routinely used to detect apoptosis, as it can specifically bind to the surface-exposed phosphatidylserine (PS) residues during the early stages of the apoptotic cell death process. Furthermore, 7-aminoactinomycin D (7-AAD) is used to stain and exclude late apoptotic and dead cells in flow cytometry. On the other hand, it is widely recognized that the members of the caspase family play several pivotal roles in the regulation of apoptosis. Amidst them, executioner caspase-3 and caspase-7 are involved in the degradation of different regulatory and structural proteins, therefore contributing to the morphological hallmarks of this cell death process [29]. Hence, U251 cells were treated either with compound **SLT-2** or **SLT-6** for 48 h at 1 µM. The results are depicted in Figure 6. Moreover, a quantitative comparison of the difference in the cell population induced by each compound in both assays is represented in Figure 6C,D.

Cells treated with the vehicle [dimethyl sulfoxide (DMSO)] mainly occupied the bottom left quadrant (96.95%), indicating a healthy state, and were used as controls in both assays. According to the results obtained in the Annexin V and Dead Cell assay, it can be observed that both compounds induced a shift from healthy cells towards an apoptotic state (Figure 6A,C). Thus, the population of viable cells was decreased while the percentage of late apoptotic or dead cells was consequently increased with respect to the control. This effect was more pronounced for compound **SLT-2**. Notably, for this compound, 25.20% of the cells were in the late apoptotic/dead stage, whilst 2.95% were in the early stage of apoptotic death. Additionally, for both hydroselenite salts, it can be observed that only a slight percentage of cells were necrotic (5–5.95%). Concerning the caspase 3/7 assay, both compounds exhibited similar behavior as observed in the other assay. An increase in the population of apoptotic/dead cells (~22%) compared to the control could be observed for **SLT-2** (Figure 6B,D). This effect was more moderate in the case of **SLT-6**. Nevertheless, it is worth mentioning that only a slight percentage of cells were necrotic for the latter, as there were almost no cells located in the upper left quadrant.

As a concluding point, our data suggest that both hydroselenite salts, **SLT-2** and **SLT-6,** induce apoptosis in the U251 cancer cell line at 1 µM, with a clear involvement of caspase 3/7 in the case of **SLT-2**.

#### 2.3.7. Influence of SLT-2 and SLT-6 on ROS Levels

**Figure 7:**
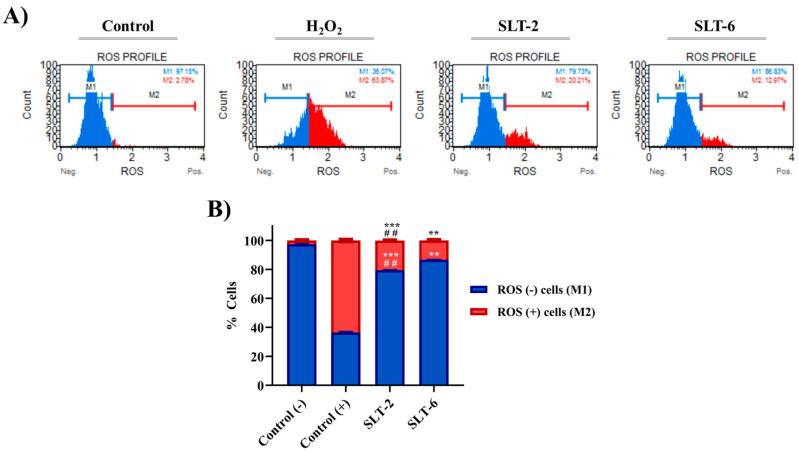
Effect of **SLT-2** and **SLT-6** on ROS levels. (**A**) U251 cells were treated with both hydroselenite salts for 24 h and subjected to Muse flow cytometry-based oxidative stress analysis for total ROS levels measurement. (**B**) Quantification of the cell population with the oxidative stress assay. Data are presented as the mean ± SD of two independent experiments. *** *p* < 0.001 and ** *p* < 0.01 when comparing control and compounds; ## *p* < 0.01 when comparing compounds.

Burgeoning evidence has shown that the antitumoral activities of a wide range of Se-containing derivatives are associated with an increase in total ROS levels [30]. To investigate whether the cytotoxic effects of the hit hydroselenite salts are related to ROS production, we assessed total ROS levels in U251 cells treated with either DMSO (negative control), H_2_O_2_ (0.9 μM, positive control), **SLT-2** (1 µM), or **SLT-6** (1 µM) for 24 h, and the total ROS level was measured. As shown in Figure 7, the 24 h treatment with the examined compounds led to a significant increase in the total ROS levels compared to the negative control. Notably, the treatment with **SLT-6** led to a small but significant change in the percentage of ROS, (+) whereas **SLT-2** displayed a more pronounced effect. Nevertheless, in order to confirm that the increase in the ROS levels is related to the induction of apoptosis by these hydroselenite salts, further studies need to be carried out.

## 3. Materials and Methods

### 3.1. Chemistry

Starting materials, reagents, and solvents were purchased from commercial suppliers and used as received without further purification. The reactions were monitored by thin-layer chromatography (TLC), and the spots were visualized under ultraviolet (UV) light. Elemental analyses for C, hydrogen (H), and nitrogen (N) were obtained with a Thermo Fisher FlashSmart™ Elemental Analyzer (Waltham, MA, USA). The NMR (^1^H, ^13^C, and ^77^Se) spectra were recorded on a Bruker Avance Neo 400 MHz (Billerica, MA, USA) operating at 400, 100, and 76 MHz, respectively, using deuterium oxide (D_2_O) or deuterated DMSO (DMSO-*d*_6_) as solvents and tetramethylsilane (TMS) as the internal standard. Chemical shift (δ) values are reported in parts per million (ppm), and coupling constants (*J*) values are in hertz (Hz). Melting points (mp) were determined with a Mettler FP82 + FP80 apparatus (Greifensee, Switzerland). All the newly synthesized hydroselenite salts were >95% pure.

#### 3.1.1. General Procedure for the Synthesis of the Hydroselenite Salts (SLT-1-6)

The preparation of the hydroselenite salts was carried out in a one-pot synthesis as previously reported in the literature. Briefly, the antibiotics (10 mmol) were dissolved in ethanol:water (1:1, 50 mL), and SeO_2_ (15 mmol) was added carefully. The resulting mixture was refluxed and stirred for 12 h. Afterwards, the solvent was removed under reduced pressure. Of note, this reaction allowed the isolation of the final hydroselenite salts with no further purification methods.

*1-Cyclopropyl-6-fluoro-4-oxo-7-(piperazin-1-yl)-1,4-dihydroquinoline-3-carboxylic acid · 0.68 hydroselenite* (**SLT-1**). The compound was obtained from **AB-1** as a white solid according to the general procedure described above. Yield: 95%, m.p: 189.7 °C. ^1^H NMR (400 MHz, D_2_O) δ 8.58 (s, 1H, NH), 7.47 (s, 1H, Aryl), 7.45 (s, 1H, Aryl), 3.65–3.62 (m, 1H, Aliph), 3.57–3.55 (m, 4H, Aliph), 3.46–3.43 (m, 4H, Aliph), 1.35 (m, 2H, Aliph), 1.13 (m, 2H, Aliph). ^13^C NMR (101 MHz, D_2_O) δ 176.1 (C=O), 169.2 (C=O), 152.2 (d, J_*C-F*_ = 249.3 Hz), 148.4, 144.6 (d, J_*C-F*_ = 10.2 Hz), 139.0, 119.0, 110.8 (d, J_*C-F*_ = 23.5 Hz), 106.7, 106.1, 46.3 (CH_2_, piperazine), 43.2 (CH_2_, piperazine), 36.1 (CH, cyclopropyl), 7.4 (CH_2_, cyclopropyl). ^77^Se NMR (76 MHz, D_2_O) δ (ppm): 1318. Anal. Calcd for C_17_H_18_FN_3_O_3_ ·0.68 H_2_SeO_3_ (%): C, 48.72; H, 4.62; N, 10.03. Found: C, 48.65; H, 4.88; N, 9.89.

*5-(3,4,5-Trimethoxybenzyl)pyrimidine-2,4-diamine · 1.0 hydroselenite* (**SLT-2**). The compound was obtained from **AB-2** as a pale orange solid according to the general procedure described above. Yield: 93%, m.p: 61.6°C. ^1^H NMR (400 MHz, D_2_O) δ 7.18 (s, 1H, Aryl), 6.42 (s, 2H, Aryl), 3.65 (s, 6H, Aliph), 3.59 (s, 3H, Aliph), 3.50 (s, 2H, Aliph). ^13^C NMR (101 MHz, D_2_O) δ 164.4, 154.3, 152.6, 139.6, 135.5, 133.1, 109.0, 106.0, 60.8 (-OCH_3_), 55.9 (-OCH_3_), 32.4 (-CH_2_-). ^77^Se NMR (76 MHz, D_2_O) δ (ppm): 1309. Anal. Calcd for C_14_H_18_N_4_O_3_ · 1.0 H_2_SeO_3_ (%): C, C, 46.78; H, 4.77; N, 13.36. Found: C, 46.92; H, 4.62; N, 13.50.

*4,4′-Sulfonyldianiline · 0.96 hydroselenite* (**SLT-3**). The compound was obtained from **AB-3** as a purple solid according to the general procedure described above. Yield: 95%, m.p: 106.7 °C. ^1^H NMR (400 MHz, DMSO) δ 7.44 (d, *J* = 8.8 Hz, 4H, Aryl), 6.58 (d, *J* = 8.9 Hz, 4H, Aryl). ^13^C NMR (101 MHz, DMSO) δ 153.2 (-C-NH_2_), 129.0, 128.6 (-C-SO_2_), 113.3. ^77^Se NMR (76 MHz, DMSO) δ (ppm): 1314. Anal. Calcd for C_12_H_12_N_2_O_2_S · 0.96 H_2_SeO_3_ (%): C, 38.73; H, 3.74; N, 7.53. Found: C, 38.49; H, 3.96; N, 7.39.

*4-Amino-N-(pyrimidin-2-yl)benzenesulfonamide · 1.73 hydroselenite* (**SLT-4**). The compound was obtained from **AB-4** as a dark pink gel according to the general procedure described above. Yield: 84%. ^1^H NMR (400 MHz, D_2_O) δ 8.36 (d, *J* = 5.3 Hz, 2H, Aryl), 7.94 (d, *J* = 8.8 Hz, 2H, Aryl), 7.37 (d, *J* = 8.8 Hz, 2H, Aryl), 6.94 (t, *J* = 5.3 Hz, 1H, Aryl). ^13^C NMR (101 MHz, D_2_O) δ 159.2, 157.6, 155.6, 139.3, 128.8, 127.9, 123.3, 122.8, 112.6. ^77^Se NMR (76 MHz, D_2_O) δ (ppm): 1301. Anal. Calcd for C_10_H_10_N_4_O_2_S · 1.73 H_2_SeO_3_ (%): C, 25.36; H, 2.84; N, 11.83. Found: C, 25.01; H, 2.65; N, 12.01.

*Isonicotinohydrazide · 0.32 hydroselenite* (**SLT-5**). The compound was obtained from **AB-5** as a white solid according to the general procedure described above. Yield: 96%, m.p: 195.7 °C. ^1^H NMR (400 MHz, DMSO) δ 8.80 + 8.78 (d, *J* = 5.2 Hz, 2H, Aryl, signal split by hydrogen bond), 7.82 (d, *J* = 4.6 Hz, 2H, Aryl). ^13^C NMR (101 MHz, DMSO) δ 166.7 + 164.8 (C=O, signal split by hydrogen bond), 151.0, 139.8+ 138.6 (signal split by hydrogen bond), 123.2 + 121.8 (signal split by hydrogen bond). ^77^Se NMR (76 MHz, DMSO) δ 1315. Anal. Calcd for C_6_H_7_N_3_O · 0.32 H_2_SeO_3_ (%): C, 40.39; H, 4.28; N, 23.55. Found: C, 40.19; H, 4.17; N, 23.33.

*2-((4,6-Diamino-3-((3-amino-6-(1-(methylamino)ethyl)tetrahydro-2H-pyran-2-yl)oxy)-2-hydroxycyclohexyl)oxy)-5-methyl-4-(methylamino)tetrahydro-2H-pyran-3,5-diol · 2.04 hydroselenite* (**SLT-6**). The compound was obtained from **AB-6** as an orange gel according to the general procedure described above. Yield: 94%. ^1^H NMR (400 MHz, D_2_O) δ 5.78 (ddd, *J* = 17.8, 9.6, 3.6 Hz, 1H), 5.01 (d, *J* = 3.7 Hz, 1H), 4.10 (dd, *J* = 10.9, 3.7 Hz, 1H), 4.06–3.93 (m, 1H), 3.89 (d, *J* = 12.8 Hz, 1H), 3.77–3.69 (m, 2H), 3.55–3.43 (m, 3H), 3.41 (d, *J* = 12.8 Hz, 1H), 3.37 (d, *J* = 10.9 Hz, 1H), 3.28–2.91 (m, 1H), 2.79 (s, 3H), 2.62 (s, 1H), 2.43 (dd, *J* = 13.2, 4.0 Hz, 1H), 2.03–1.70 (m, 4H), 1.47 (td, *J* = 13.4, 5.3 Hz, 1H), 1.22 (s, 3H), 1.17 (dt, *J* = 6.9, 3.1 Hz, 2H). ^13^C NMR (101 MHz, D_2_O) δ 101.1, 94.7, 83.6, 76.2, 76.1, 75.7, 74.5, 74.4, 70.1, 69.9, 69.1, 68.8, 67.8, 66.2, 66.0, 63.2, 57.4, 51.0, 49.7, 49.4, 48.5, 42.6, 34.5, 31.1, 27.6, 25.4, 22.9, 20.9, 20.5, 14.1, 12.3, 9.5. ^77^Se NMR (76 MHz, DMSO) δ 1302. Anal. Calcd for C_21_H_43_N_5_O_7_ · 2.04 H_2_SeO_3_ (%): C, 34.05; H, 6.36; N, 9.46. Found: C, 34.28; H, 6.71; N, 9.17.

#### 3.1.2. Atomic Absorption Spectroscopy (AAS)

Se contents in the synthesized hydroselenite salts were determined using flame AAS by means of a Perkin Elmer AAnalyst 800 atomic absorption spectrometer (Perkin Elmer, Hamburg, Germany) equipped with a deuterium lamp for background correction and a flame atomizer using a mixture of air and acetylene at flow rates of 17.0 and 2.0 L/min, respectively. A Se electrodeless discharge lamp was used as the radiation source, operating at 196.0 nm with a current of 290.0 mA and a slit width of 0.2 nm. Instrumental parameters were configured in accordance with the instrument’s instructions, whereas burner head adjustment, lamp alignment, and nebulizer flow were optimized to achieve maximum signal intensity.

Matrix-matched calibration solutions were prepared by spiking with an appropriate volume of a working standard Se solution of 1000 mg/L (Merck, Darmstadt, Germany) to give nominal Se concentrations of 5.0, 15.0, and 45.0 mg/L in nitric acid solution (0.2% *w*/*v*). To quantify Se, a solution of each sample of the synthesized hydroselenite salts was prepared by accurately weighing 50 mg each in 10 mL of a 0.2% nitric acid solution; subsequently, a forty-fold dilution was carried out. In addition, an internal quality control solution was prepared, yielding a Se concentration of 16.0 mg/L. This control sample was previously measured to assess accuracy (*n* = 6, 16.01 ± 0.14 mg/L). Additionally, a recovery study involving spiking at three different concentrations (5.0, 10.0, and 15.0 mg/L) within the linear calibration range yielded satisfactory results, with recovery rates between 96 and 102%, ensuring accurate Se determination in synthesized hydroselenite salt samples.

#### 3.1.3. Water Solubility Assay by ^1^H-NMR

The solubility of the antibiotics and the novel hydroselenite salts was evaluated via ^1^H-qNMR spectroscopy using dimethyl sulfone (DMSO2) internally as a reference material. This method is based on the consideration that the integration for a given signal that is selected as a marker for a given compound is proportional to its concentration [31]. Therefore, solutions of 0.5 mL in D_2_O with an excess of each compound were prepared, along with 2–3 mg of DMSO2. ^1^H-NMR spectra were recorded at 298 K on a Bruker Avance Neo at a proton resonance frequency of 400 MHz. The solubilities were calculated considering the ratio between the area of the peaks belonging to the internal standard and the different hydrogens of the antibiotics and their corresponding hydroselenite salts.

### 3.2. Biology

#### 3.2.1. MIC and MBC Assays

All the compounds were screened for their in vitro antibacterial activity against a panel of three Gram-positive (*S. aureus*, *S. epidermidis*, and *B. sphaericus*) and three Gram-negative (*E. coli*, *K. pneumoniae*, and *P. aeruginosa*) bacteria using the broth micro-dilution method. Bacterial strains were kindly provided by the Department of Microbiology and Parasitology of the University of Navarra. The bacteria were grown in tryptic soy agar (TSA) plates and maintained at 4 °C. A stock solution of the compounds was prepared in DMSO (1 mg/mL), and they were serially diluted in round-bottom 96-well plates in tryptic soy broth (TSB) with eleven concentrations ranging from 0.2 to 200 µg/mL with a final volume of 100 µL. Then, the same volume of a bacterial dilution containing 10^5^ CFU/mL was added to each well. Untreated bacteria were used as negative controls. The plates were incubated at 37 °C for 24 h. After the incubation period, the presence or absence of visual growth of bacterial cells was analyzed for each well. The lowest concentrations of the corresponding salt and antibiotic at which no visible growth occurred were considered the MIC values. Furthermore, 10 µL of the bacterial suspension from the MIC, MIC/2, and MIC/4 wells were added to TSA plates and incubated overnight at 37 °C. The concentrations that did not show any bacterial growth were considered as the MBC values. Results were reported as the mean of three test runs.

#### 3.2.2. Cell Culture Conditions

American Type Culture Collection (ATCC, Manassas, VA, USA) and PromoCell (Heidelberg, Germany) provided all the cell lines. The cancer cell lines MCF-7 and DU-145 were maintained in RPMI-1640 medium (Gibco, Waltham, MA, USA), supplemented with 1% of antibiotics (10.00 units/mL penicillin and 10.00 mg/mL streptomycin; Gibco), and 10% of heat-inactivated FBS (Gibco). Panc-1 and U251 cells were maintained in Dulbecco’s Modified Eagle Media (DMEM) (ThermoFisher Scientific, Waltham, MA, USA), supplemented with 10% FBS (Gibco) and 1% antibiotics (10.00 units/mL penicillin and 10.00 mg/mL streptomycin; Gibco). NHDF juvenile foreskin was cultured in PromoCell growth medium supplemented with fibroblast growth factor (FGF) at 10 ng/mL and 1% penicillin/streptomycin (Gibco). The cells were incubated in tissue culture flasks at 37 °C and 5% CO_2_.

#### 3.2.3. Cell Viability Assay

The antitumoral effect of the synthesized compounds and the antibiotics was tested at seven different concentrations (1, 2.5, 5, 10, 25, 50, and 100 µM) in MCF7, DU-145, U251, and Panc-1 cancer cell lines and NHDF non-malignant cells by MTT assay as previously described [27]. Compounds were dissolved in DMSO at a concentration of 10^−2^ M, and serial dilutions were prepared. A total of 1 × 10^4^ cells per well were seeded in flat-bottom 96-well microtiter plates in 100 µL of growth medium. After 24 h of incubation at 5% CO_2_ and 37 °C, the cells were treated with either DMSO or the respective increasing concentrations of each compound in fresh media for 48 h. Afterwards, the media were removed, and fresh media containing 20 μL of the MTT reagent (Promega, Madison, WI, USA) were added per well. Absorbances were measured at 550 nm in a 96-well multiscanner (Labsystems, Bradenton, FL, USA). The IC_50_ values were determined using OriginPro 8.5.1. software by nonlinear curve fitting. SIs were calculated as the ratio of the IC_50_ values determined for the non-malignant (NHDF) and the corresponding tumoral cell lines. GraphPad Prism 7.0 software was used to obtain the corresponding graphs. Data were obtained from at least three independent experiments performed in triplicate.

#### 3.2.4. Trypan Blue Staining Assay

U251 cells were seeded in 12-well plates at a density of 3 × 10^5^ cells per well for 24 h. After the incubation periods, cells were treated with three serial concentrations [twice the IC_50_ (2 × IC_50_), IC_50_, and half the IC_50_ (IC_50_/2)] of the hydroselenite salts for 48 h. The cells were harvested after the end of the treatments and mixed with a 1:1 volume of 0.4% trypan blue dye (Invitrogen, Waltham, MA, USA). Afterwards, the cells were loaded over the hemocytometer and counted separately using a bright field microscope. The viable cells presented a clear, bright appearance. Conversely, membrane-compromised or dead cells were stained with the dye. GraphPad Prism 7.0 software was used to obtain the corresponding graphs. Data were obtained from three independent experiments performed in triplicate.

#### 3.2.5. NCI-60 Analysis

All the salts were submitted to the DTP of NCI. Cytotoxicity activity was evaluated against a panel of 59 human tumor cell lines. The protocols are available on https://dtp.cancer.gov/discovery_development/nci-60/methodology.htm (accessed on 17 June 2024). Graphpad Prism 7.0 software was used to represent the corresponding graphs.

#### 3.2.6. Apoptosis Assays

Induction of apoptosis by **SLT-2** and **SLT-6** was assayed using the Annexin V and Dead Cell assay kit and the caspase 3/7 assay kit (EMD Millipore, Darmstadt, Germany). U251 cells were seeded in 6-well plates at a density of 4 × 10^5^ cells per well and maintained for 24 h at 37 °C in a humidified atmosphere of 5% CO_2_. Then, the cells were treated either with DMSO (control) or 1 µM of both hydroselenite salts and incubated for 48 h. At the end of treatment, cells were harvested and stained with the respective dyes according to the manufacturer’s protocols. In the case of the Annexin V assay, cells were stained with 100 μL of Muse™ Annexin V and Dead Cell Reagent and incubated for 20 min at r.t. protected from light prior to analysis. On the other hand, for the caspase 3/7 assay, cells were stained with 5 μL of Muse™ caspase 3/7 working solution and incubated for 30 min at 37 °C. After incubation, 150 mL of Muse™ caspase 7-AAD working solution was added and the samples were incubated for another 5 min at r.t. protected from light. Samples were further analyzed on a Muse™ Cell Analyzer (Merck Millipore, Darmstadt, Germany). Both assays led to four different populations of cells: viable cells (Annexin V, caspase 3/7, and 7-AAD_negative_ (lower left quadrant)); early apoptotic cells (both Annexin V and caspase 3/7_positive_ and 7-AAD_negative_ (lower right quadrant)); late apoptotic or dead cells (Annexin V, caspase 3/7, and 7-AAD_positive_ (upper right quadrant)); and necrotic cells (both Annexin V and caspase 3/7_negative_ and 7-AAD_positive_ (upper left quadrant)). Data were obtained from at least two independent experiments. GraphPad Prism 7.0 software was used to obtain the corresponding graphs.

#### 3.2.7. ROS Measurement

ROS levels were measured in U251 cells treated with 1 µM of **SLT-2** and **SLT-6** using the Muse Oxidative Stress Kit (EMD Millipore, Darmstadt, Germany) according to the manufacturer’s protocol. The U251 cells were seeded in 6-well plates at a density of 4 × 10^5^ cells per well and incubated for 24 h at 37 °C in a humidified atmosphere of 5% CO_2_. Afterwards, the cells were treated with 1 µM of the respective hydroselenite salts in the serum-enriched DMEM medium for 24 h. Then, the cells were harvested, and 10 μL of cells in suspension were added into each tube. Furthermore, 190 μL of Muse Oxidative Stress Reagent working solution was added to each tube. The samples were mixed thoroughly by vortexing at medium speed and incubated for 30 min at 37 °C. Samples were further analyzed on a Muse™ Cell Analyzer (Merck Millipore, Darmstadt, Germany). This kit is able to identify two cell populations: ROS (−) cells (blue M1 peak in the graph) and ROS (+) cells (red M2 peak in the graph). Cells treated with H_2_O_2_ (0.9 µM) were used as positive controls. The percentages of both cell populations were measured using the Muse™ Cell Analyzer (Merck Millipore, Darmstadt, Germany). Data were obtained from at least two independent experiments. GraphPad Prism 7.0 software was used to represent the corresponding graphs.

#### 3.2.8. Statistical Analysis

Data were expressed as the mean ± standard deviation (SD), and experiments were performed at least thrice in triplicate unless otherwise noted. Non-linear curve regression analysis calculated by OriginPro version 8.5.1. software was used to obtain the IC_50_ values for the hydroselenite salts and their corresponding antibiotics. The unpaired *t*-test was used to calculate the statistical significance of differences comparing the hydroselenite salts and control or the treatment with two different hydroselenite salts. Data were analyzed using GraphPad Prism version 8.0.1., and the statistically significant values (*p*-value) for unpaired *t*-test analysis were taken as **** *p* < 0.0001, *** *p* < 0. 001, ** *p* < 0.01, and * *p* < 0.05 when comparing control and compounds, and ## *p* < 0.01 when comparing hydroselenite salts.

## 4. Conclusions

In summary, we have synthesized a series of 6 novel hydroselenite salts and evaluated their antibacterial and antitumoral capabilities. To our knowledge, this constitutes the first report of the appealing in vitro antibacterial profile of hydroselenite salts. Our results demonstrated that the formulation of the antibiotic as hydroselenite salts enhanced their water solubility. Furthermore, the antibacterial activity of most of the hydroselenite salts was either superior to or comparable with their corresponding unmodified drugs. Additionally, the work presented evidence that this design, based on the incorporation of hydroselenite into several antibiotics, led to analogs with potent anticancer activity. Of note, all the hydroselenite salts were submitted to the DTP of the NCI, and most of them exhibited a promising antitumoral profile. Taking into account our in-house results and the data from the NCI screen, compounds **SLT-2** and **SLT-6** were identified as the most potent antiproliferative agents among all the Se-derivatives and antibiotics with IC_50_ values below 2 µM and high SI in the human glioblastoma cell line U251. Although we assessed whether the cytotoxic effects induced by these compounds are linked to apoptosis and ROS production, further research is necessary to fully comprehend the mechanism of action of these hydroselenite salts. Overall, considering the antibacterial activity and potency of these Se-derivatives on cancer cell viability, it appears that the formulation of some antibiotics as hydroselenite salts could serve as a promising launch point for further design of novel therapeutic agents for combating these pathologies.

## Figures and Tables

**Table 1:** Water solubility of the antibiotics and their corresponding hydroselenite salts in D_2_O.

Ref.	Water Solubility (g/mL)
AB-1	<1.62 × 10^−4^
SLT-1	5.91 × 10^−3^
AB-2	2.84 × 10^−4^
SLT-2	4.92 × 10^−3^
AB-3	1.52 × 10^−4^
SLT-3	3.34 × 10^−4^
AB-4	1.12 × 10^−4^
SLT-4	3.08 × 10^−3^
AB-5	5.35 × 10^−3^
SLT-5	5.93 × 10^−2^

**Table 2:** Antibacterial activity of the reported molecules against six bacterial strains represented as the MIC and MBC values (in μg/mL).

Ref.	Gram-Positive Bacteria						Gram-Negative Bacteria					
	*S. aureus*		*S. epidermidis*		*B. sphaericus*		*E. coli*		*K. pneumoniae*		*P. aeruginosa*	
	MIC	MBC	MIC	MBC	MIC	MBC	MIC	MBC	MIC	MBC	MIC	MBC
AB-1	0.39	0.78	0.2	0.2	6.25	6.25	0.03	3.13	0.1	0.2	0.1	0.39
SLT-1	0.78	0.78	0.78	1.56	6.25	6.25	0.2	0.78	0.05	0.05	0.2	1.56
AB-2	1.56	3.13	>200	>200	1.56	6.25	1.56	6.25	3.13	12.5	50	>200
SLT-2	1.56	3.13	>200	>200	3.13	12.50	1.56	6.25	6.25	12.5	50	>200
AB-3	>200	>200	>200	>200	>200	>200	>200	>200	>200	>200	>200	>200
SLT-3	100	200	50	50	>200	>200	>200	>200	>200	>200	>200	>200
AB-4	>200	>200	>200	>200	>200	>200	>200	>200	>200	>200	50	>200
SLT-4	100	>200	200	>200	>200	>200	>200	>200	>200	>200	>200	>200
AB-5	>200	>200	>200	>200	>200	>200	>200	>200	>200	>200	>200	>200
SLT-5	>200	>200	100	100	>200	>200	>200	>200	>200	>200	>200	>200
AB-6	25	50	0.39	0.39	0.2	0.78	25	25	1.56	3.13	6.56	12.5
SLT-6	12.5	12.5	0.39	1.56	0.39	0.39	25	25	3.13	12.5	6.56	12.5
H_2_SeO_3_	100	200	100	200	100	200	200	>200	100	200	200	>200

**Table 3:** IC_50_ (in µM) values for the parent antibiotics and their corresponding salts in MCF-7, DU-145, U251, Panc-1, and NHDF cell lines.

	MCF-7	DU-145	U251	Panc-1	NHDF
AB-1	23.38 ± 7.61	>100	>100	>100	>100
SLT-1	3.40 ± 0.47	7.95 ± 1.18	4.37 ± 1.21	>100	5.77 ± 1.50
AB-2	>100	>100	>100	>100	6.47 ± 1.79
SLT-2	2.23 ± 0.52	3.91 ± 0.77	1.95 ± 0.42	>100	5.41 ± 0.76
AB-3	>100	>100	>100	>100	>100
SLT-3	1.74 ± 0.35	4.11 ± 0.61	1.77 ± 0.19	>100	14.69 ± 6.27
AB-4	>100	>100	>100	>100	>100
SLT-4	1.11 ± 0.14	2.23 ± 0.52	1.36 ± 0.27	>100	2.94 ± 0.88
AB-5	>100	99.92 ± 0.20	>100	>100	41.23 ± 12.12
SLT-5	5.18 ± 0.37	18.33 ± 3.03	5.77 ± 0.75	>100	9.57 ± 2.36
AB-6	>100	>100	>100	>100	>100
SLT-6	0.58 ± 0.14	1.95 ± 0.28	1.07 ± 0.12	13.85 ± 4.62	2.71 ± 0.29

IC_50_ values are presented as the mean ± SD of at least three independent experiments determined by the MTT assay.

**Table 4:** Average values of GI_50_ (µM) of all the reported hydroselenite salts against the NCI subpanels in five-dose analysis.

Subpanel	Compound (Mean GI_50_)				
	SLT-2	SLT-3	SLT-4	SLT-5	SLT-6
Leukemia	25.37	25.91	2.38	16.39	21.76
NSCL	10.90	9.54	1.24	11.38	4.05
Colon	17.42	12.59	4.54	15.60	6.93
CNS	1.55	1.42	0.97	1.80	1.09
Melanoma	15.04	15.30	15.75	17.33	18.67
Ovarian	6.36	5.63	4.35	6.77	4.67
Renal	3.68	3.75	2.61	3.88	2.92
Prostate	2.34	2.40	2.17	2.49	2.51
Breast	33.96	24.01	10.42	45.28	13.36

**Table 5:** Average values of TGI (µM) of all the reported hydroselenite salts against the NCI subpanels in five-dose analysis.

Subpanel	Compound (Mean TGI)				
	SLT-2	SLT-3	SLT-4	SLT-5	SLT-6
Leukemia	16.90	29.30	20.39	36.23	25.50
NSCL	24.78	22.91	3.02	24.81	13.18
Colon	73.03	74.06	51.11	61.82	63.09
CNS	3.06	3.25	2.20	3.65	2.78
Melanoma	53.77	59.11	59.17	42.68	64.96
Ovarian	33.10	36.70	15.85	16.94	22.58
Renal	43.11	43.15	42.23	26.83	42.92
Prostate	4.67	4.86	3.73	5.57	4.61
Breast	61.69	61.56	48.84	62.07	53.47

**Table 6:** Average values of LC_50_ (µM) of all the reported hydroselenite salts against the NCI subpanels in five-dose analysis.

Subpanel	Compound (Mean LC_50_)				
	SLT-2	SLT-3	SLT-4	SLT-5	SLT-6
Leukemia	46.08	41.19	25.54	50.24	32.27
NSCL	64.29	68.94	59.10	46.25	64.44
Colon	100.00	100.00	89.16	78.47	100.00
CNS	5.69	24.71	4.43	7.48	28.78
Melanoma	96.30	84.11	87.90	62.92	86.62
Ovarian	68.26	68.50	53.48	44.75	69.95
Renal	68.91	81.26	62.48	60.96	68.98
Prostate	9.32	-	-	16.60	-
Breast	74.01	63.70	62.46	67.53	67.40

## Data Availability

Dataset available on request from the authors.

## Supplementary Materials

The following supporting information can be downloaded at https://www.mdpi.com/article/10.3390/molecules30081714/s1, Figures S1–S28: ^1^H-, ^13^C-, ^77^Se- and q-NMR spectra. Figures S29–S59: NCI-60 results for SLT-1-6. Figure S60: Antiproliferative activity of selenous acid towards U251 cells.
