# Supplementary material for: Selenium in Action: Exploring the Biological Wonders of Hydroselenite Salts
Source: Molecules. 2025 Apr 11;30(8):1714. doi: 10.3390/molecules30081714 (PMC12029531; doi:10.3390/molecules30081714)
Supplement: Supplementary file 1 [file molecules-30-01714-s001.zip › molecules-3318122-supplementary.pdf]

Selenium in Action: Exploring the Biological Wonders of Hydroselenite Salts

Cristina Morán-Serradilla [1], Daniel Plano [1,], Yadira Pastor [2], Iñigo Navarro-Blasco [3], Asif Raza [4], Arun K. Sharma [4], and Carmen Sanmartín [1]*

[1] Department of Pharmaceutical Sciences, Universidad de Navarra, 31008 Pamplona, Spain; cmoran.3@alumni.unav,es (C.M.-S.); dplano@unav.es (D.P.) sanmartin@unav.es (C.S.)

[2] Department of Microbiology and Parasitology, University of Navarra, Irunlarrea 1, 31008 Pamplona, Spain; ypastor@unav.es (Y.P.)

[3]Department of Chemistry, Universidad de Navarra, 31008 Pamplona, Spain; inavarro@unav.es (I.N.-B.)

[4] Department of Molecular and Precision Medicine, Penn State Cancer Institute, CH72, Penn State College of Medicine,500 University Drive, Hershey, PA 17033, USA; mraza@pennstatehealth.psu.edu (A.R.); asharma1@pennstathealth.psu.edu (A.K.S.)

Table of contents:

**Chemical characterization**	
**^{1}H, ^{13}C and ^{77}Se NMR spectra**	
**SLT-1**	**Figures S1-3**
**SLT-2**	**Figures S4-6**
**SLT-3**	**Figures S7-9**
**SLT-4**	**Figures S10-12**
**SLT-5**	**Figures S13-15**
**SLT-6**	**Figures S16-18**
**Quantitative ^{1}H NMR spectra**	
**AB-1**	**Figure S19**
**SLT-1**	**Figure S20**
**AB-2**	**Figure S21**
**SLT-2**	**Figure S22**
**AB-3**	**Figure S23**
**SLT-3**	**Figure S24**
**AB-4**	**Figure S25**
**SLT-4**	**Figure S26**
**AB-5**	**Figure S27**
**SLT-5**	**Figure S28**
**Biological evaluation**	
**NCI-60 results**	
**SLT-1**	**Figure S29**
**SLT-2**	**Figure S30-35**
**SLT-3**	**Figure S36-41**
**SLT-4**	**Figure S42-47**
**SLT-5**	**Figure S48-53**

SLT-6 **Figure S54-59**
Antiproliferative activity towards U251 cells
Selenous acid **Figure S60**

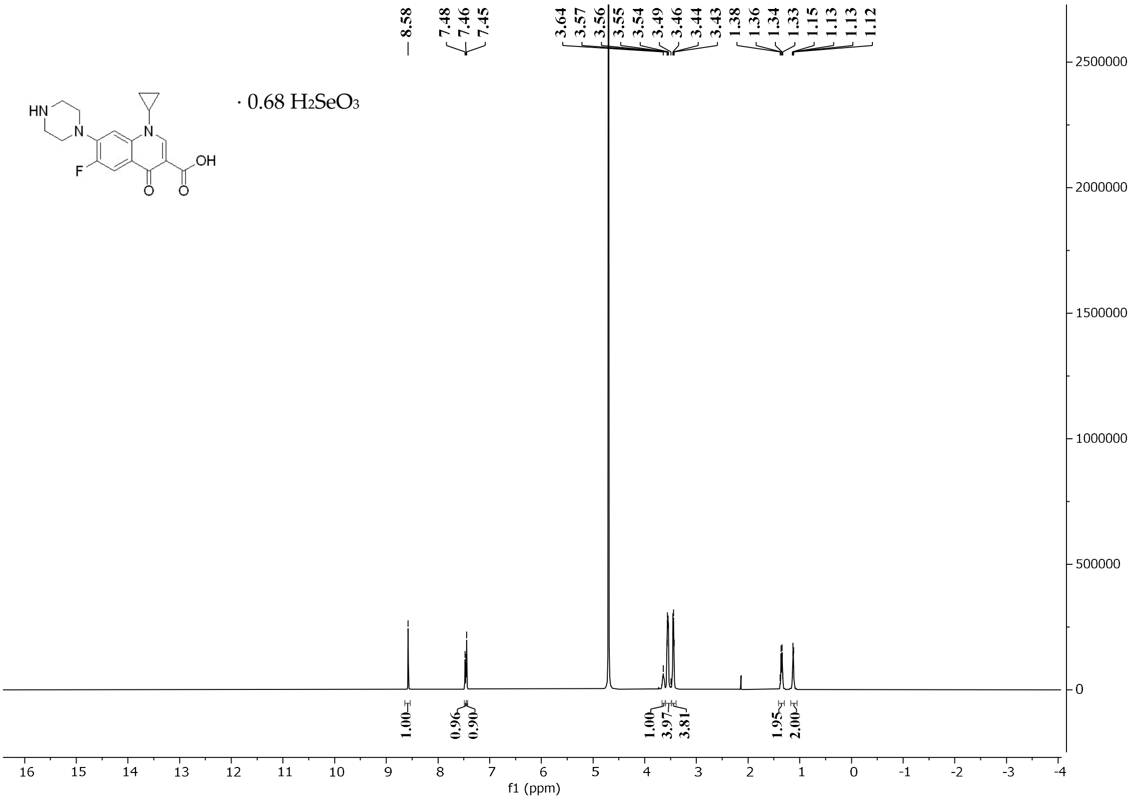

Figure S1. ^{1}H-NMR spectrum of compound **SLT-1.**

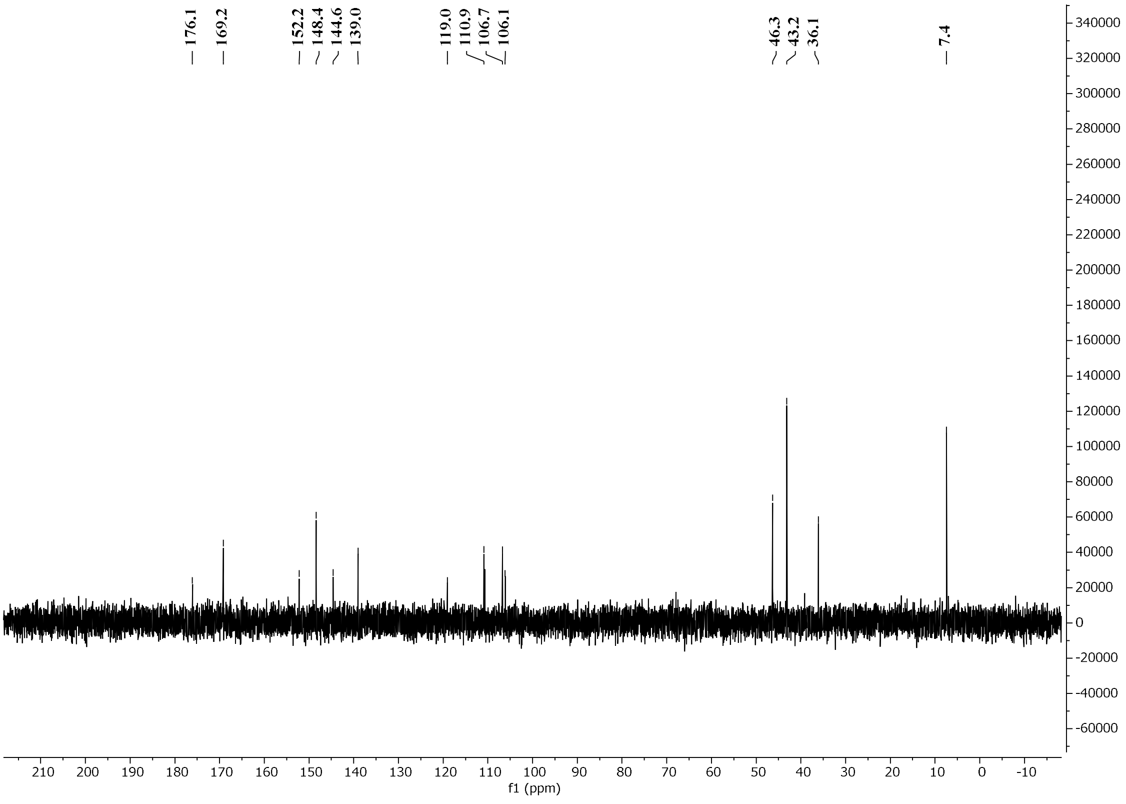

Figure S2. ^{13}C-NMR spectrum of compound **SLT-1**.

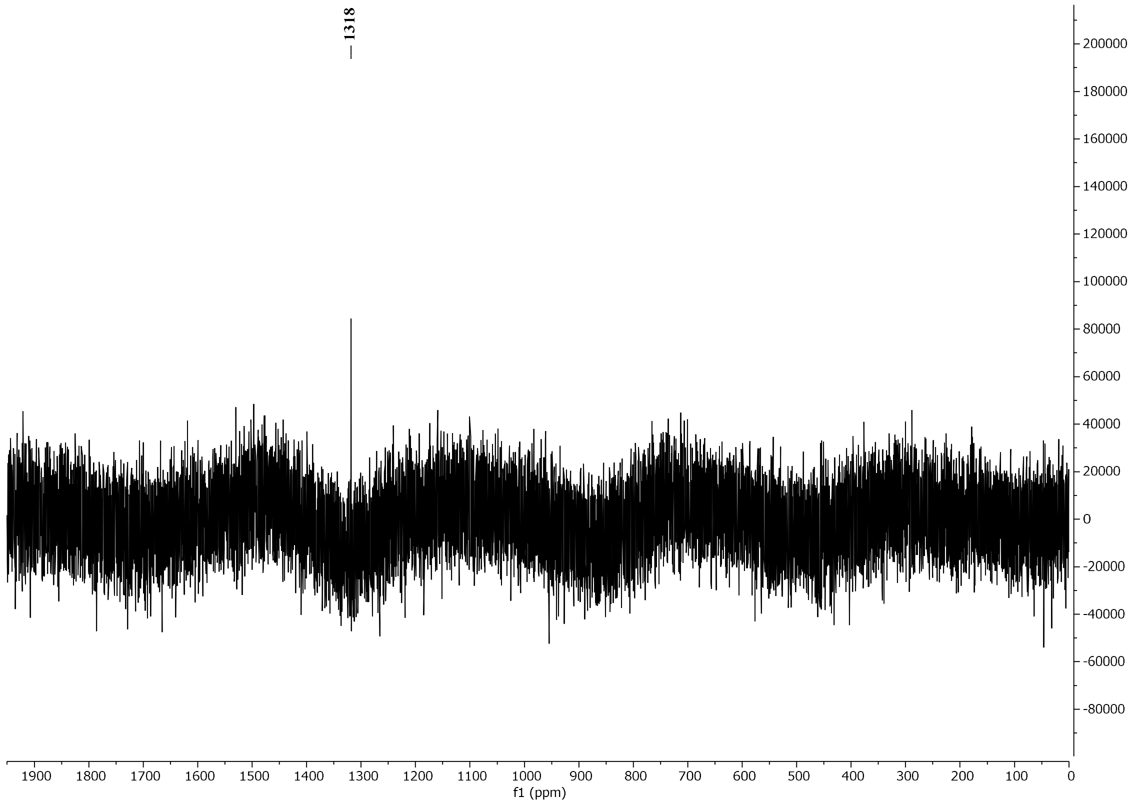

Figure S3. ^{77}Se-NMR spectrum of compound **SLT-1**.

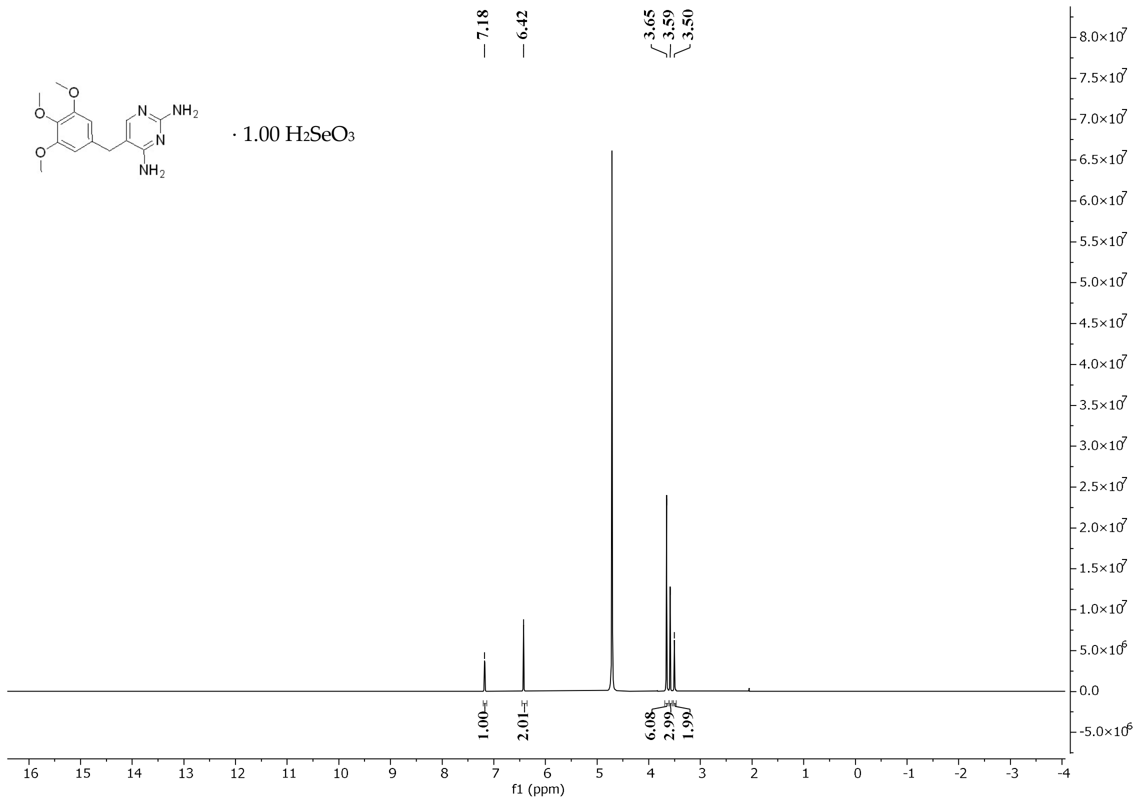

Figure S4. ^{1}H-NMR spectrum of compound **SLT-2**.

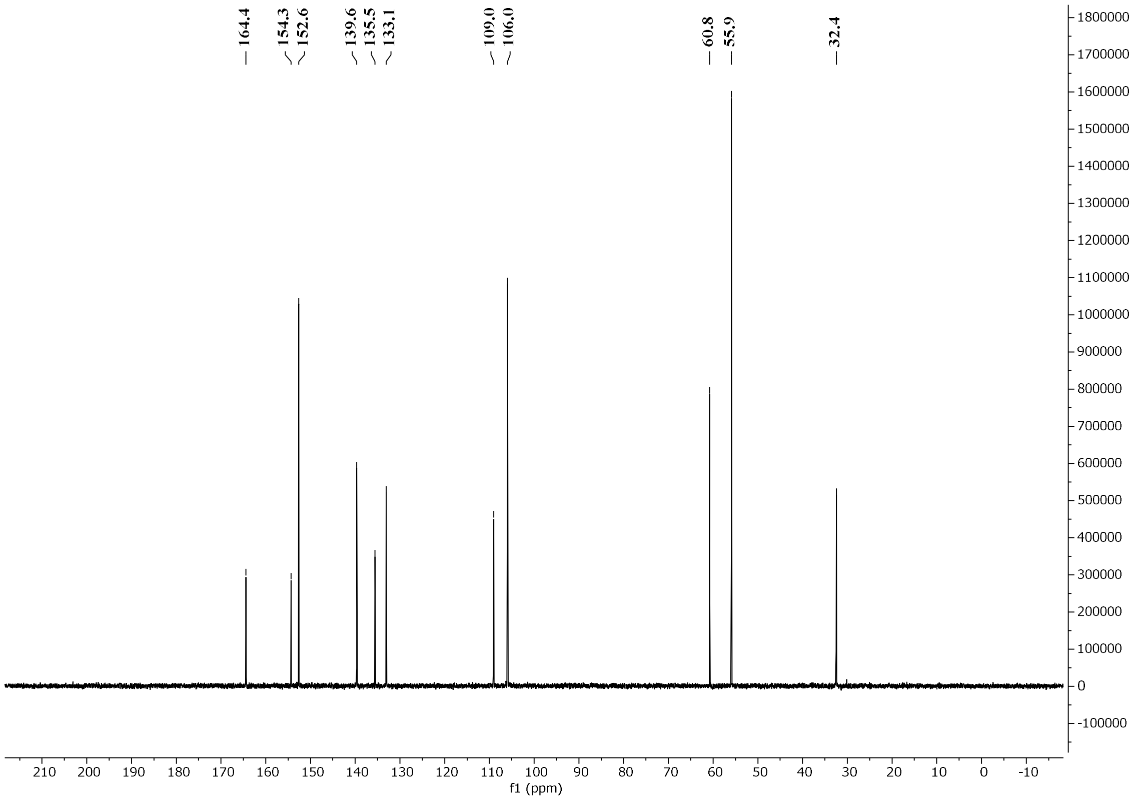

Figure S5. ^{13}C-NMR spectrum of compound **SLT-2**.

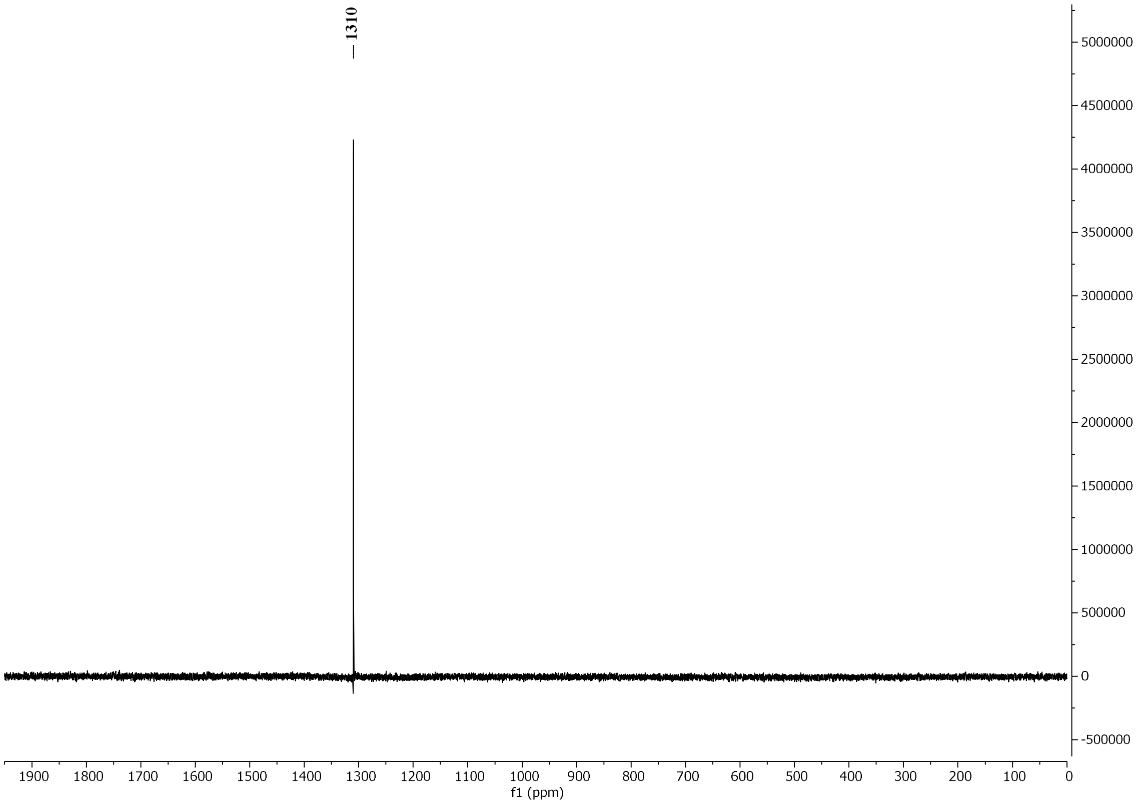

Figure S6. ^{77}Se-NMR spectrum of compound **SLT-2**.

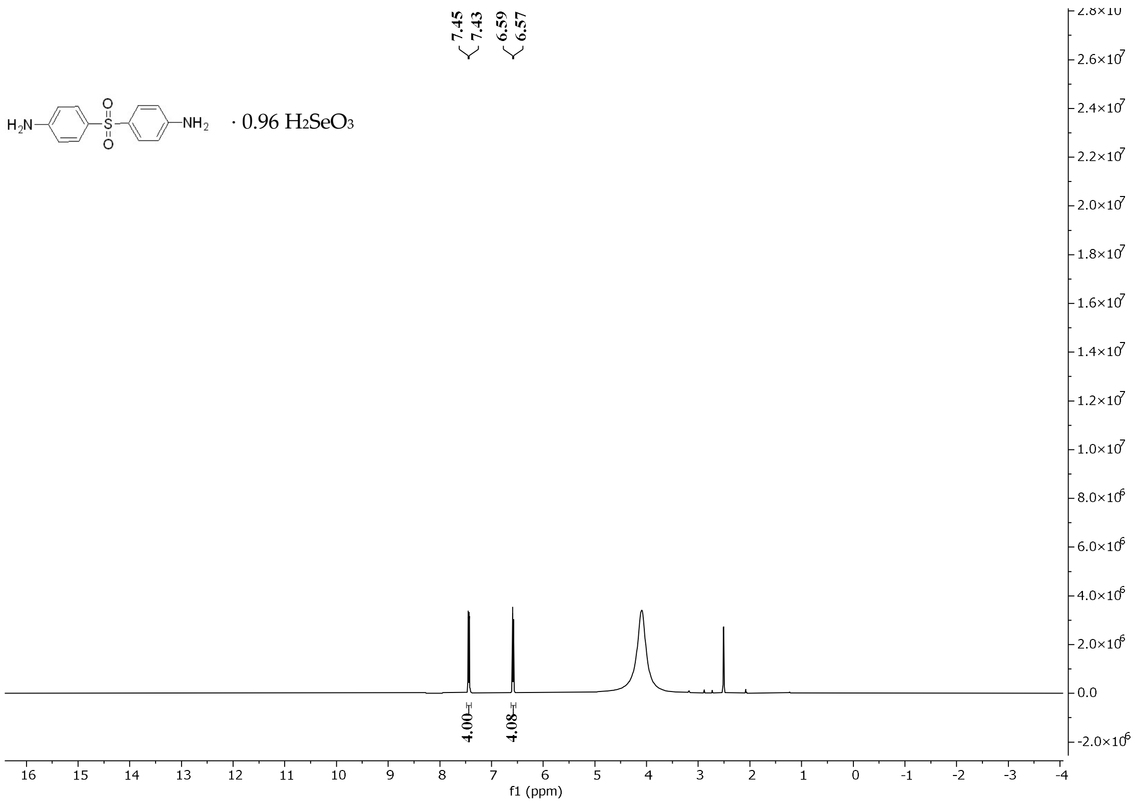

Figure S7. ^{1}H-NMR spectrum of compound **SLT-3**.

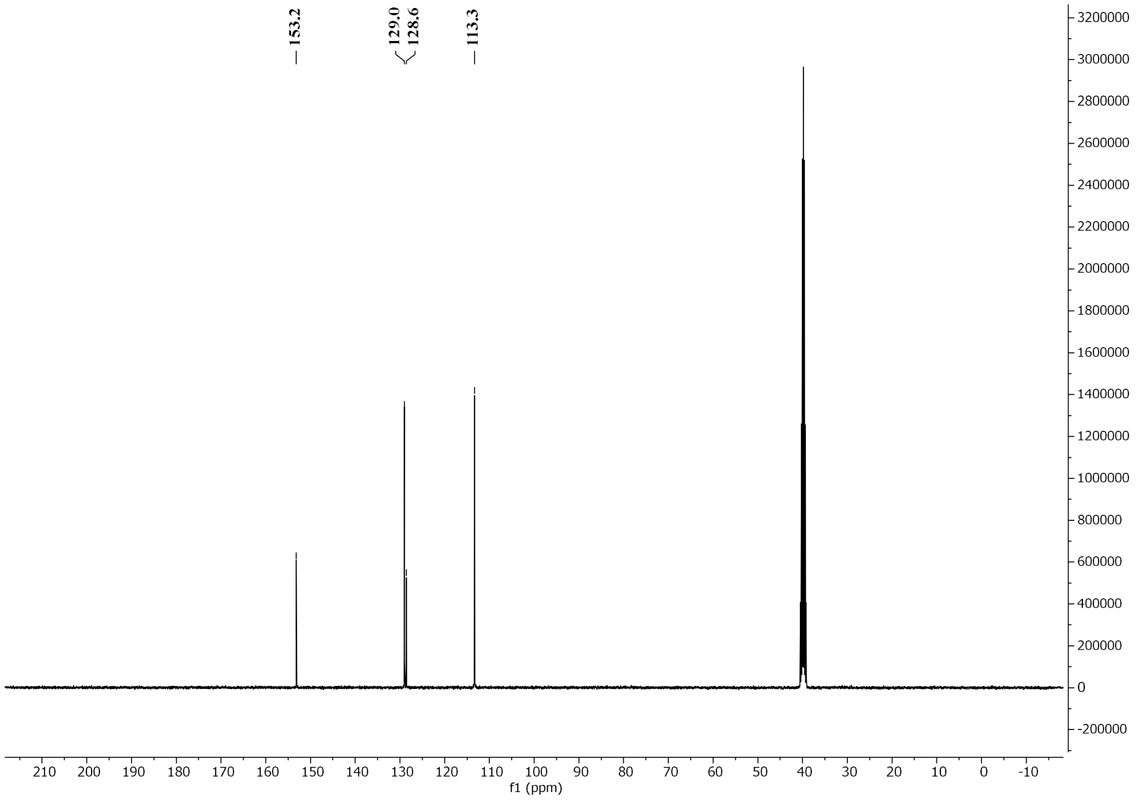

Figure S8. ^{13}C-NMR spectrum of compound **SLT-3**.

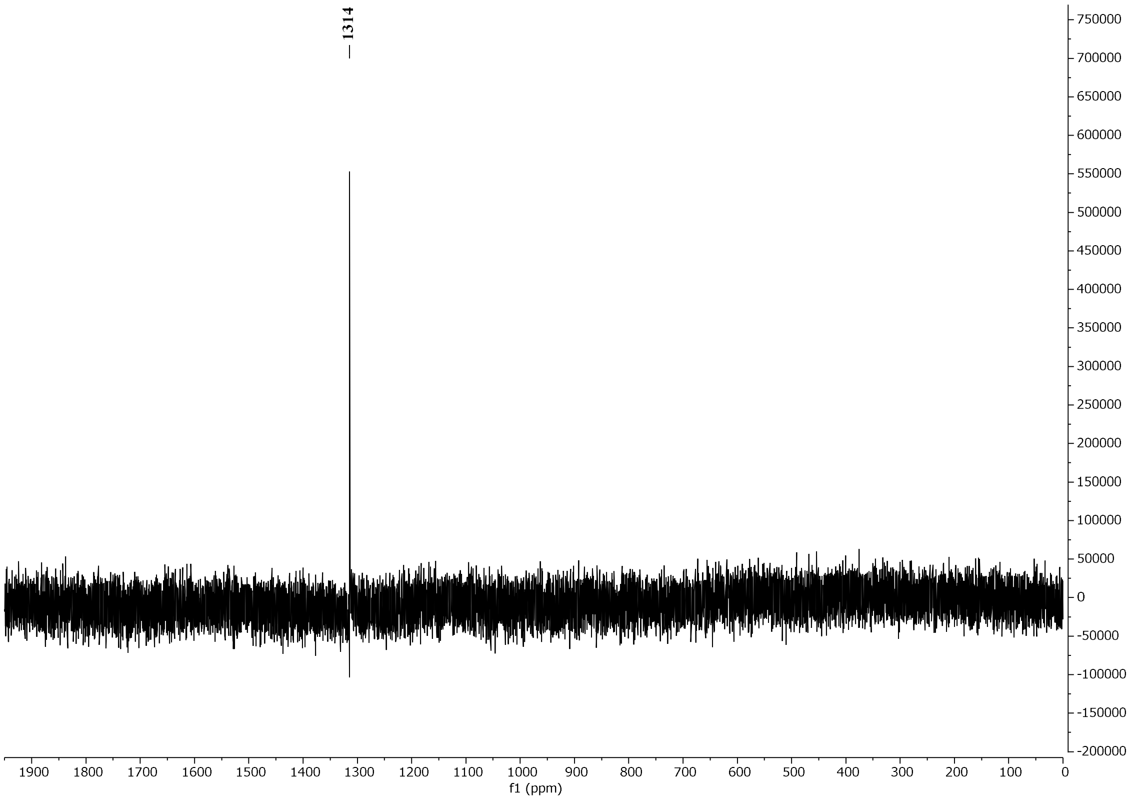

Figure S9. ^{77}Se-NMR spectrum of compound **SLT-3**.

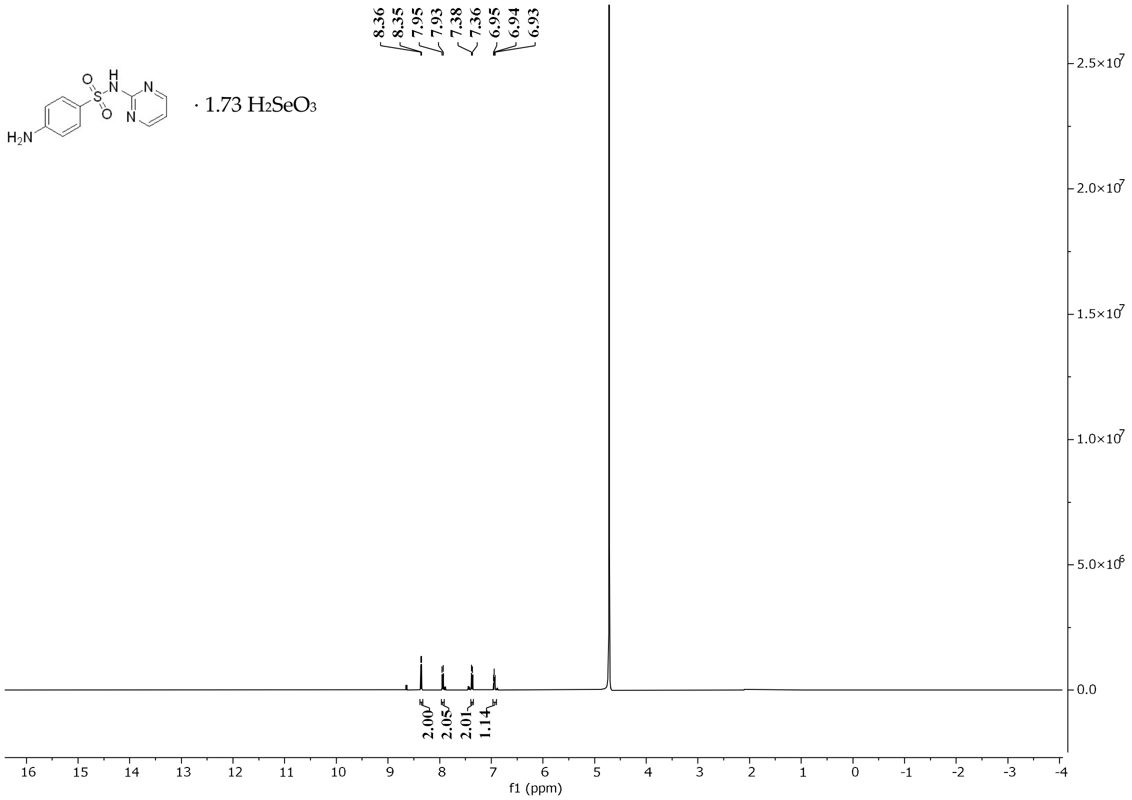

Figure S10. ^{1}H-NMR spectrum of compound **SLT-4**.

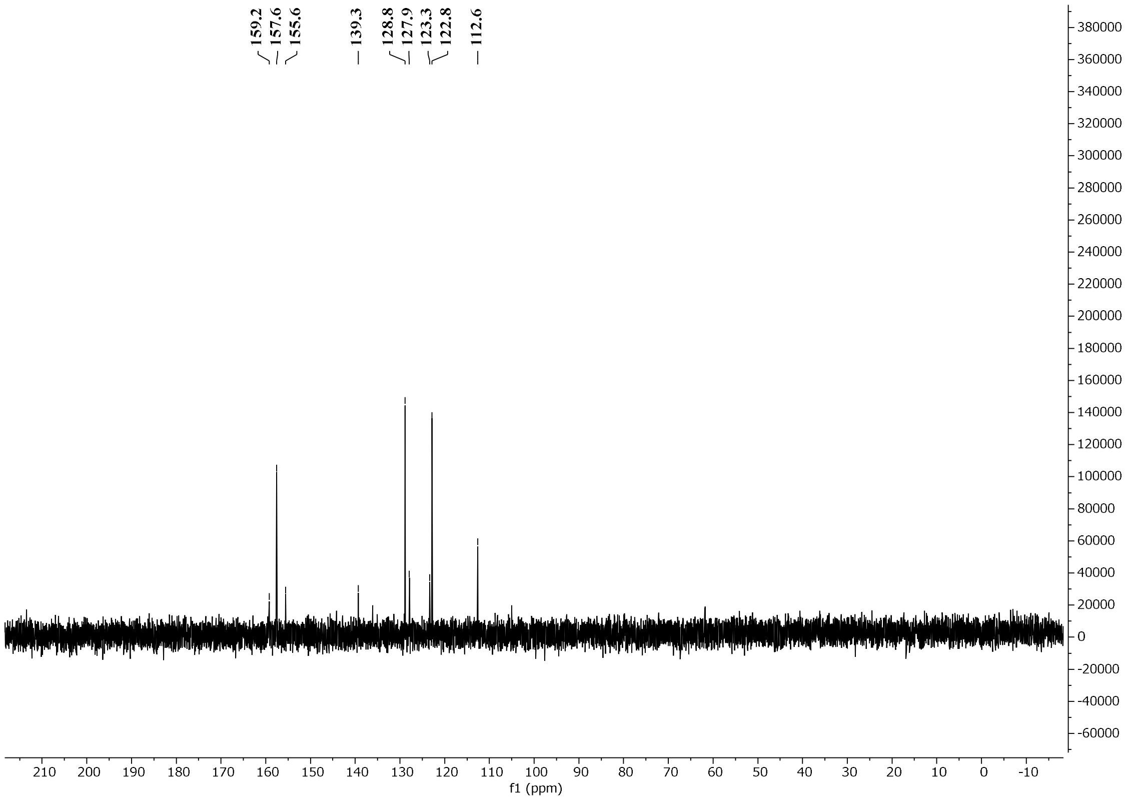

Figure S11. ^{13}C-NMR spectrum of compound **SLT-4**.

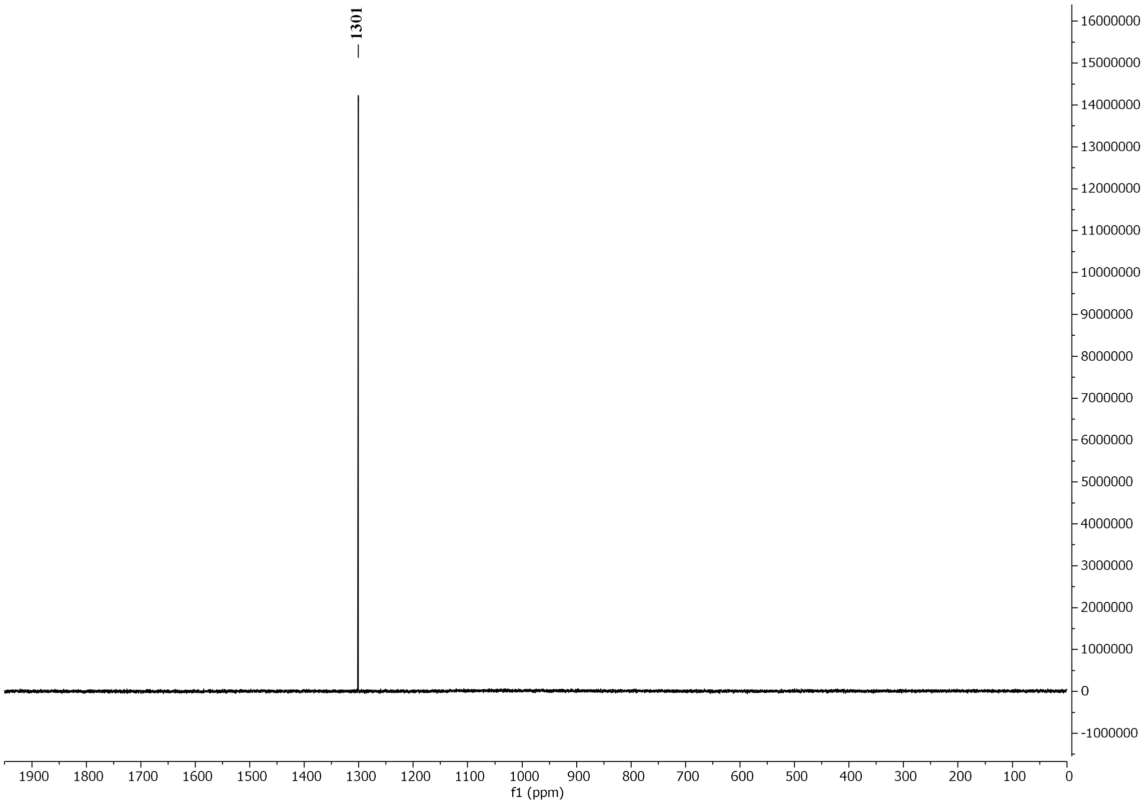

Figure S12. ^{77}Se-NMR spectrum of compound **SLT-4**.

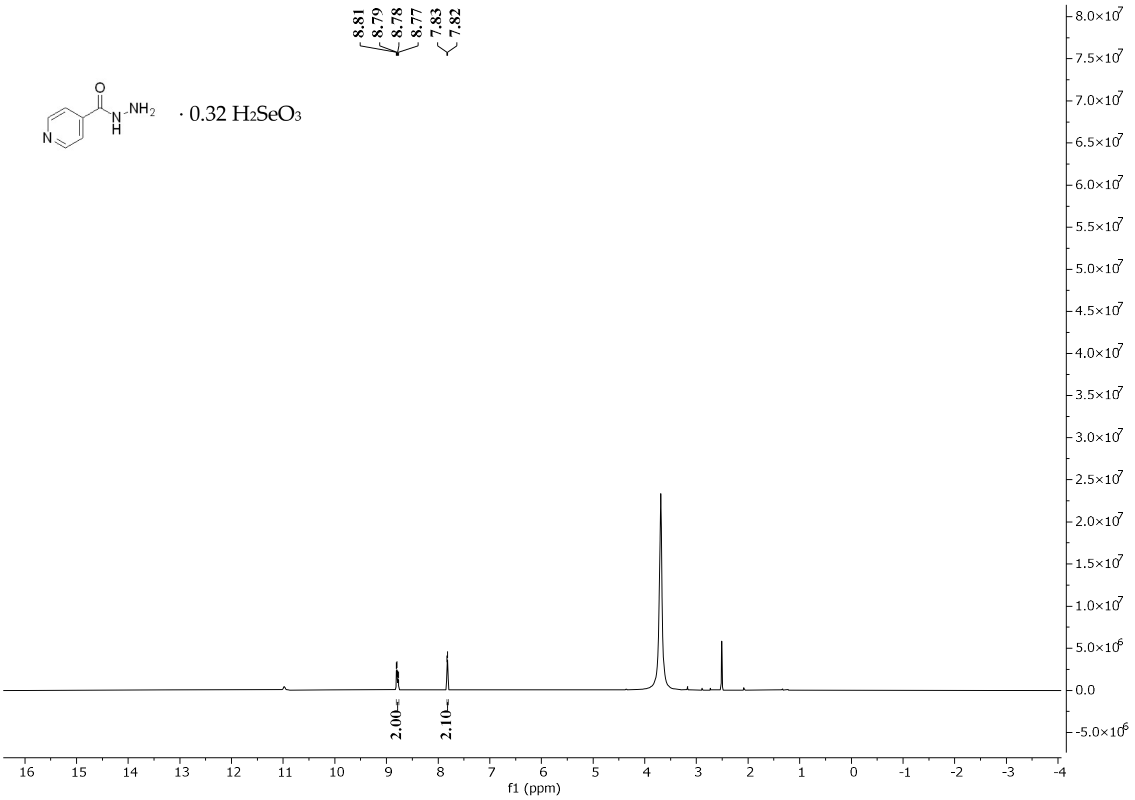

Figure S13. ^{1}H-NMR spectrum of compound **SLT-5**.

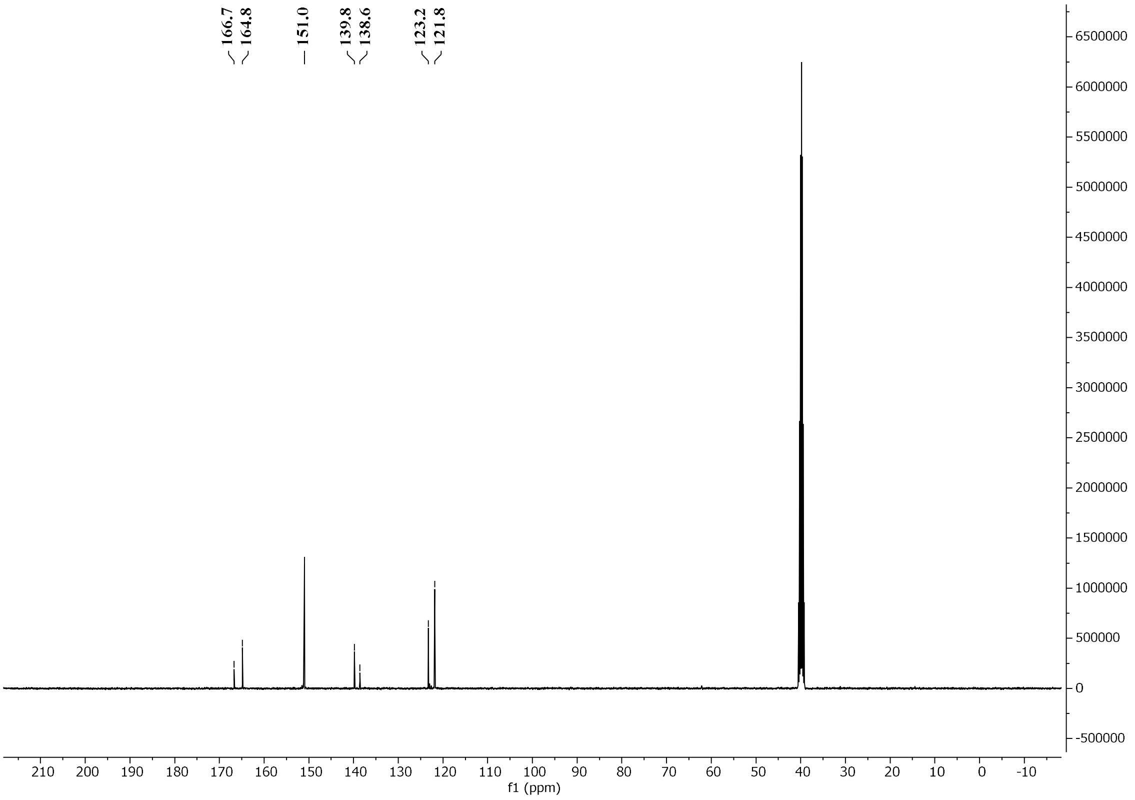

Figure S14. ^{13}C-NMR spectrum of compound **SLT-5**.

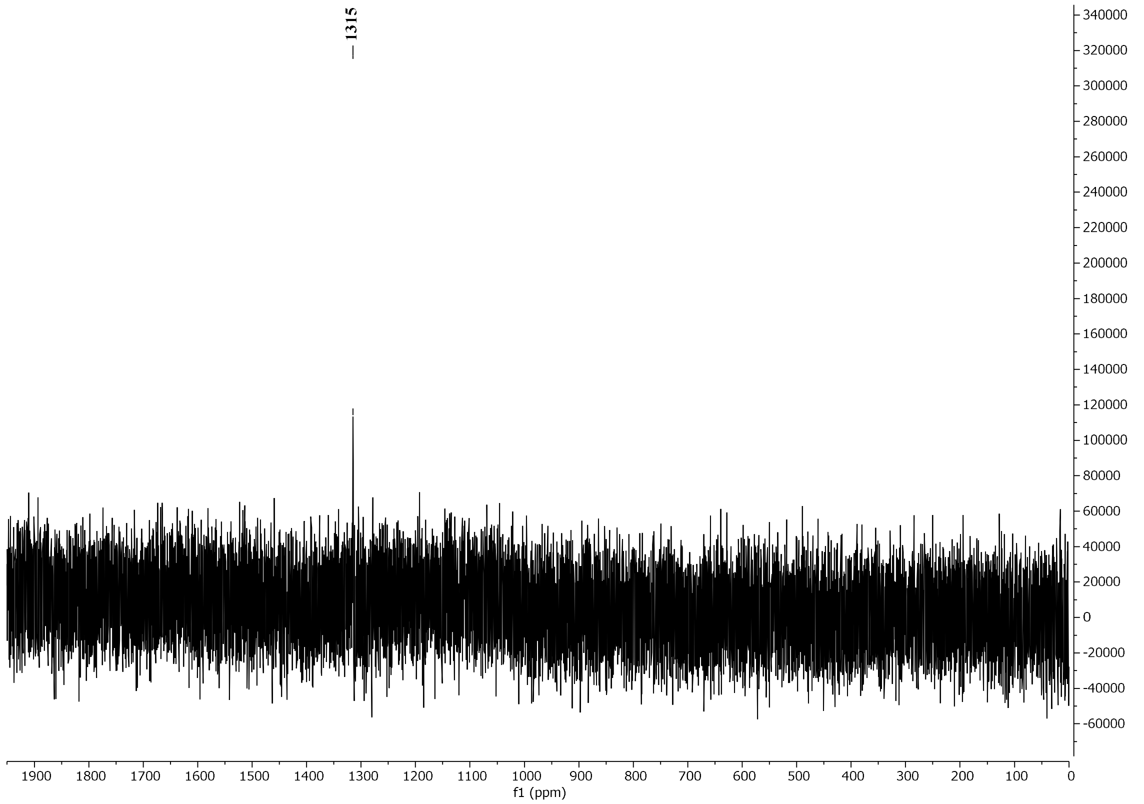

Figure S15. ^{77}Se-NMR spectrum of compound **SLT-5**.

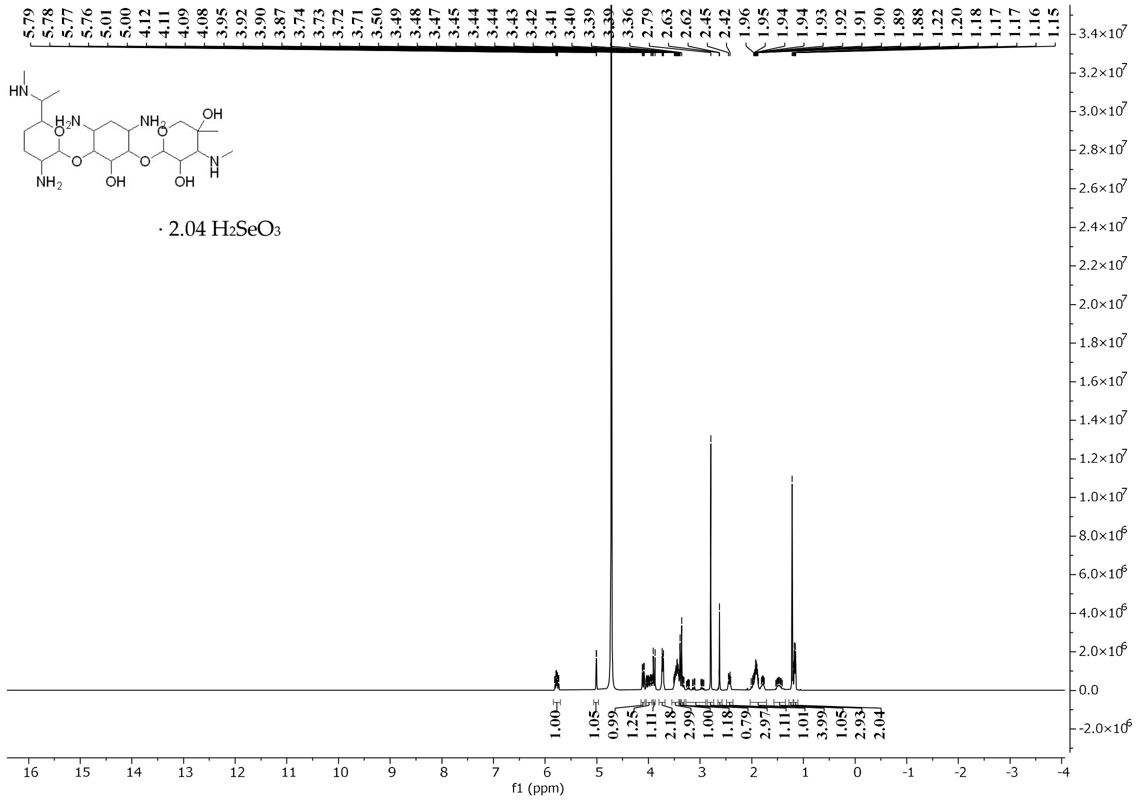

Figure S16. ^{1}H-NMR spectrum of compound **SLT-6**.

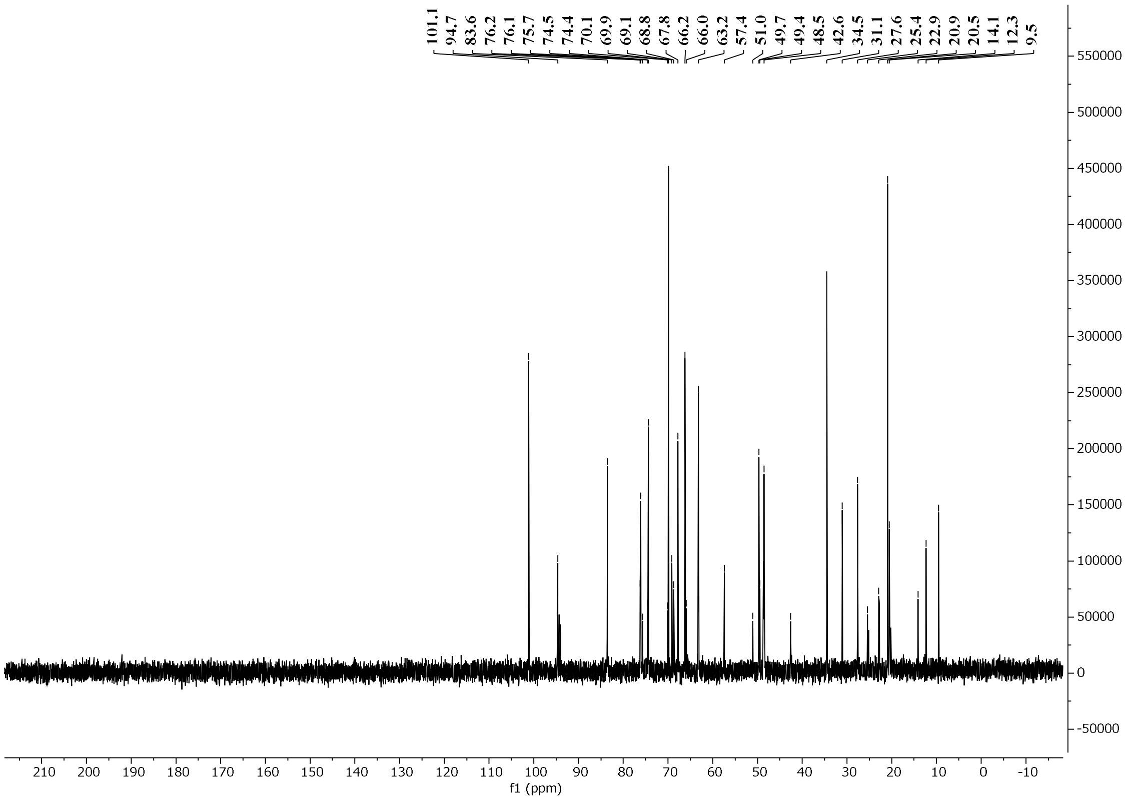

Figure S17. ^{13}C-NMR spectrum of compound **SLT-6**.

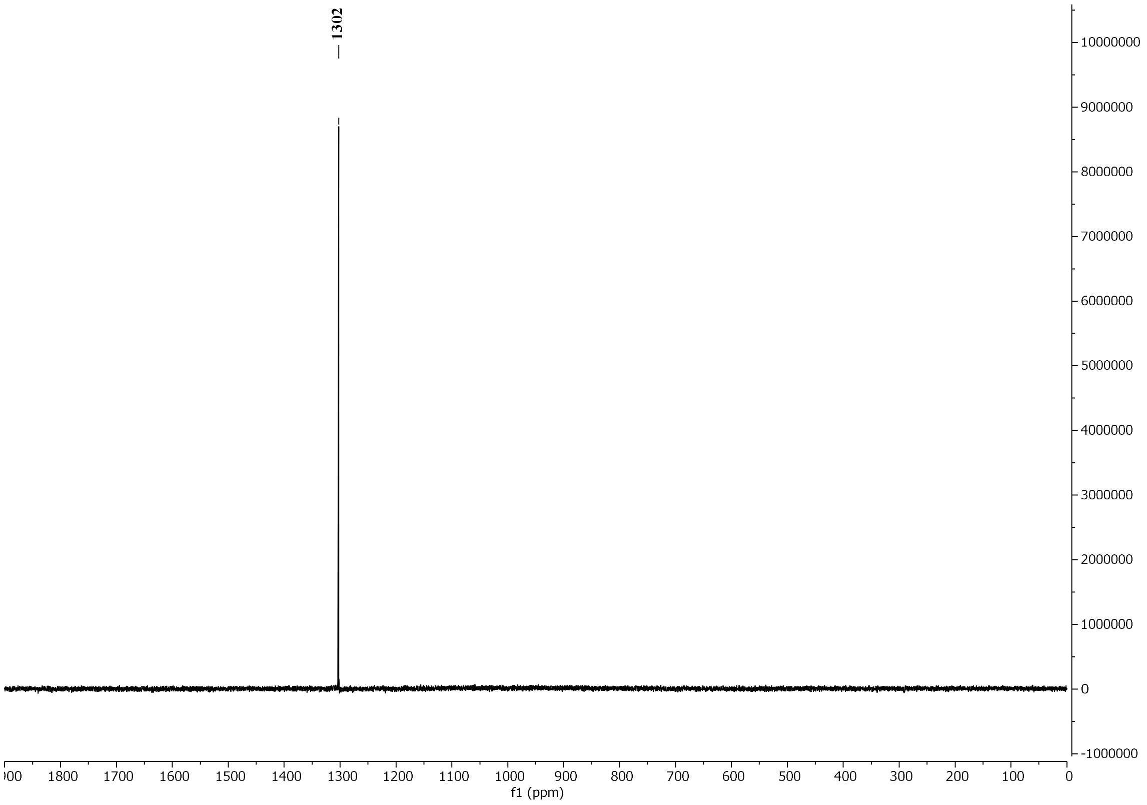

Figure S18. ^{77}Se-NMR spectrum of compound **SLT-6**.

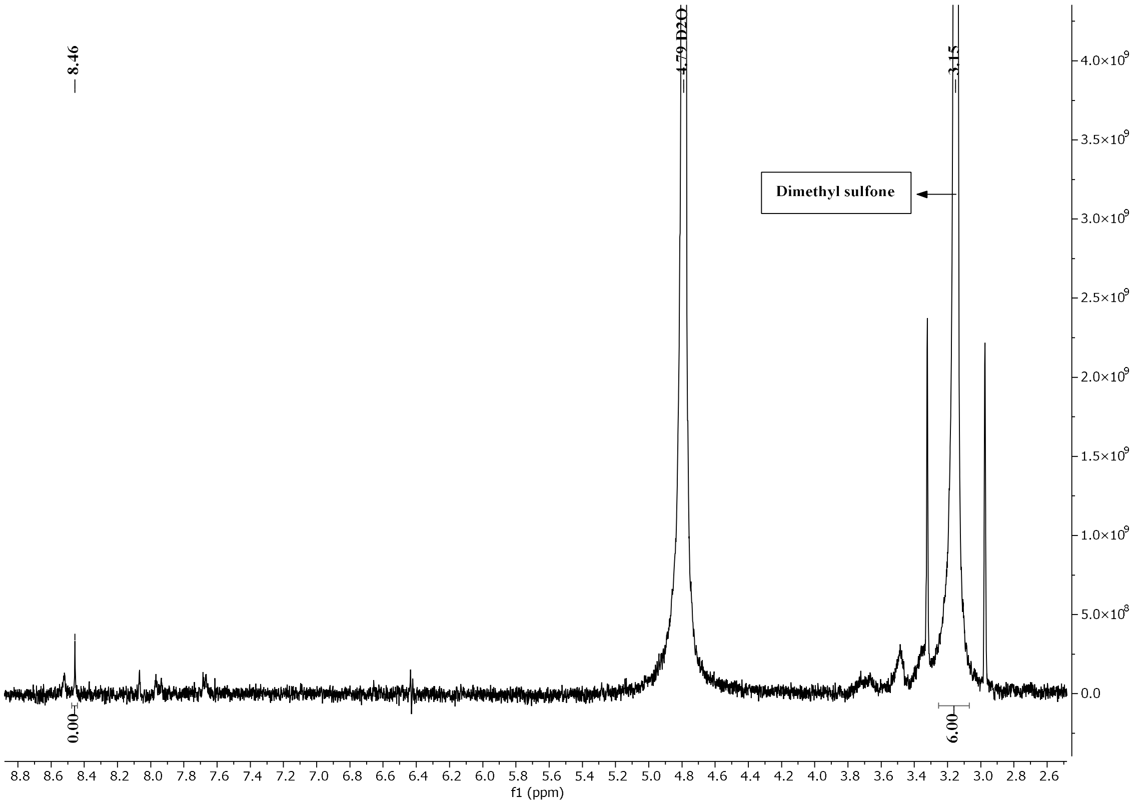

Figure S19. ^{1}H-NMR spectrum of compound **AB-1** and dimethyl sulfone.

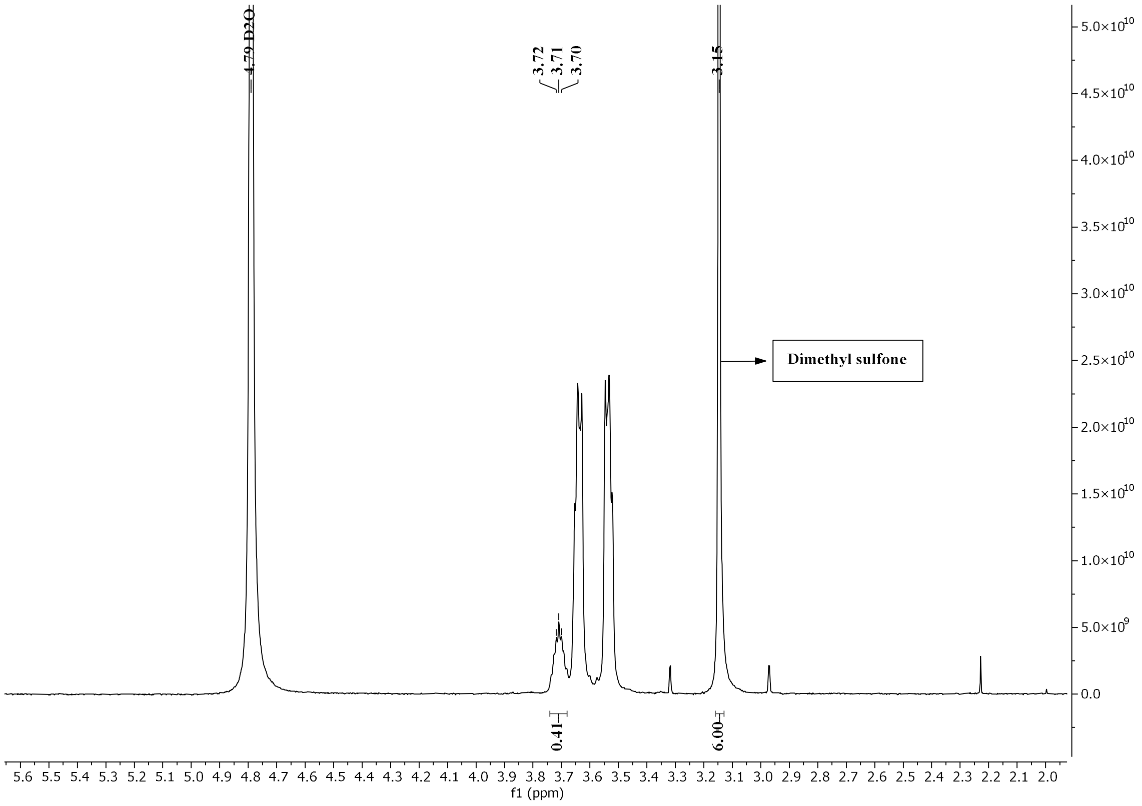

Figure S20. ^{1}H-NMR spectrum of compound **SLT-1** and dimethyl sulfone.

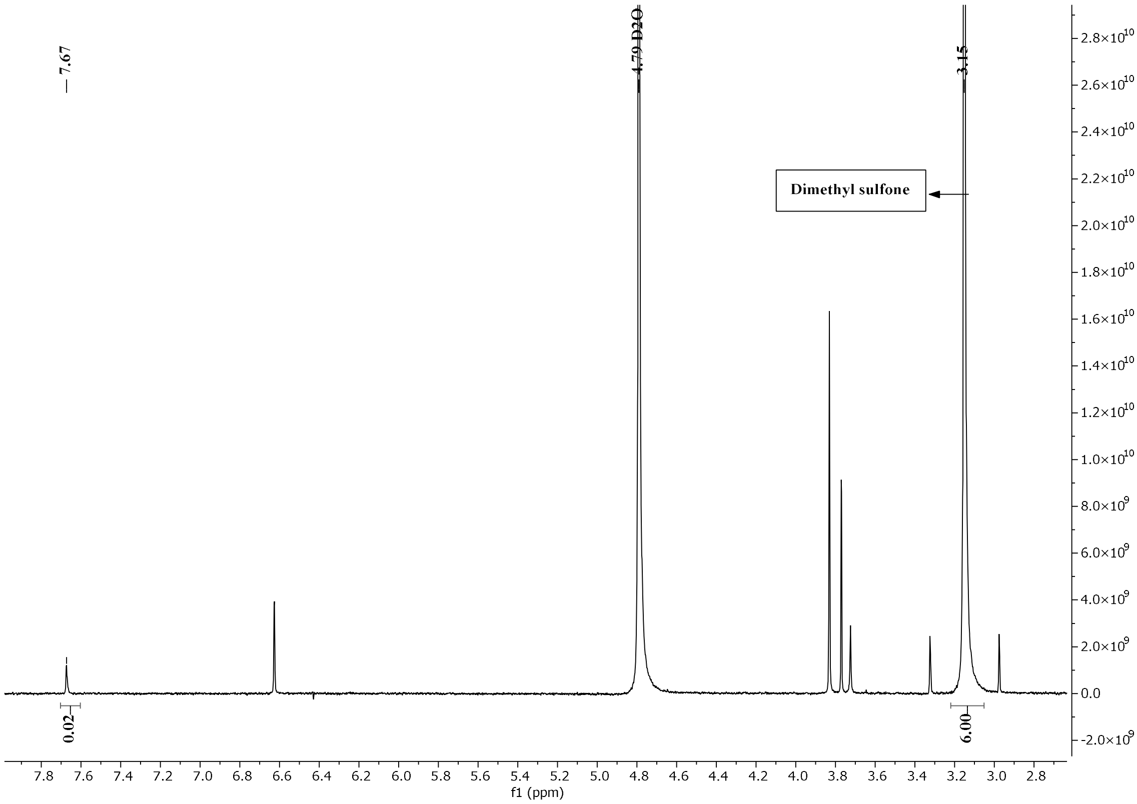

Figure S21. ^{1}H-NMR spectrum of compound **AB-2** and dimethyl sulfone.

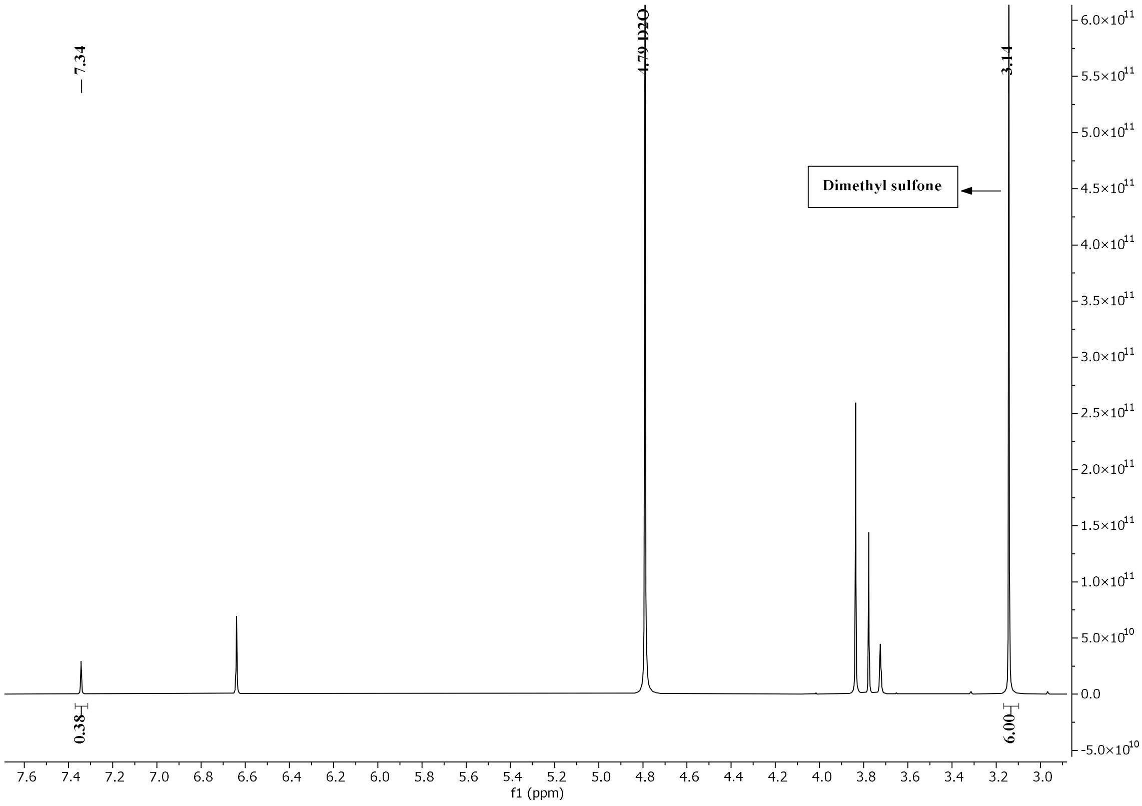

Figure S22. ^{1}H-NMR spectrum of compound **SLT-2** and dimethyl sulfone.

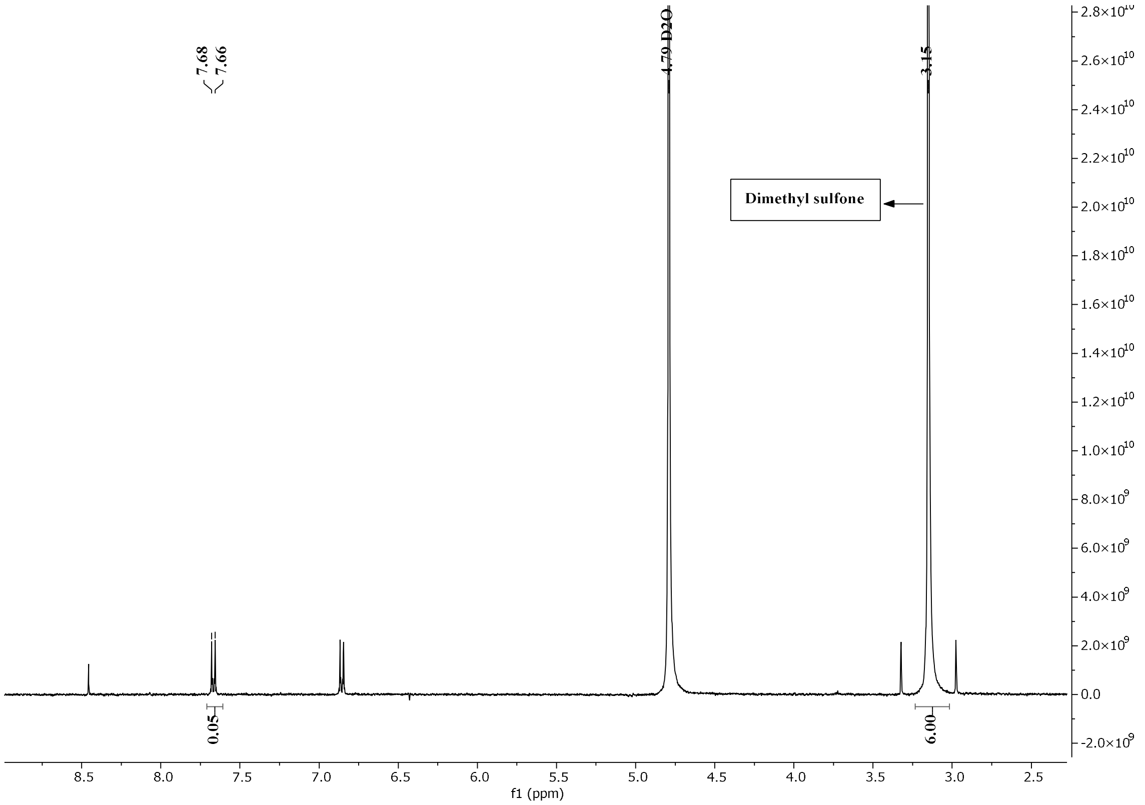

Figure S23. ^{1}H-NMR spectrum of compound **AB-3** and dimethyl sulfone.

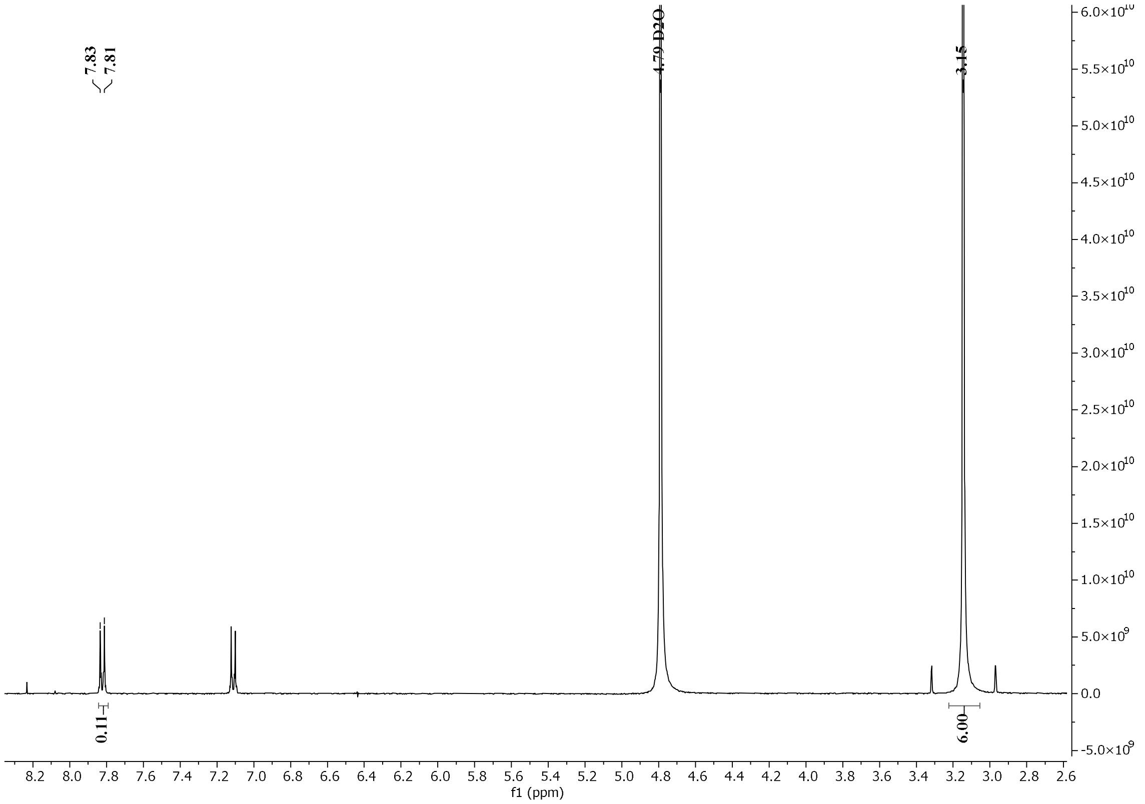

Figure S24. ^{1}H-NMR spectrum of compound **SLT-3** and dimethyl sulfone.

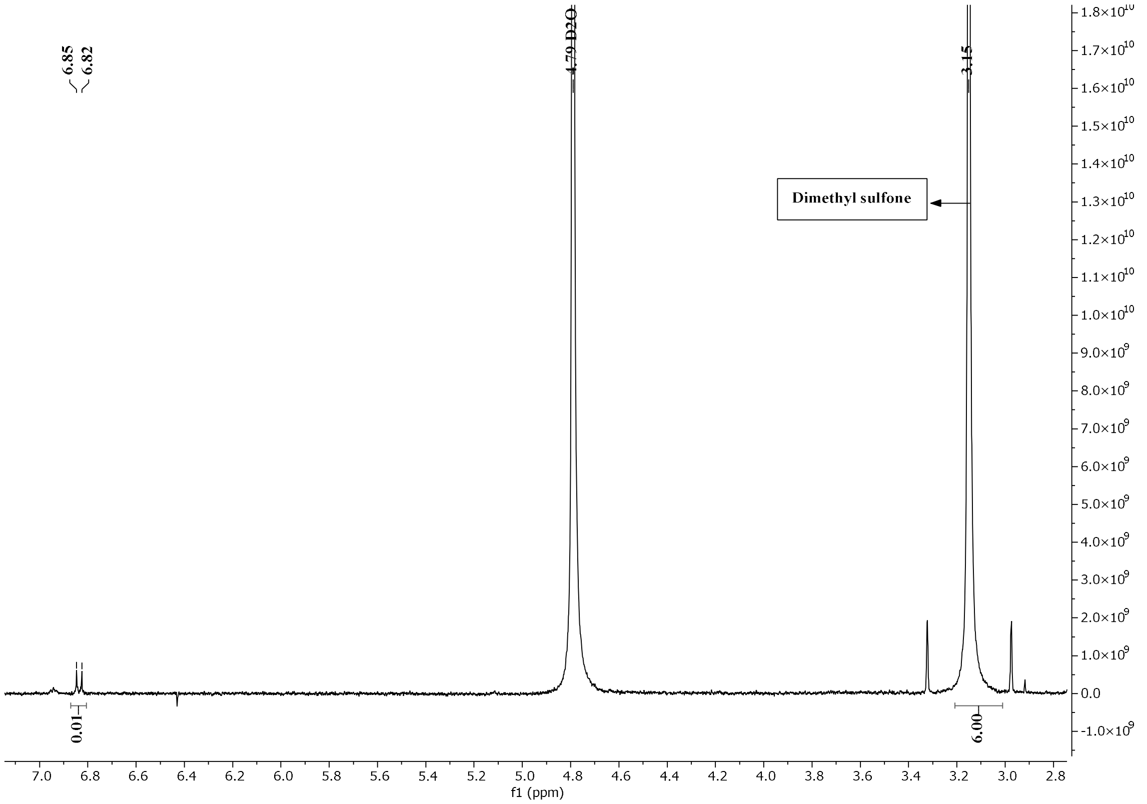

Figure S25. ^{1}H-NMR spectrum of compound **AB-4** and dimethyl sulfone.

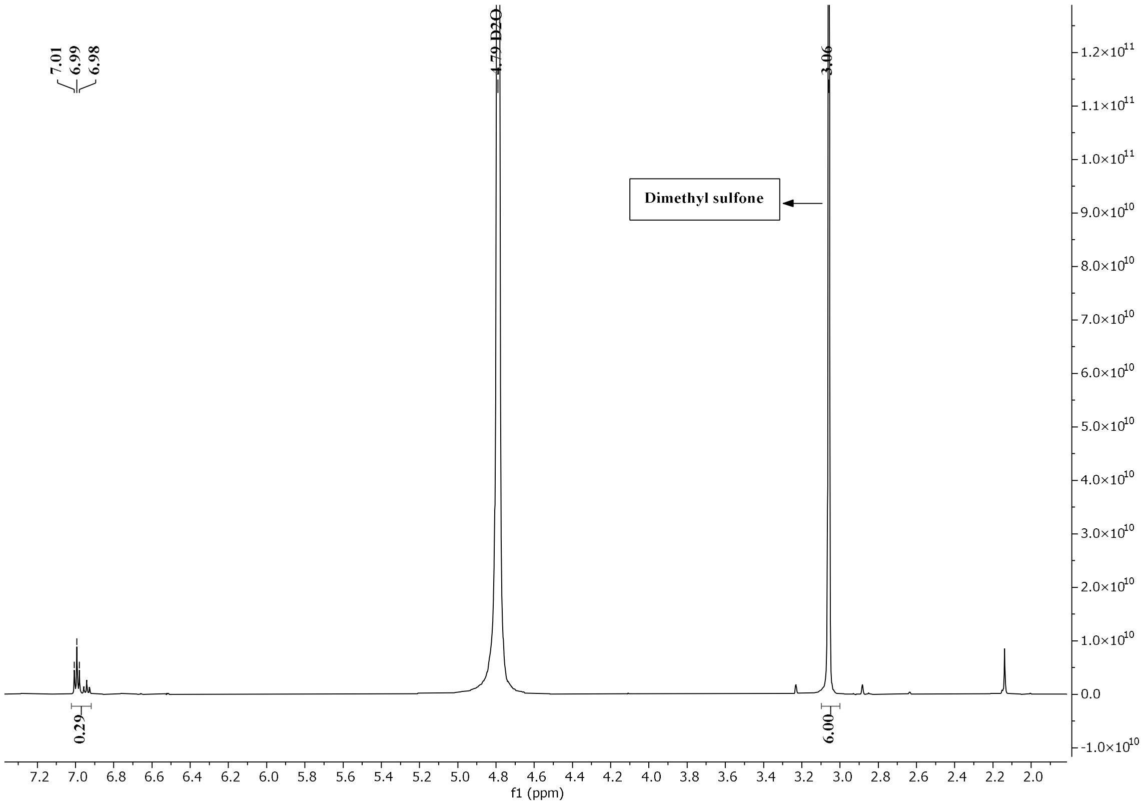

Figure S26. ^{1}H-NMR spectrum of compound **SLT-4** and dimethyl sulfone.

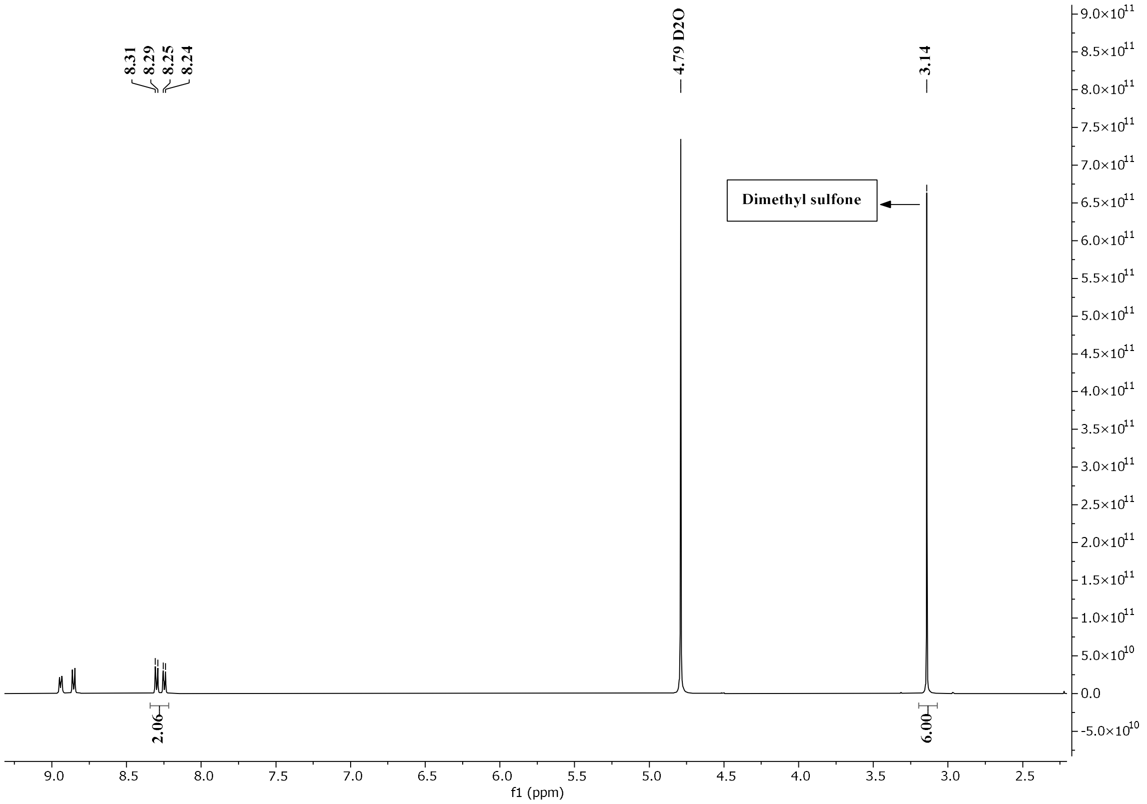

Figure S27. ^{1}H-NMR spectrum of compound **AB-5** and dimethyl sulfone.

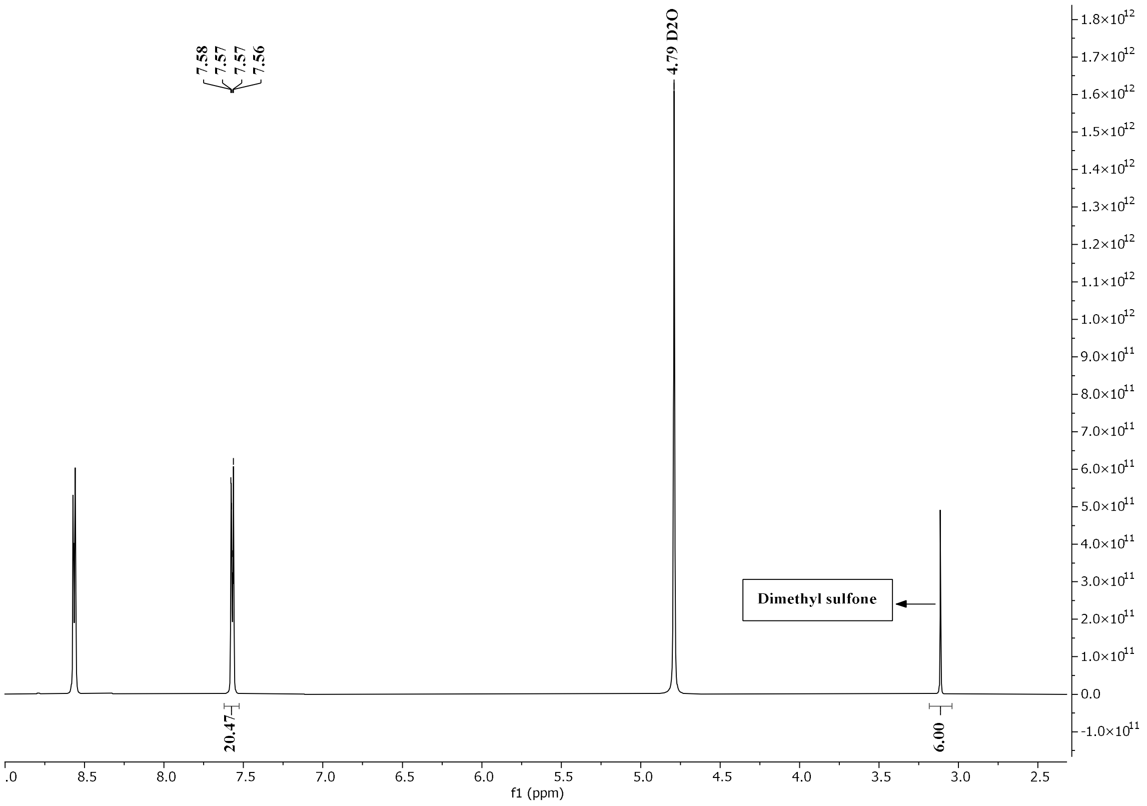

Figure S28. ^{1}H-NMR spectrum of compound **SLT-5** and dimethyl sulfone.

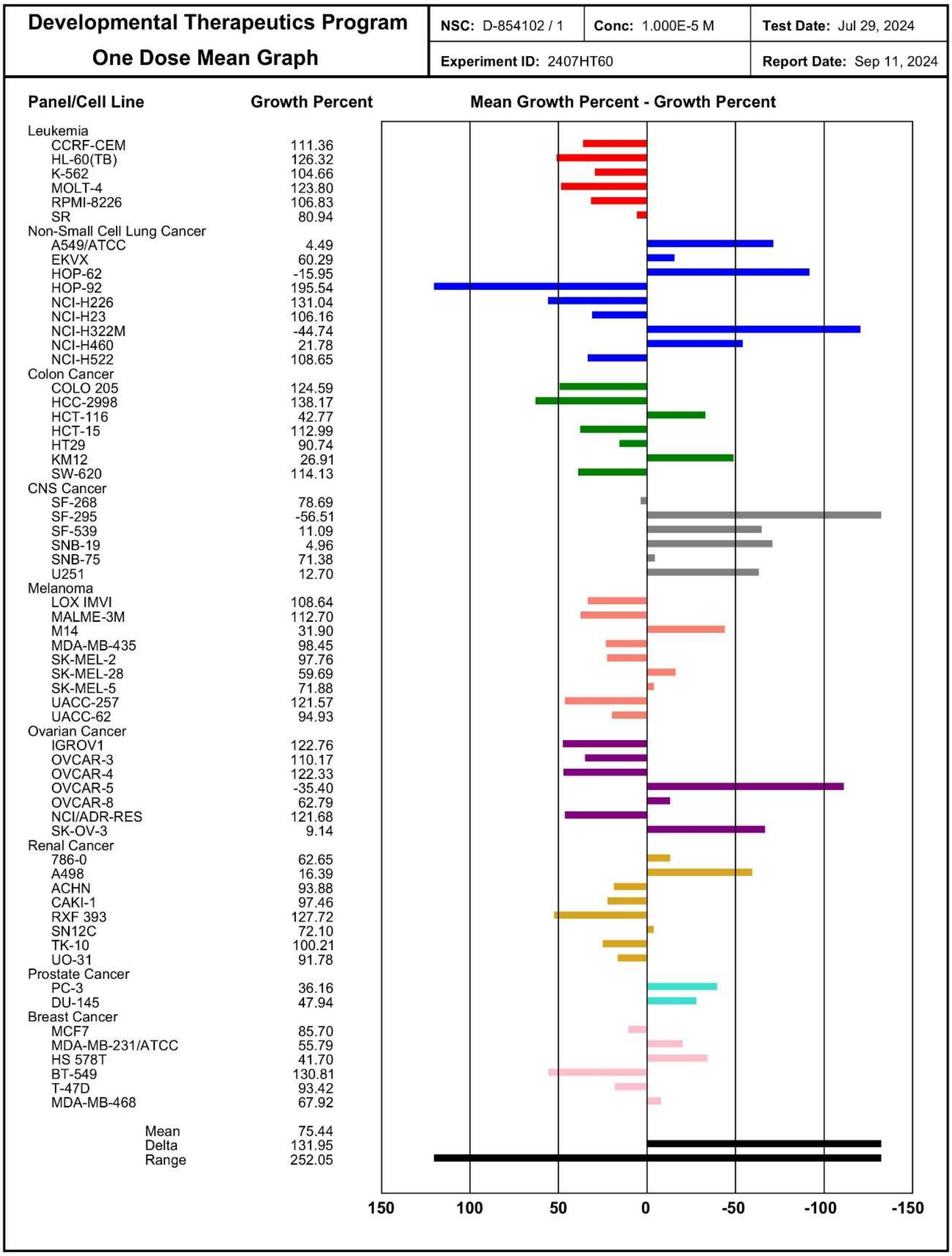

Figure S29. NCI-60 results at one dose (10 μM) of compound **SLT-1** (NSC 854102) after 48 h of treatment.

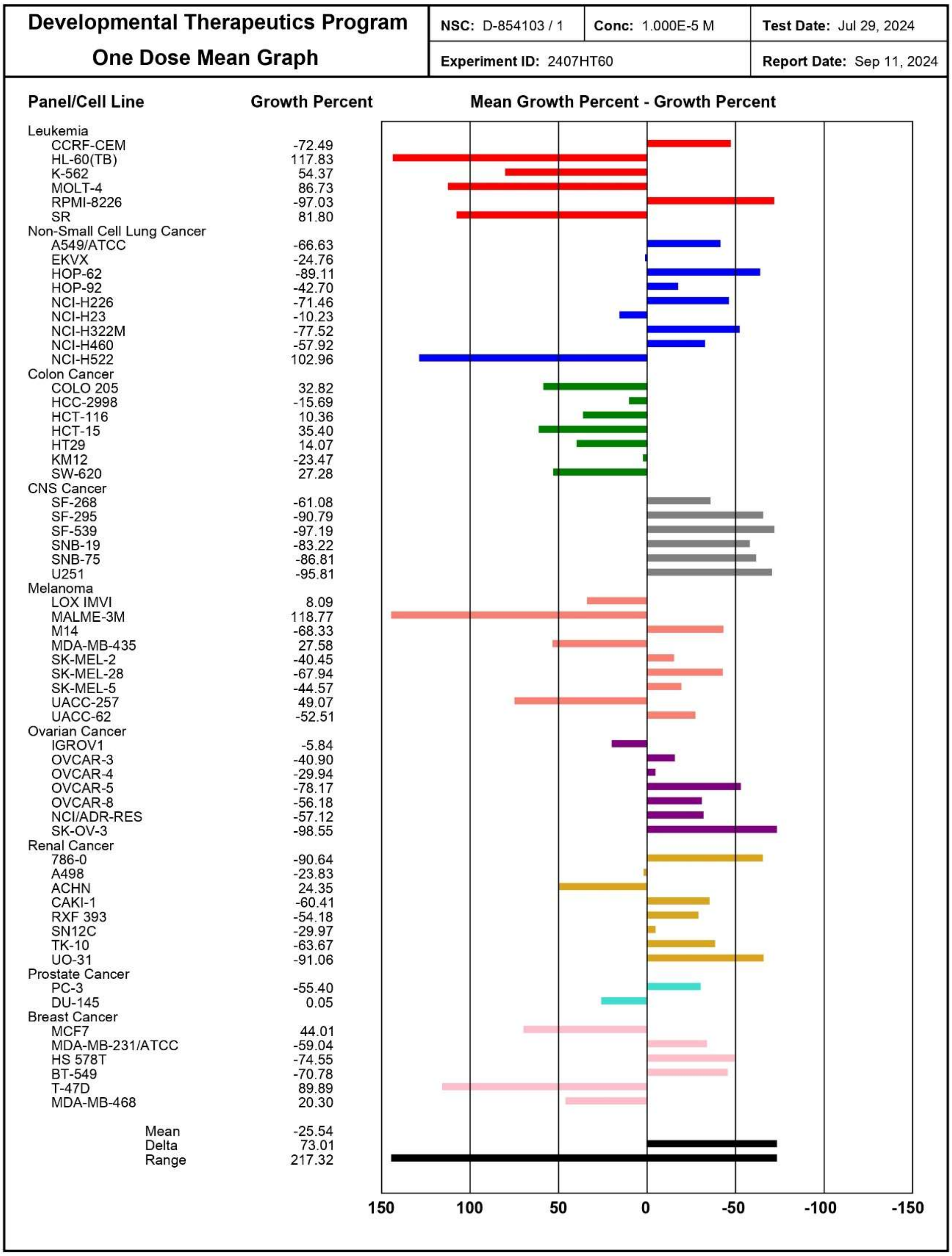

Figure S30. NCI-60 results at one dose (10 μM) of compound **SLT-2** (NSC 854103) after 48 h of treatment.

National Cancer Institute Developmental Therapeutics Program<br>In-Vitro Testing Results			
NSC : D - 854103 / 1	Experiment ID : 2410HT67	Test Type : HTS	Units : Molar
Report Date : December 4, 2024	Test Date : October 28, 2024	QNS :	MC :
COMI : SLT-2	Stain Reagent :	SSPL : 0ZVG	

			Log10 Concentration													
	Time		Mean Optical Densities					Percent Growth								
Panel/Cell Line	Zero	Ctrl	-8.0	-7.0	-6.0	-5.0	-4.0	-8.0	-7.0	-6.0	-5.0	-4.0	GI50	TGI	LC50
Leukemia															
CCRF-CEM	2.383	8.526	9.906	9.926	9.971	0.120	0.068	123	123	124	-95	-97	* 2.17E-6	* 3.68E-6	* 6.23E-6
HL-60(TB)	0.762	3.539	4.619	4.552	4.426	4.580	0.090	139	136	132	137	-88	* 2.44E-5	* 4.07E-5	* 6.77E-5
K-562	0.572	7.827	8.308	8.696	8.195	4.548	6.203	107	112	105	55	78	> 1.00E-4	> 1.00E-4	> 1.00E-4
MOLT-4	1.004	4.344	4.763	4.772	4.859	1.549	0.021	113	113	115	16	-98	* 4.57E-6	* 1.39E-5	* 3.80E-5
RPMI-8226	6.912	19.179	20.478	20.964	19.577	0.609	0.173	111	115	103	-91	-98	* 1.88E-6	* 3.40E-6	* 6.14E-6
SR	0.173	0.904	1.074	1.190	1.047	0.962	0.003	123	140	120	108	-98	* 1.92E-5	* 3.34E-5	* 5.84E-5
Non-Small Cell Lung Cancer															
A549/ATCC	0.672	5.520	5.328	5.541	5.300	0.255	0.377	96	100	96	-62	-44	* 1.94E-6	* 4.04E-6	.
EKVX	5.546	11.035	10.729	10.423	11.103	7.825	2.742	94	89	101	42	-51	* 7.22E-6	* 2.83E-5	* 9.86E-5
HOP-62	1.310	3.447	3.695	3.651	1.699	0.080	0.193	112	110	18	-94	-85	* 4.49E-7	* 1.45E-6	* 4.06E-6
HOP-92	5.399	13.855	13.841	14.370	13.659	14.375	7.757	100	106	98	106	28	* 5.22E-5	> 1.00E-4	> 1.00E-4
NCI-H226	9.434	12.254	12.679	13.026	13.247	1.007	2.121	115	127	135	-89	-78	* 2.40E-6	* 4.00E-6	* 6.68E-6
NCI-H23	3.807	13.460	14.007	14.595	14.157	5.972	1.846	106	112	107	22	-52	* 4.73E-6	* 2.01E-5	* 9.54E-5
NCI-H322M	3.247	8.307	9.278	9.154	7.088	1.552	1.380	119	117	76	-52	-58	* 1.59E-6	* 3.91E-6	* 9.61E-6
NCI-H460	1.152	16.510	17.438	17.603	16.198	0.926	0.786	106	107	98	-20	-32	* 2.56E-6	* 6.82E-6	> 1.00E-4
NCI-H522	5.459	11.960	11.796	12.152	12.398	12.562	3.314	98	103	107	109	-39	* 2.50E-5	* 5.44E-5	> 1.00E-4
Colon Cancer															
COLO 205	0.651	2.554	2.889	3.044	2.977	3.166	0.765	118	126	122	132	6	* 4.48E-5	> 1.00E-4	> 1.00E-4
HCC-2998	4.637	14.982	12.342	14.990	14.390	2.952	3.409	74	100	94	-36	-26	* 2.18E-6	* 5.27E-6	> 1.00E-4
HCT-116	0.718	4.642	4.790	4.909	4.968	0.957	0.741	104	107	108	6	1	* 3.72E-6	> 1.00E-4	> 1.00E-4
HCT-15	2.224	20.033	18.022	17.793	19.523	11.873	5.047	89	87	97	54	16	* 1.29E-5	> 1.00E-4	> 1.00E-4
HT29	0.858	6.729	6.518	6.906	7.045	1.695	2.156	96	103	105	14	22	* 4.05E-6	> 1.00E-4	> 1.00E-4
KM12	0.515	2.623	2.712	2.640	2.602	0.365	0.403	104	101	99	-29	-22	* 2.42E-6	* 5.94E-6	> 1.00E-4
SW-620	0.447	2.511	2.957	3.080	2.913	2.821	0.944	122	128	119	115	24	* 5.19E-5	> 1.00E-4	> 1.00E-4
CNS Cancer															
SF-268	1.150	3.512	3.237	3.257	3.345	0.335	0.584	88	89	93	-71	-49	* 1.83E-6	* 3.69E-6	.
SF-295	2.699	6.825	6.702	6.718	4.946	0.207	0.762	97	98	54	-92	-72	* 1.07E-6	* 2.35E-6	* 5.15E-6
SF-539	2.612	9.868	9.936	10.003	10.221	0.241	0.168	101	102	105	-91	-94	* 1.91E-6	* 3.44E-6	* 6.19E-6
SNB-19	0.764	1.537	1.767	1.885	1.281	0.152	0.535	130	145	67	-80	-30	* 1.30E-6	* 2.85E-6	.
SNB-75	2.121	4.196	3.929	3.939	3.869	0.051	0.484	87	88	84	-98	-77	* 1.54E-6	* 2.91E-6	* 5.48E-6
U251	0.840	4.125	4.286	4.224	3.781	0.077	0.291	105	103	90	-91	-65	* 1.66E-6	* 3.13E-6	* 5.93E-6
Melanoma															
LOX IMVI	0.569	3.939	4.226	4.027	4.161	0.971	0.889	109	103	107	12	9	* 3.96E-6	> 1.00E-4	> 1.00E-4
MALME-3M	7.908	14.227	13.968	13.922	14.033	14.754	10.418	96	96	97	108	40	* 7.10E-5	> 1.00E-4	> 1.00E-4
M14	3.018	11.834	12.419	12.820	12.671	2.668	1.569	107	111	109	-12	-48	* 3.10E-6	* 8.02E-6	> 1.00E-4
MDA-MB-435	1.295	4.207	4.364	4.743	4.761	2.408	2.382	105	118	119	38	37	* 7.15E-6	> 1.00E-4	> 1.00E-4
SK-MEL-2	1.837	4.972	4.650	4.682	4.861	2.058	0.730	90	91	96	7	-60	* 3.32E-6	* 1.28E-5	* 7.04E-5
SK-MEL-28	3.945	7.060	7.416	7.215	7.284	1.585	2.888	111	105	107	-60	-27	* 2.20E-6	* 4.38E-6	.
SK-MEL-5	3.148	12.690	12.512	11.941	12.329	1.806	3.160	98	92	96	-43	-2	* 2.15E-6	* 4.93E-6	> 1.00E-4
UACC-257	3.162	6.608	6.998	7.116	7.254	6.729	3.651	111	115	119	104	14	* 3.97E-5	> 1.00E-4	> 1.00E-4
UACC-62	0.869	3.284	3.266	3.438	3.465	0.652	2.010	100	106	108	-25	47	* 2.74E-6	.	> 1.00E-4
Ovarian Cancer															
IGROV1	1.064	4.205	4.607	4.621	4.599	0.764	0.914	113	113	113	-28	-14	* 2.79E-6	* 6.31E-6	> 1.00E-4
OVCAR-3	3.350	10.365	10.249	10.795	11.313	6.228	3.995	98	106	114	41	9	* 7.54E-6	> 1.00E-4	> 1.00E-4
OVCAR-4	4.819	9.106	8.754	9.492	8.940	8.212	4.749	92	109	97	79	-1	* 2.30E-5	* 9.59E-5	> 1.00E-4
OVCAR-5	4.584	14.398	14.485	14.668	15.919	2.058	2.033	101	103	115	-55	-56	* 2.42E-6	* 4.75E-6	* 9.33E-6
OVCAR-8	0.817	4.767	4.808	4.698	4.959	0.513	0.117	101	98	105	-37	-86	* 2.43E-6	* 5.47E-6	* 1.84E-5
NCI/ADR-RES	3.596	12.384	13.288	13.280	13.794	4.816	1.578	110	110	116	14	-56	* 4.43E-6	* 1.58E-5	* 8.18E-5
SK-OV-3	2.453	7.029	7.372	7.135	7.196	0.317	1.713	107	102	104	-87	-30	* 1.91E-6	* 3.49E-6	.
Renal Cancer															
786-0	2.430	5.978	6.045	5.650	5.618	0.160	0.201	104	96	95	-93	-92	* 1.73E-6	* 3.19E-6	* 5.88E-6
A498	3.091	14.250	14.705	14.741	14.396	2.868	2.559	104	104	101	-7	-17	* 2.97E-6	* 8.58E-6	> 1.00E-4
ACHN	1.436	5.807	5.980	6.132	6.158	2.459	1.853	104	107	108	23	10	* 4.85E-6	> 1.00E-4	> 1.00E-4
CAKI-1	0.927	5.749	5.614	5.720	5.720	0.932	1.035	97	99	99	-1	2	* 3.12E-6	.	> 1.00E-4
RXF 393	2.153	.	.	.	.	.	.	.	.	.	.	.	.	.	.
SN12C	1.356	3.860	3.660	3.601	3.789	0.400	0.517	92	90	97	-70	-62	* 1.91E-6	* 3.80E-6	* 7.55E-6
TK-10	2.146	7.289	8.523	8.080	8.364	4.213	3.791	124	115	121	40	32	* 7.52E-6	> 1.00E-4	> 1.00E-4
UO-31	1.458	.	.	.	.	.	.	.	.	.	.	.	.	.	.
Prostate Cancer															
PC-3	5.569	.	.	.	.	.	.	.	.	.	.	.	.	.	.
DU-145	1.963	4.648	4.826	4.854	4.962	0.881	0.765	107	108	112	-55	-61	* 2.34E-6	* 4.67E-6	* 9.32E-6
Breast Cancer															
MCF7	2.853	14.614	13.481	14.422	15.539	10.587	6.149	90	98	108	66	28	* 2.62E-5	> 1.00E-4	> 1.00E-4
MDA-MB-231/ATCC	4.387	15.617	16.031	15.707	15.931	2.770	2.055	104	101	103	-37	-53	* 2.39E-6	* 5.45E-6	* 6.41E-5
HS 578T	1.737	3.359	3.109	3.181	3.056	0.212	0.480	85	89	81	-88	-72	* 1.53E-6	* 3.02E-6	* 5.97E-6
BT-549	8.085	.	.	.	.	.	.	.	.	.	.	.	.	.	.
T-47D	5.448	10.078	10.146	9.880	10.412	10.107	6.188	102	96	107	101	16	* 3.97E-5	> 1.00E-4	> 1.00E-4
MDA-MB-468	4.562	7.434	7.367	7.283	7.469	7.492	6.839	98	95	102	102	79	> 1.00E-4	> 1.00E-4	> 1.00E-4

Figure S31. NCI's DTP dose-response report for compound **SLT-2**. GI_{50}, TGI and LC_{50} values (expressed as molarity).

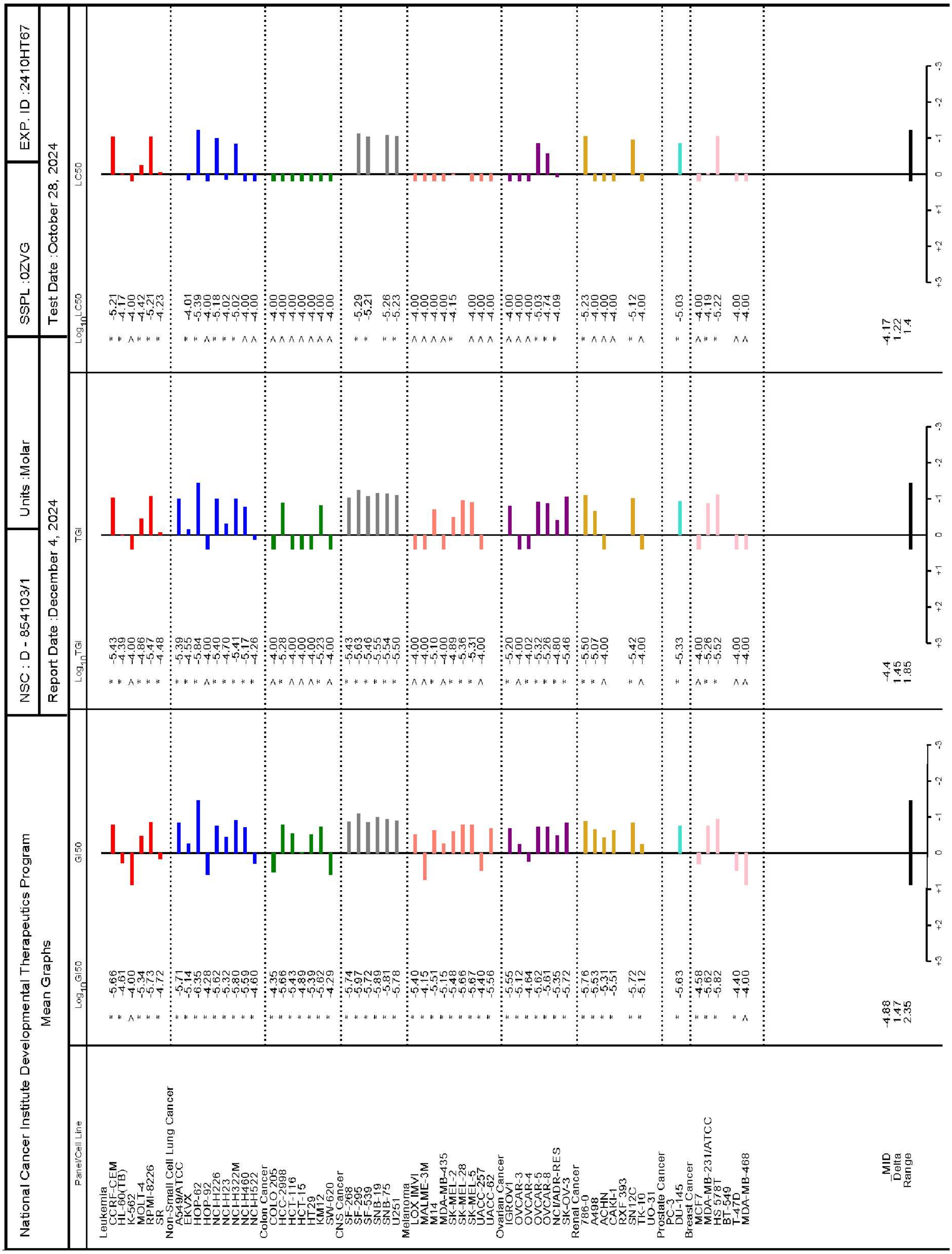

Figure S32. NCI's DTP dose-response report for compound **SLT-2**. Mean graphs of GI_{50}, TGI and LC_{50} values.

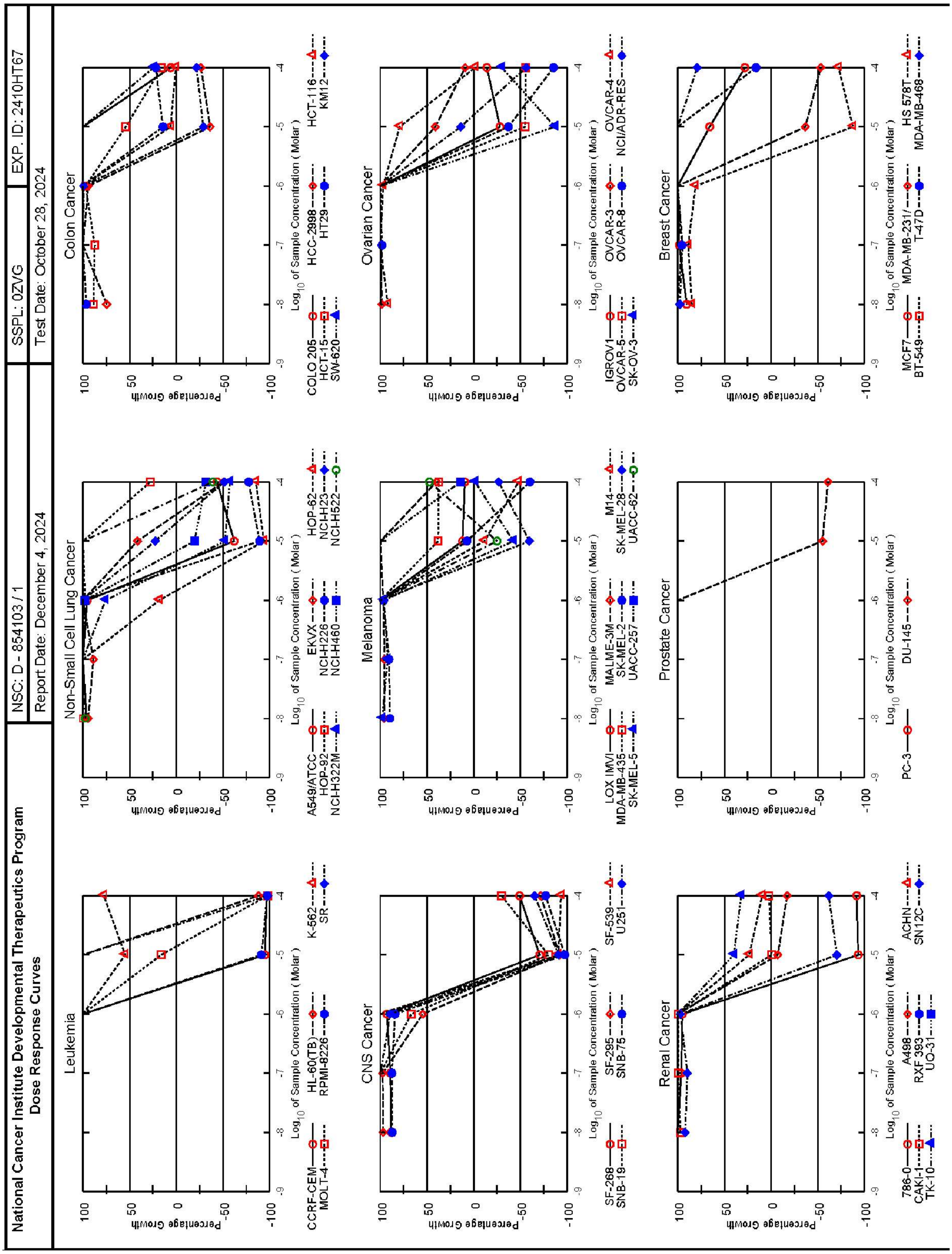

Figure S33. Dose-response curves for **SLT-2.**

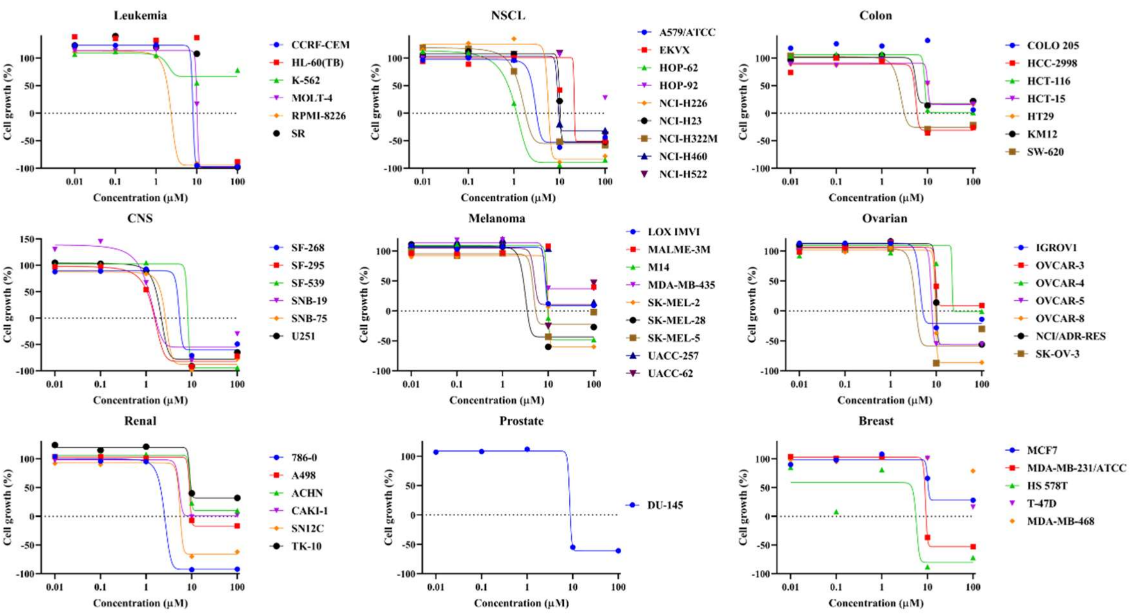

Figure S34. Dose-response curves of **SLT-2** in all the cell lines present in the NCI panel.

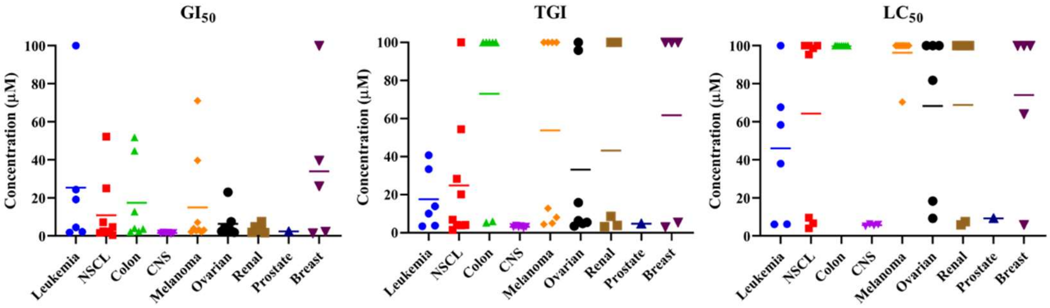

Figure S35. Mean graphs of the GI_{50}, TGI, and LC_{50} values of **SLT-2.**

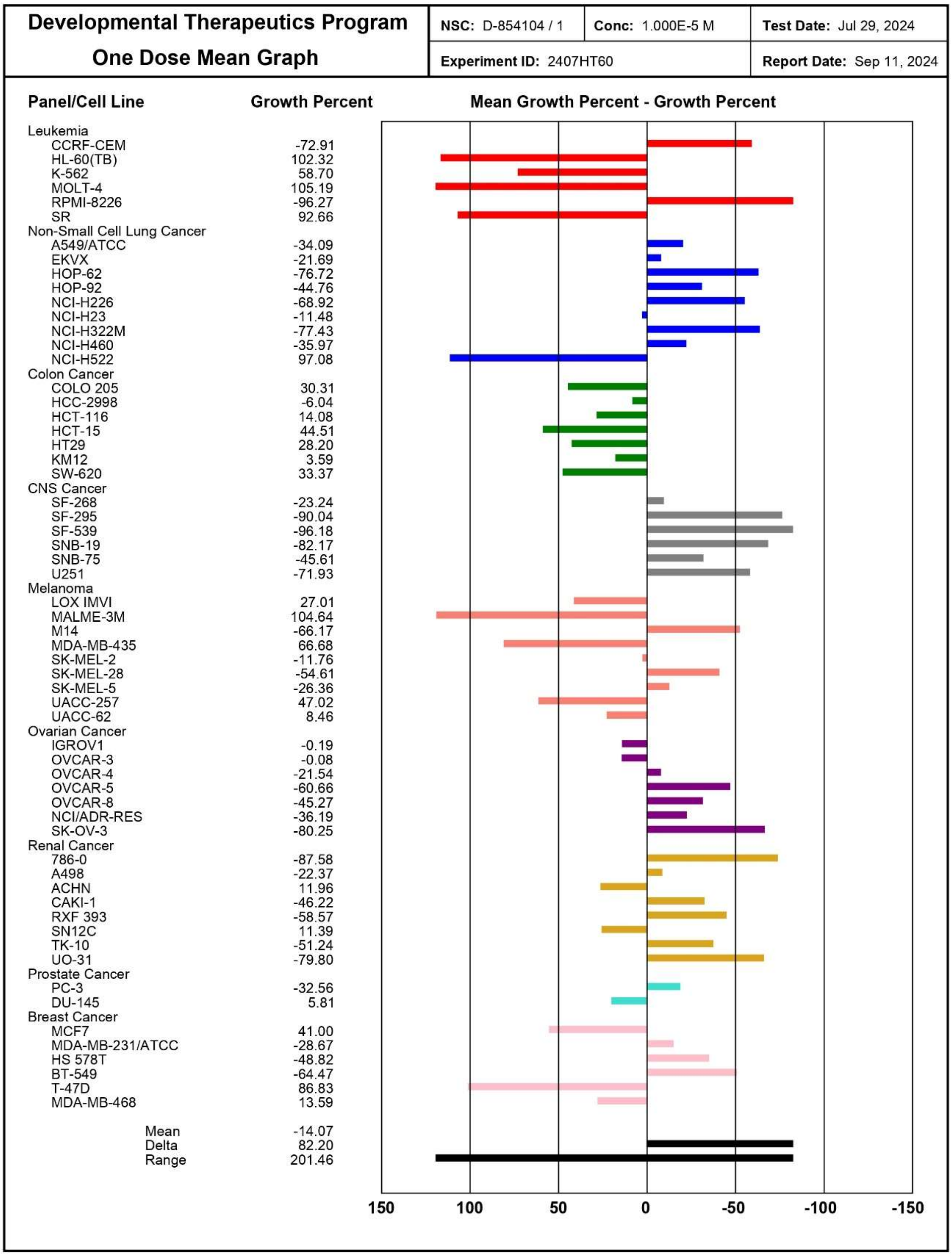

Figure S31. NCI-60 results at one dose (10 μM) of compound **SLT-3** (NSC 854104) after 48 h of treatment.

National Cancer Institute Developmental Therapeutics Program
In-Vitro Testing Results

NSC : D - 854104 / 1	Experiment ID : 2410HT67	Test Type : HTS	Units : Molar
Report Date : December 4, 2024	Test Date : October 28, 2024	QNS :	MC :
COMI : SLT-6	Stain Reagent :	SSPL : 0ZVG	

			Log10 Concentration														
	Time		Mean Optical Densities					Percent Growth									
Panel/Cell Line	Zero	Ctrl	-8.0	-7.0	-6.0	-5.0	-4.0	-8.0	-7.0	-6.0	-5.0	-4.0		GI50		TGI	LC50
**Leukemia**																	
CCRF-CEM	2.383	8.526	10.070	9.846	10.047	0.035	0.073	125	122	125	-98	-97		* 2.16E-6		* 3.62E-6	* 6.06E-6
HL-60(TB)	0.762	3.539	4.468	4.424	4.589	4.301	0.056	133	132	138	128	-93		* 2.25E-5		* 3.79E-5	* 6.40E-5
K-562	0.572	7.827	8.960	8.407	8.429	4.976	5.880	116	108	108	61	73		> 1.00E-4		> 1.00E-4	> 1.00E-4
MOLT-4	1.004	4.344	4.812	4.727	4.776	0.751	0.005	114	111	113	-25	-100		* 2.86E-6		* 6.57E-6	* 2.16E-5
RPMI-8226	6.912	19.179	20.960	21.248	20.792	0.336	0.044	115	117	113	-95	-99		* 2.01E-6		* 3.49E-6	* 6.07E-6
SR	0.173	0.904	1.247	1.097	1.045	0.626	0.001	147	126	120	62	-99		* 1.19E-5		* 2.42E-5	* 4.94E-5
**Non-Small Cell Lung Cancer**																	
A549/ATCC	0.672	5.520	5.475	5.453	4.628	0.458	0.469	99	99	82	-32	-30		* 1.90E-6		* 5.24E-6	> 1.00E-4
EKVX	5.546	11.035	10.490	10.478	10.603	5.543	3.977	90	90	92	-0	-28		* 2.86E-6		* 9.99E-6	> 1.00E-4
HOP-62	1.310	3.447	3.495	3.663	1.329	0.173	0.259	102	110	1	-87	-80		* 3.55E-7		* 1.02E-6	* 3.80E-6
HOP-92	5.399	13.855	14.483	14.442	14.574	14.084	7.616	107	107	109	103	26		* 4.89E-5		> 1.00E-4	> 1.00E-4
NCI-H226	9.434	12.254	12.835	12.986	12.361	1.742	2.389	121	126	104	-82	-75		* 1.95E-6		* 3.63E-6	* 6.76E-6
NCI-H23	3.807	13.460	13.850	14.123	14.522	3.886	2.470	104	107	111	1	-35		* 3.58E-6		* 1.04E-5	> 1.00E-4
NCI-H322M	3.247	8.307	9.097	9.264	4.581	1.616	1.325	116	119	26	-50	-59		* 5.55E-7		* 2.21E-6	* 9.93E-6
NCI-H460	1.152	16.510	17.555	17.784	11.681	0.929	0.715	107	108	69	-19	-38		* 1.63E-6		* 6.03E-6	> 1.00E-4
NCI-H522	5.459	11.960	11.687	12.221	11.685	11.471	4.428	96	104	96	92	-19		* 2.41E-5		* 6.77E-5	> 1.00E-4
**Colon Cancer**																	
COLO 205	0.651	2.554	2.882	2.984	3.101	2.738	0.962	117	123	129	110	16		* 4.37E-5		> 1.00E-4	> 1.00E-4
HCC-2998	4.637	14.982	12.720	11.010	13.357	4.409	2.992	78	62	84	-5	-36		* 2.42E-6		* 8.81E-6	> 1.00E-4
HCT-116	0.718	4.642	4.736	4.976	4.540	0.975	0.756	102	109	97	7	1		* 3.32E-6		> 1.00E-4	> 1.00E-4
HCT-15	2.224	20.033	18.739	17.758	19.521	9.785	8.435	93	87	97	43	35		* 7.30E-6		> 1.00E-4	> 1.00E-4
HT29	0.858	6.729	6.940	6.915	6.920	3.060	2.835	104	103	103	38	34		* 6.46E-6		> 1.00E-4	> 1.00E-4
KM12	0.515	2.623	2.620	2.699	2.721	0.563	0.376	100	104	104	-2	-27		* 3.25E-6		* 9.58E-6	> 1.00E-4
SW-620	0.447	2.511	2.848	2.907	2.789	1.805	0.838	116	119	113	66	19		* 2.17E-5		> 1.00E-4	> 1.00E-4
**CNS Cancer**																	
SF-268	1.150	3.512	3.556	3.663	3.327	0.735	0.930	102	106	92	-36	-19		* 2.14E-6		* 5.24E-6	> 1.00E-4
SF-295	2.699	6.825	6.650	6.796	3.067	0.483	0.721	96	99	9	-82	-73		* 3.51E-7		* 1.25E-6	* 4.44E-6
SF-539	2.612	9.868	9.658	9.506	9.827	0.251	0.121	97	95	100	-90	-95		* 1.83E-6		* 3.35E-6	* 6.13E-6
SNB-19	0.764	1.537	1.777	1.936	1.269	0.373	0.670	131	151	65	-51	-12		* 1.35E-6		* 3.64E-6	.
SNB-75	2.121	4.196	3.970	3.997	4.093	0.224	0.298	89	90	95	-89	-86		* 1.75E-6		* 3.28E-6	* 6.11E-6
U251	0.840	4.125	4.542	4.316	2.666	0.248	0.383	113	106	56	-70	-54		* 1.11E-6		* 2.76E-6	* 6.87E-6
**Melanoma**																	
LOX IMVI	0.569	3.939	4.146	4.080	4.102	1.274	1.359	106	104	105	21	23		* 4.50E-6		> 1.00E-4	> 1.00E-4
MALME-3M	7.908	14.227	13.970	14.903	14.752	14.413	10.760	96	111	109	103	45		* 8.29E-5		> 1.00E-4	> 1.00E-4
M14	3.018	11.834	12.323	12.935	13.329	2.469	1.040	106	112	117	-18	-66		* 3.13E-6		* 7.34E-6	* 4.70E-5
MDA-MB-435	1.295	4.207	4.602	4.793	4.640	3.206	1.884	114	120	115	66	20		* 2.21E-5		> 1.00E-4	> 1.00E-4
SK-MEL-2	1.837	4.972	5.045	5.320	4.746	1.471	0.931	102	111	93	-20	-49		* 2.41E-6		* 6.67E-6	> 1.00E-4
SK-MEL-28	3.945	7.060	7.331	7.133	7.350	2.538	3.573	109	102	109	-36	-9		* 2.57E-6		* 5.68E-6	> 1.00E-4
SK-MEL-5	3.148	12.690	11.196	12.990	11.202	3.464	2.080	84	103	84	3	-34		* 2.66E-6		* 1.23E-5	> 1.00E-4
UACC-257	3.162	6.608	6.876	7.273	7.055	5.041	3.593	108	119	113	55	12		* 1.28E-5		> 1.00E-4	> 1.00E-4
UACC-62	0.869	3.284	3.649	3.637	3.686	1.264	1.985	116	115	117	16	46		* 4.62E-6		> 1.00E-4	> 1.00E-4
**Ovarian Cancer**																	
IGROV1	1.064	4.205	4.302	4.571	4.301	1.023	1.231	103	112	103	-4	5		* 3.14E-6		.	> 1.00E-4
OVCAR-3	3.350	10.365	10.654	11.384	9.552	4.424	4.489	104	114	88	15	16		* 3.34E-6		> 1.00E-4	> 1.00E-4
OVCAR-4	4.819	9.106	8.921	8.993	8.560	8.036	5.366	97	98	90	74	12		* 2.40E-5		> 1.00E-4	> 1.00E-4
OVCAR-5	4.584	14.398	14.386	14.703	13.687	2.475	1.941	100	103	93	-46	-58		* 2.03E-6		* 4.66E-6	* 2.20E-5
OVCAR-8	0.817	4.767	4.749	4.935	4.809	0.344	0.257	100	104	101	-58	-68		* 2.10E-6		* 4.32E-6	* 8.92E-6
NCI/ADR-RES	3.596	12.384	12.965	13.481	13.338	2.852	1.685	107	112	111	-21	-53		* 2.90E-6		* 6.96E-6	* 8.01E-5
SK-OV-3	2.453	7.029	6.443	6.733	6.642	1.120	1.739	87	94	92	-54	-29		* 1.92E-6		* 4.24E-6	.
**Renal Cancer**																	
786-0	2.430	5.978	6.298	6.722	6.488	0.205	0.255	109	121	116	-92	-90		* 2.08E-6		* 3.62E-6	* 6.31E-6
A498	3.091	14.250	14.635	15.149	11.772	2.898	2.917	103	108	78	-6	-6		* 2.14E-6		* 8.43E-6	> 1.00E-4
ACHN	1.436	5.807	5.849	6.040	5.700	3.032	2.392	101	105	98	36	22		* 6.01E-6		> 1.00E-4	> 1.00E-4
CAKI-1	0.927	5.749	5.381	5.494	5.781	0.666	1.321	92	95	101	-28	8		* 2.47E-6		.	> 1.00E-4
RXF 393	2.153	.	.	.	.	.	.	.	.	.	.	.		.		.	.
SN12C	1.356	3.860	3.747	3.983	3.665	0.398	0.757	95	105	92	-71	-44		* 1.82E-6		* 3.69E-6	.
TK-10	2.146	7.289	7.782	7.808	7.698	4.385	3.730	110	110	108	44	31		* 7.95E-6		> 1.00E-4	> 1.00E-4
UO-31	1.458	.	.	.	.	.	.	.	.	.	.	.		.		.	.
**Prostate Cancer**																	
PC-3	5.569	.	.	.	.	.	.	.	.	.	.	.		.		.	.
DU-145	1.963	4.648	4.857	4.833	4.962	0.972	1.388	108	107	112	-50	-29		* 2.40E-6		* 4.88E-6	.
**Breast Cancer**																	
MCF7	2.853	14.614	14.811	15.014	14.387	9.168	5.741	102	103	98	54	25		* 1.34E-5		> 1.00E-4	> 1.00E-4
MDA-MB-231/ATCC	4.387	15.617	15.124	15.564	16.023	2.091	1.464	96	100	104	-52	-67		* 2.21E-6		* 4.62E-6	* 9.66E-6
HS 578T	1.737	3.359	3.073	3.111	2.649	0.763	0.637	82	84	56	-56	-63		* 1.13E-6		* 3.16E-6	* 8.82E-6
BT-549	8.085	.	.	.	.	.	.	.	.	.	.	.		.		.	.
T-47D	5.448	10.078	10.253	9.812	10.030	10.675	5.837	104	94	99	113	8		* 4.00E-5		> 1.00E-4	> 1.00E-4
MDA-MB-468	4.562	7.434	7.347	7.231	7.420	7.439	5.633	97	92	100	100	38		* 6.33E-5		> 1.00E-4	> 1.00E-4

Figure S37. NCI's DTP dose-response report for compound **SLT-3**. GI_{50}, TGI and LC_{50} values (expressed as molarity).

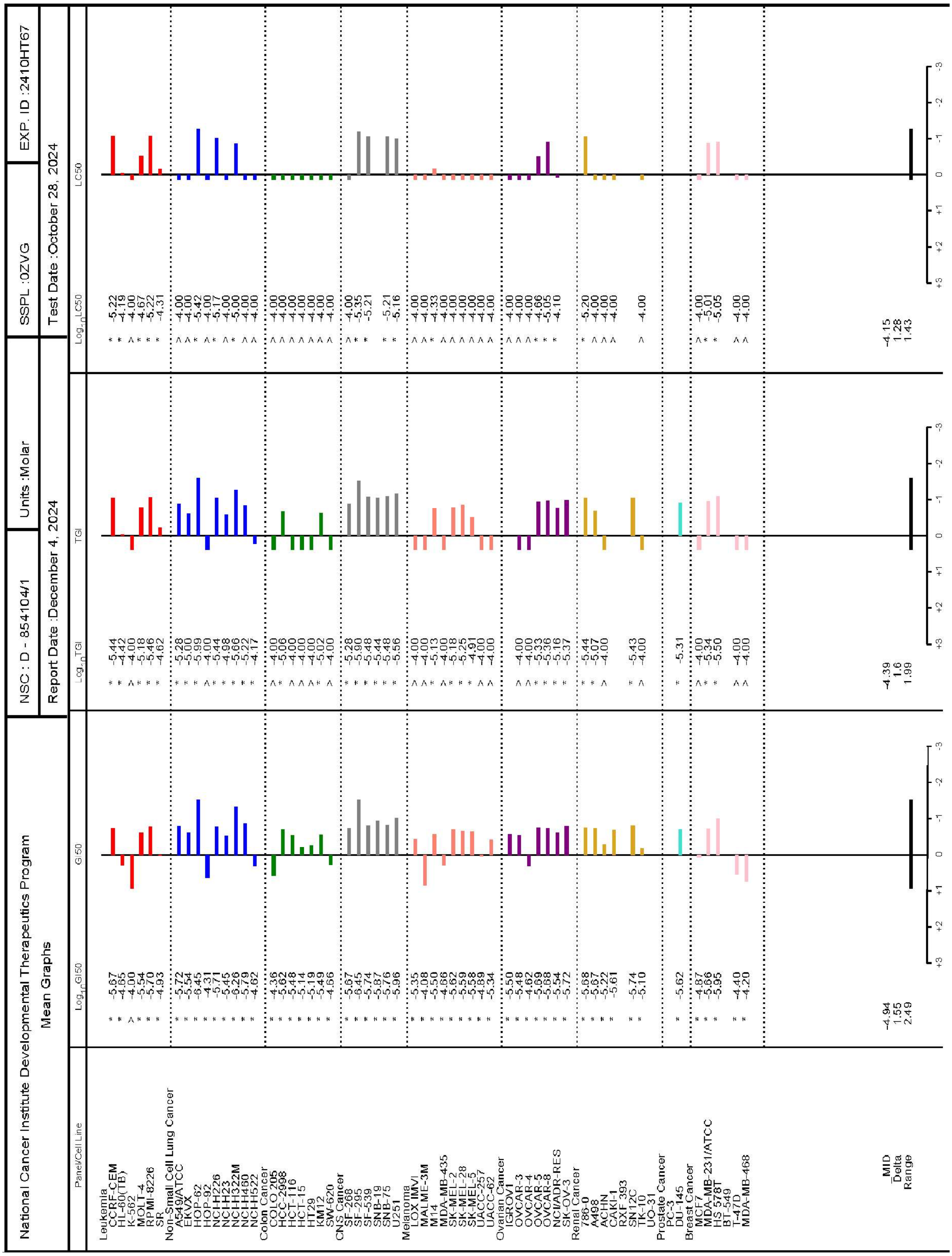

Figure S38. NCI's DTP dose-response report for compound **SLT-3**. Mean graphs of GI_{50}, TGI and LC_{50} values.

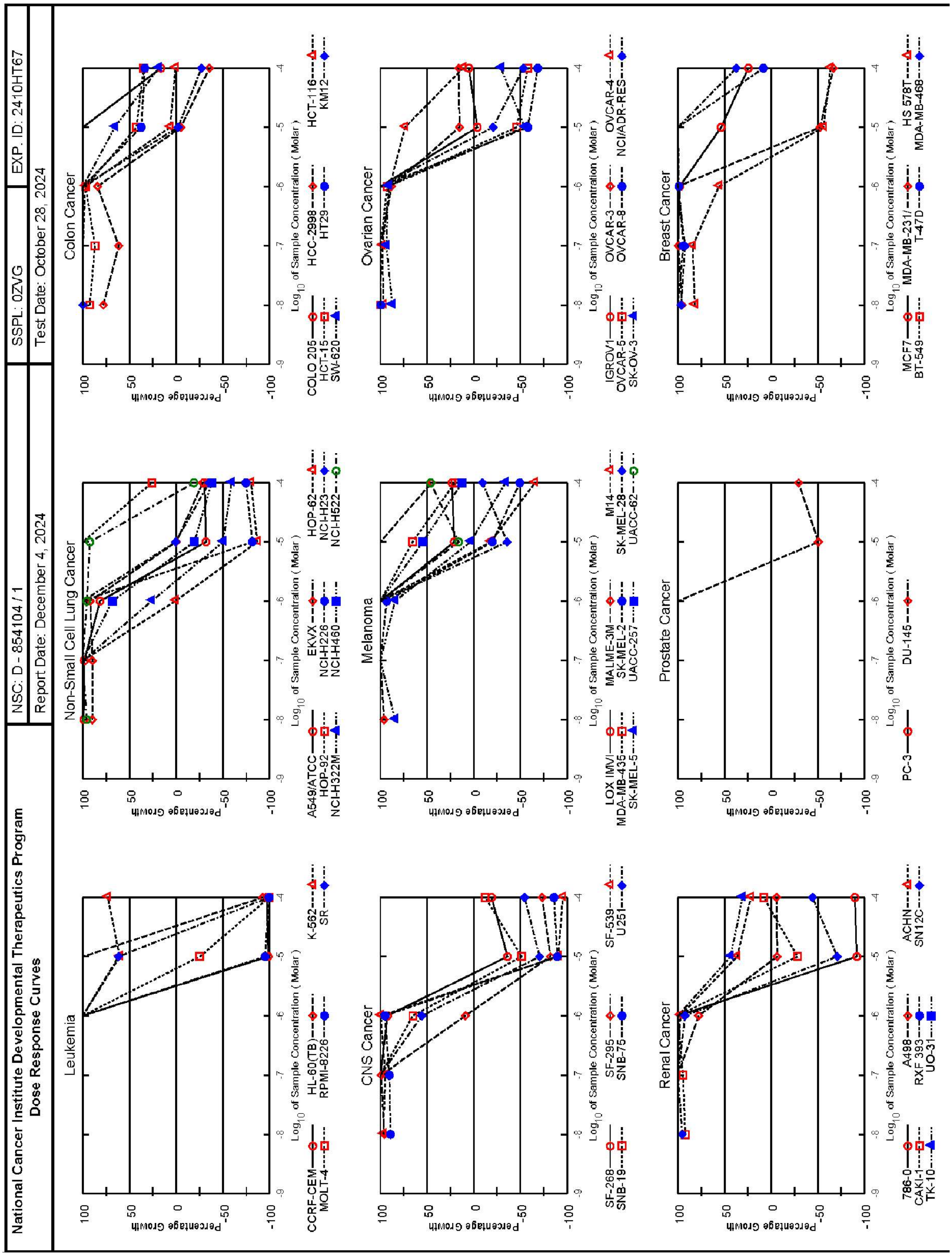

Figure S39. Dose-response curves for **SLT-3.**

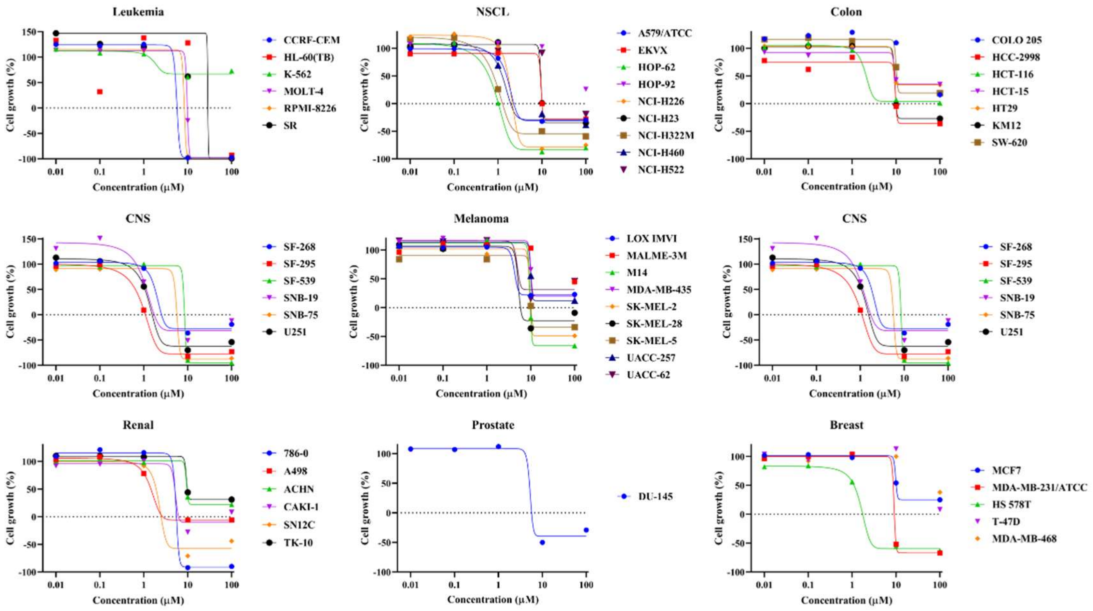

Figure S40. Dose-response curves of **SLT-3** in all the cell lines present in the NCI panel.

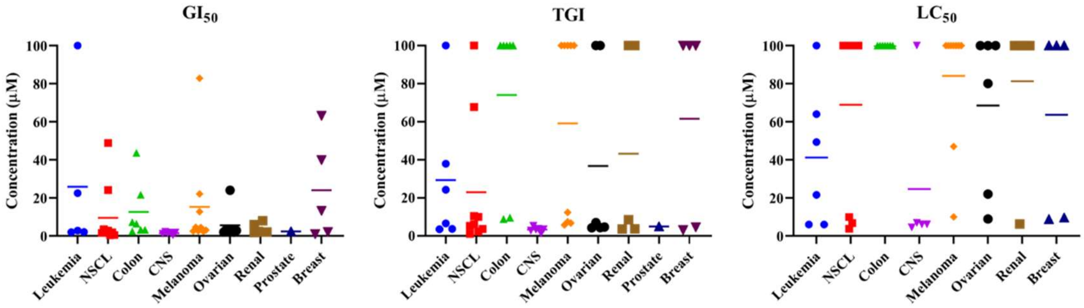

Figure S41. Mean graphs of the GI_{50}, TGI, and LC_{50} values of **SLT-3.**

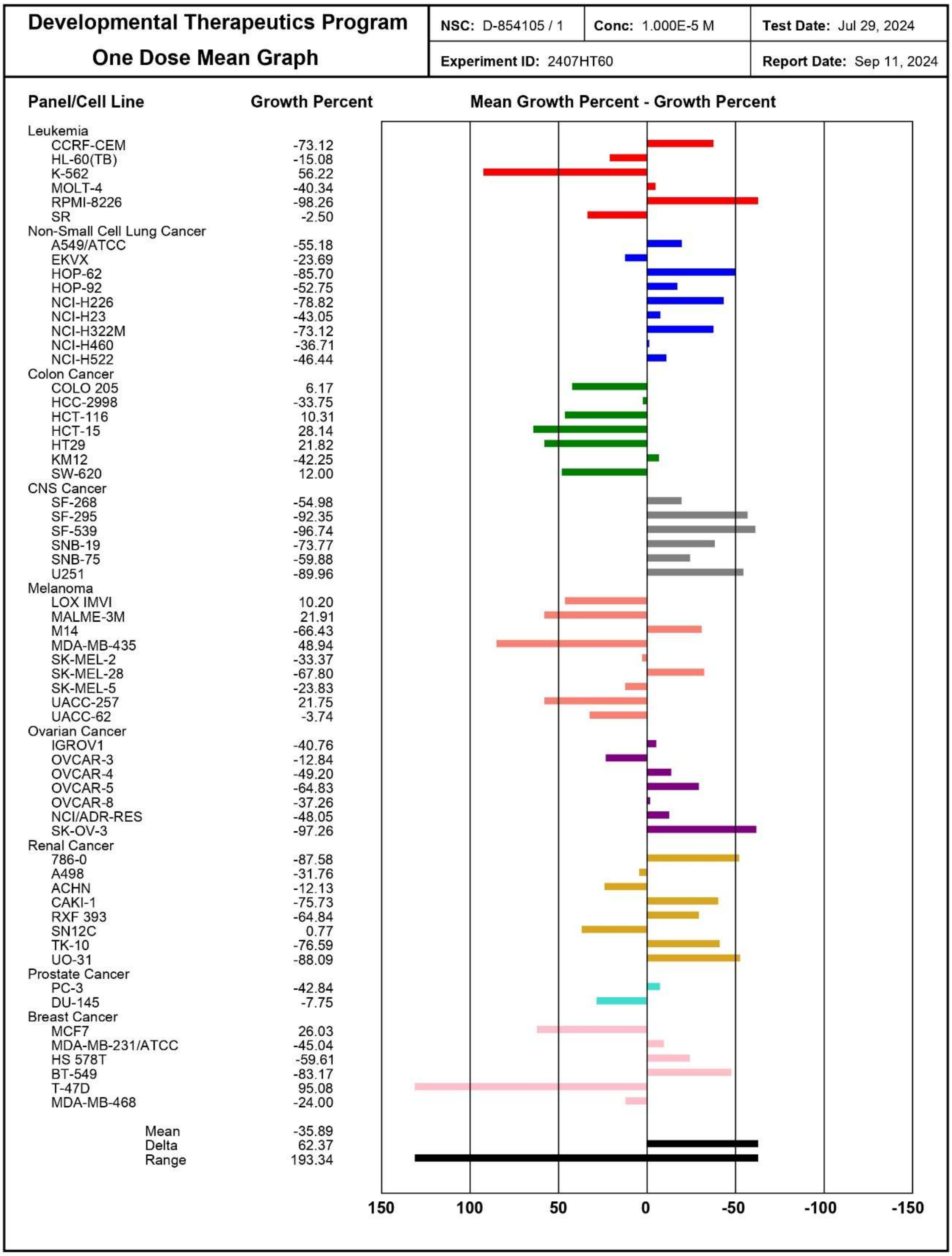

Panel/Cell Line	Growth Percent
Leukemia	
CCRF-CEM	-73.12
HL-60(TB)	-15.08
K-562	56.22
MOLT-4	-40.34
RPMI-8226	-98.26
SR	-2.50
Non-Small Cell Lung Cancer	
A549/ATCC	-55.18
EKVX	-23.69
HOP-62	-85.70
HOP-92	-52.75
NCI-H226	-78.82
NCI-H23	-43.05
NCI-H322M	-73.12
NCI-H460	-36.71
NCI-H522	-46.44
Colon Cancer	
COLO 205	6.17
HCC-2998	-33.75
HCT-116	10.31
HCT-15	28.14
HT29	21.82
KM12	-42.25
SW-620	12.00
CNS Cancer	
SF-268	-54.98
SF-295	-92.35
SF-539	-96.74
SNB-19	-73.77
SNB-75	-59.88
U251	-89.96
Melanoma	
LOX IMVI	10.20
MALME-3M	21.91
M14	-66.43
MDA-MB-435	48.94
SK-MEL-2	-33.37
SK-MEL-28	-67.80
SK-MEL-5	-23.83
UACC-257	21.75
UACC-62	-3.74
Ovarian Cancer	
IGROV1	-40.76
OVCAR-3	-12.84
OVCAR-4	-49.20
OVCAR-5	-64.83
OVCAR-8	-37.26
NCI/ADR-RES	-48.05
SK-OV-3	-97.26
Renal Cancer	
786-0	-87.58
A498	-31.76
ACHN	-12.13
CAKI-1	-75.73
RXF 393	-64.84
SN12C	0.77
TK-10	-76.59
UO-31	-88.09
Prostate Cancer	
PC-3	-42.84
DU-145	-7.75
Breast Cancer	
MCF7	26.03
MDA-MB-231/ATCC	-45.04
HS 578T	-59.61
BT-549	-83.17
T-47D	95.08
MDA-MB-468	-24.00
Mean	-35.89
Delta	62.37
Range	193.34

Figure S32. NCI-60 results at one dose (10 μM) of compound **SLT-4** (NSC 854105) after 48 h of treatment.

National Cancer Institute Developmental Therapeutics Program
In-Vitro Testing Results

NSC : D - 854105 / 1	Experiment ID : 2410HT67	Test Type : HTS	Units : Molar
Report Date : December 4, 2024	Test Date : October 28, 2024	QNS :	MC :
COMI : SLT-7	Stain Reagent :	SSPL : 0ZVG	

Log10 Concentration

	Time		Mean Optical Densities					Percent Growth							
Panel/Cell Line	Zero	Ctrl	-8.0	-7.0	-6.0	-5.0	-4.0	-8.0	-7.0	-6.0	-5.0	-4.0	GI50	TGI	LC50
**Leukemia**															
CCRF-CEM	2.383	8.526	9.760	10.035	9.962	0.061	0.051	120	125	123	-97	-98	* 2.15E-6	* 3.62E-6	* 6.10E-6
HL-60(TB)	0.762	3.539	4.350	4.450	4.355	0.759	0.013	129	133	129	-10	-98	* 3.70E-6	* 8.42E-6	* 2.82E-5
K-562	0.572	7.827	8.935	8.777	8.252	3.724	6.131	115	113	106	44	77	.	> 1.00E-4	> 1.00E-4
MOLT-4	1.004	4.344	4.828	4.588	4.696	0.210	0.010	114	107	111	-79	-99	* 2.09E-6	* 3.83E-6	* 7.02E-6
RPMI-8226	6.912	19.179	21.139	21.218	21.291	0.081	0.040	116	117	117	-99	-99	* 2.05E-6	* 3.49E-6	* 5.94E-6
SR	0.173	0.904	1.118	1.467	0.947	0.008	0.002	129	177	106	-95	-99	* 1.90E-6	* 3.36E-6	* 5.96E-6
**Non-Small Cell Lung Cancer**															
A549/ATCC	0.672	5.520	5.490	5.262	1.789	0.272	0.524	99	95	23	-60	-22	* 4.20E-7	* 1.90E-6	.
EKVX	5.546	11.035	10.506	10.533	10.213	3.073	3.833	90	91	85	-45	-31	* 1.86E-6	* 4.53E-6	> 1.00E-4
HOP-62	1.310	3.447	3.511	3.151	1.220	0.208	0.159	103	86	-7	-84	-88	* 2.45E-7	* 8.44E-7	* 3.61E-6
HOP-92	5.399	13.855	13.973	13.456	14.152	3.690	7.292	101	95	104	-32	22	* 2.49E-6	.	> 1.00E-4
NCI-H226	9.434	12.254	12.317	12.560	9.930	1.407	2.696	103	111	18	-85	-71	* 4.52E-7	* 1.49E-6	* 4.57E-6
NCI-H23	3.807	13.460	13.994	13.735	14.339	2.586	2.402	106	103	109	-32	-37	* 2.62E-6	* 5.92E-6	> 1.00E-4
NCI-H322M	3.247	8.307	8.709	9.009	2.799	0.953	0.630	108	114	-14	-71	-81	* 3.16E-7	* 7.80E-7	* 4.34E-6
NCI-H460	1.152	16.510	17.835	16.459	5.631	0.690	0.544	109	100	29	-40	-53	* 5.07E-7	* 2.64E-6	* 6.03E-5
NCI-H522	5.459	11.960	11.542	11.894	11.285	4.109	4.623	94	99	90	-25	-15	* 2.22E-6	* 6.08E-6	> 1.00E-4
**Colon Cancer**															
COLO 205	0.651	2.554	2.855	3.032	2.933	1.653	0.675	116	125	120	53	-1	* 1.12E-5	* 9.51E-5	> 1.00E-4
HCC-2998	4.637	14.982	12.908	11.534	12.260	4.796	3.201	80	67	74	2	-31	* 2.13E-6	* 1.11E-5	> 1.00E-4
HCT-116	0.718	4.642	4.803	4.844	3.152	0.667	0.540	104	105	62	-7	-25	* 1.49E-6	* 7.90E-6	> 1.00E-4
HCT-15	2.224	20.033	18.343	18.700	20.012	7.699	7.286	90	93	100	31	28	* 5.30E-6	> 1.00E-4	> 1.00E-4
HT29	0.858	6.729	6.429	6.740	6.359	2.490	2.581	95	100	94	28	29	* 4.60E-6	> 1.00E-4	> 1.00E-4
KM12	0.515	2.623	2.678	2.788	2.138	0.272	0.234	103	108	77	-47	-54	* 1.65E-6	* 4.16E-6	* 2.41E-5
SW-620	0.447	2.511	2.723	2.756	2.872	0.975	0.370	110	112	118	26	-17	* 5.42E-6	* 3.95E-5	> 1.00E-4
**CNS Cancer**															
SF-268	1.150	3.512	3.161	3.177	3.260	0.323	0.703	85	86	89	-72	-39	* 1.76E-6	* 3.58E-6	.
SF-295	2.699	6.825	6.780	6.718	2.361	0.226	0.335	99	98	-12	-92	-88	* 2.70E-7	* 7.69E-7	* 2.98E-6
SF-539	2.612	9.868	9.808	9.221	7.385	0.103	0.058	99	91	66	-96	-98	* 1.25E-6	* 2.55E-6	* 5.19E-6
SNB-19	0.764	1.537	1.783	1.673	1.096	0.137	0.545	132	118	43	-82	-29	* 8.04E-7	* 2.21E-6	.
SNB-75	2.121	4.196	3.790	4.035	3.522	0.038	0.816	80	92	67	-98	-62	* 1.27E-6	* 2.55E-6	* 5.12E-6
U251	0.840	4.125	4.583	4.383	1.524	0.084	0.436	114	108	21	-90	-48	* 4.62E-7	* 1.54E-6	.
**Melanoma**															
LOX IMVI	0.569	3.939	4.290	4.439	4.174	1.154	1.719	110	115	107	17	34	* 4.32E-6	> 1.00E-4	> 1.00E-4
MALME-3M	7.908	14.227	14.023	14.540	14.123	14.678	11.452	97	105	99	108	56	> 1.00E-4	> 1.00E-4	> 1.00E-4
M14	3.018	11.834	12.623	12.792	12.332	1.549	0.892	109	111	106	-49	-70	* 2.29E-6	* 4.84E-6	* 1.15E-5
MDA-MB-435	1.295	4.207	4.307	4.522	4.518	1.999	1.800	103	111	111	24	17	* 5.03E-6	> 1.00E-4	> 1.00E-4
SK-MEL-2	1.837	4.972	4.601	4.881	4.754	0.816	0.652	88	97	93	-56	-64	* 1.95E-6	* 4.23E-6	* 9.17E-6
SK-MEL-28	3.945	7.060	7.550	7.071	7.214	1.046	4.776	116	100	105	-74	27	* 2.03E-6	.	.
SK-MEL-5	3.148	12.690	11.782	12.018	12.015	1.941	2.078	91	93	93	-38	-34	* 2.13E-6	* 5.11E-6	> 1.00E-4
UACC-257	3.162	6.608	6.736	7.265	7.126	4.679	3.541	104	119	115	44	11	* 8.23E-6	> 1.00E-4	> 1.00E-4
UACC-62	0.869	3.284	3.482	3.628	3.710	0.768	2.118	108	115	118	-12	52	.	.	> 1.00E-4
**Ovarian Cancer**															
IGROV1	1.064	4.205	4.180	4.413	4.107	0.613	1.092	100	107	97	-42	1	* 2.18E-6	.	> 1.00E-4
OVCAR-3	3.350	10.365	11.216	9.944	9.954	2.787	2.949	112	94	94	-17	-12	* 2.51E-6	* 7.06E-6	> 1.00E-4
OVCAR-4	4.819	9.106	8.192	8.465	8.385	8.083	4.332	78	84	84	77	-10	* 2.03E-5	* 7.65E-5	> 1.00E-4
OVCAR-5	4.584	14.398	14.411	14.474	3.994	1.498	1.574	100	101	-13	-67	-66	* 2.80E-7	* 7.70E-7	* 4.80E-6
OVCAR-8	0.817	4.767	4.623	4.791	4.270	0.145	0.255	96	101	87	-82	-69	* 1.66E-6	* 3.28E-6	* 6.46E-6
NCI/ADR-RES	3.596	12.384	12.955	13.215	13.404	1.700	1.343	107	109	112	-53	-63	* 2.37E-6	* 4.78E-6	* 9.63E-6
SK-OV-3	2.453	7.029	6.917	7.085	5.185	0.510	1.725	98	101	60	-79	-30	* 1.18E-6	* 2.69E-6	.
**Renal Cancer**															
786-0	2.430	5.978	6.281	5.219	6.137	0.168	0.226	108	82	105	-93	-91	* 1.90E-6	* 3.39E-6	* 6.06E-6
A498	3.091	14.250	14.775	14.696	7.000	2.521	2.740	105	104	35	-18	-11	* 6.07E-7	* 4.52E-6	> 1.00E-4
ACHN	1.436	5.807	5.911	5.954	5.777	2.594	1.476	102	103	99	26	1	* 4.76E-6	> 1.00E-4	> 1.00E-4
CAKI-1	0.927	5.749	5.180	5.477	5.833	0.343	1.092	88	94	102	-63	3	* 2.06E-6	.	.
RXF 393	2.153	.	.	.	.	.	.	.	.	.	.	.	.	.	.
SN12C	1.356	3.860	3.705	3.783	3.540	0.219	0.479	94	97	87	-84	-65	* 1.65E-6	* 3.24E-6	* 6.34E-6
TK-10	2.146	7.289	7.481	7.501	7.473	3.351	3.257	104	104	104	23	22	* 4.66E-6	> 1.00E-4	> 1.00E-4
UO-31	1.458	.	.	.	.	.	.	.	.	.	.	.	.	.	.
**Prostate Cancer**															
PC-3	5.569	.	.	.	.	.	.	.	.	.	.	.	.	.	.
DU-145	1.963	4.648	4.875	4.833	5.219	0.177	1.295	108	107	121	-91	-34	* 2.17E-6	* 3.73E-6	.
**Breast Cancer**															
MCF7	2.853	14.614	13.334	15.084	14.318	5.969	6.786	89	104	98	26	33	* 4.67E-6	> 1.00E-4	> 1.00E-4
MDA-MB-231/ATCC	4.387	15.617	15.744	16.331	15.851	1.391	1.501	101	106	102	-68	-66	* 2.02E-6	* 3.97E-6	* 7.81E-6
HS 578T	1.737	3.359	3.034	2.757	1.923	0.302	0.501	80	63	11	-83	-71	* 1.77E-7	* 1.32E-6	* 4.50E-6
BT-549	8.085	.	.	.	.	.	.	.	.	.	.	.	.	.	.
T-47D	5.448	10.078	9.737	10.054	9.849	10.317	4.793	93	100	95	105	-12	* 2.96E-5	* 7.90E-5	> 1.00E-4
MDA-MB-468	4.562	7.434	7.622	7.217	7.409	7.356	3.288	107	93	100	98	-28	* 2.39E-5	* 5.99E-5	> 1.00E-4

Figure S43. NCI's DTP dose-response report for compound **SLT-4**. GI_{50}, TGI and LC_{50} values (expressed as molarity).

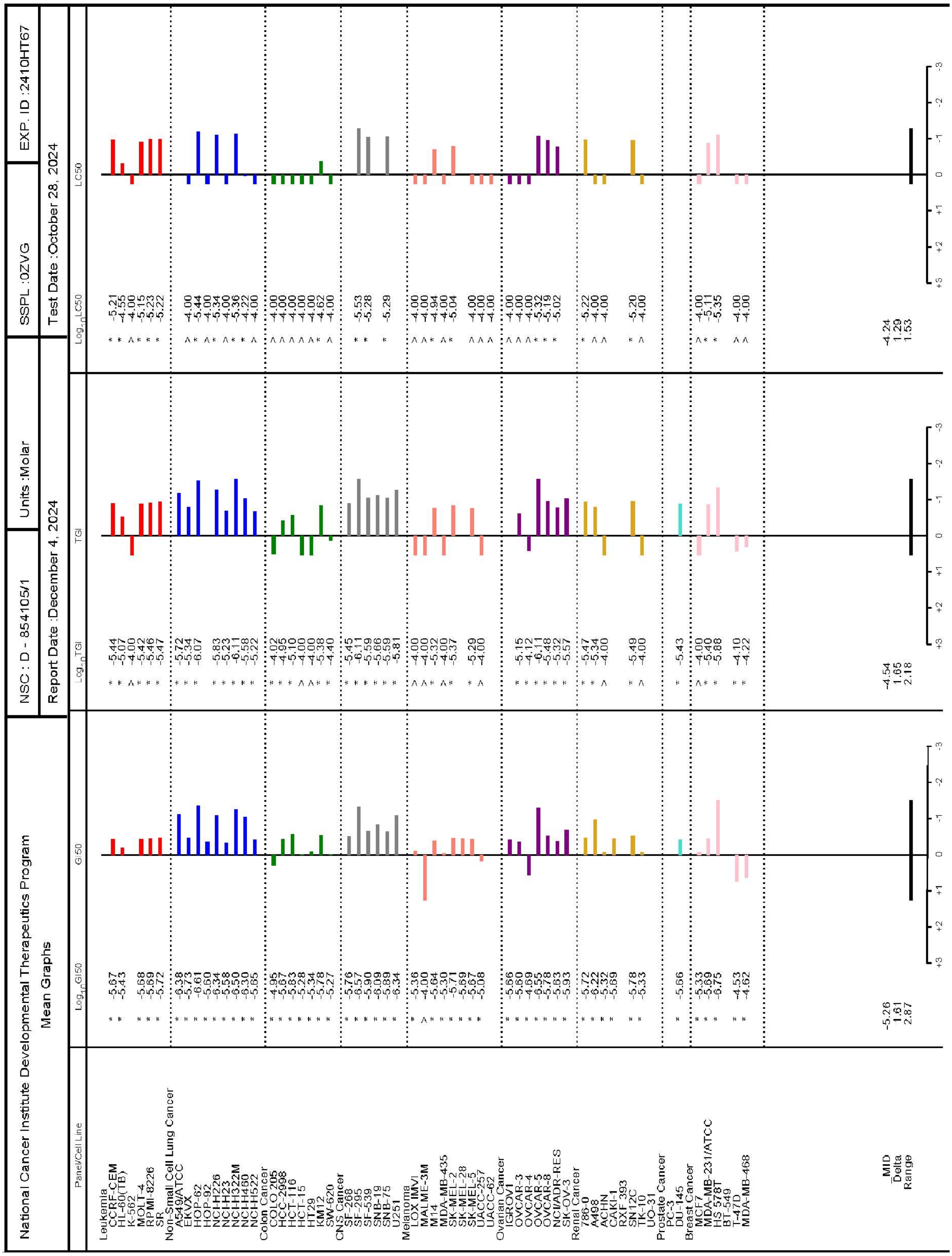

Figure S44. NCI's DTP dose-response report for compound **SLT-4**. Mean graphs of GI_{50}, TGI and LC_{50} values.

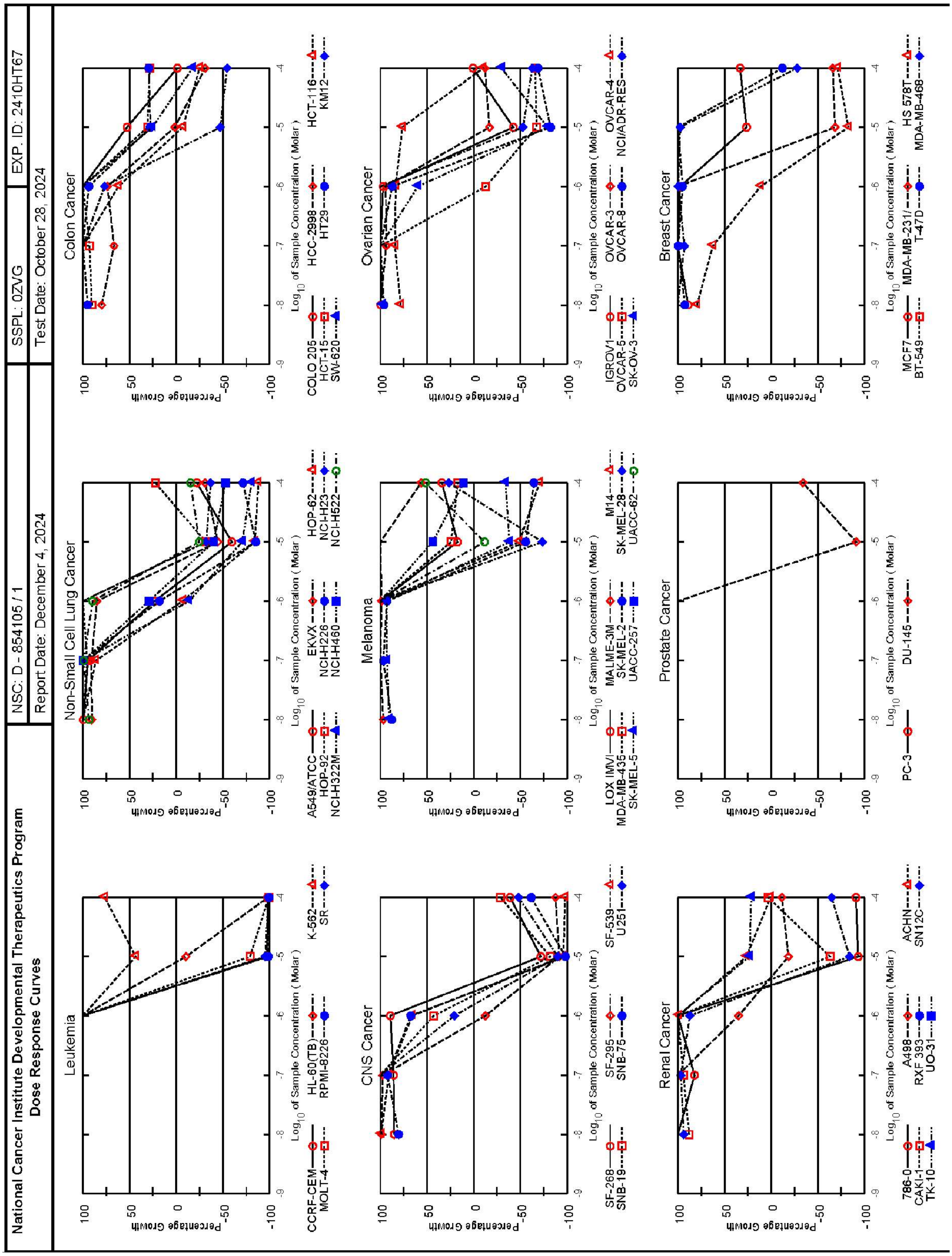

Figure S45. Dose-response curves for **SLT-4.**

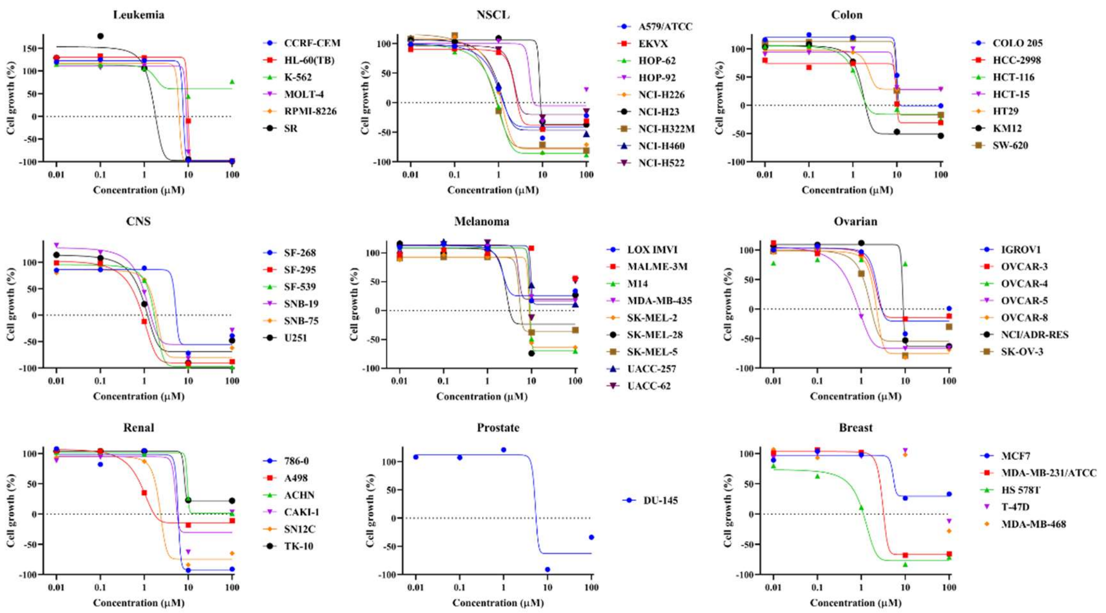

Figure S46. Dose-response curves of **SLT-4** in all the cell lines present in the NCI panel.

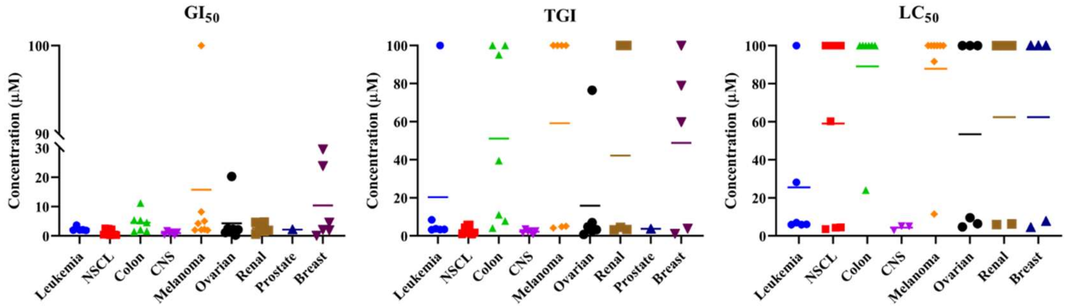

Figure S47. Mean graphs of the GI_{50}, TGI, and LC_{50} values of **SLT-4.**

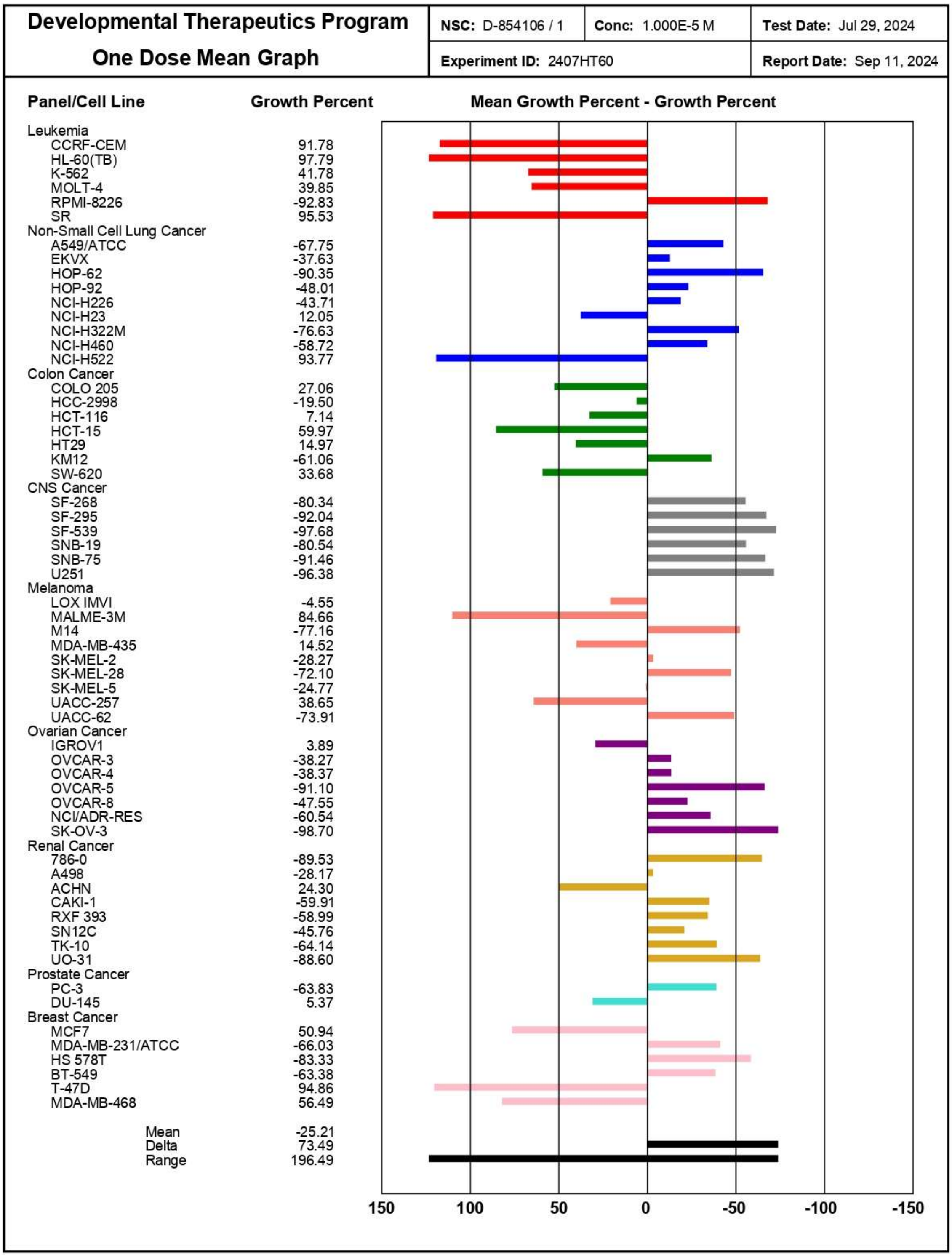

Figure S48. NCI-60 results at one dose (10 μM) of compound **SLT-5** (NSC 854106) after 48 h of treatment.

National Cancer Institute Developmental Therapeutics Program
In-Vitro Testing Results

NSC : D - 854106 / 1	Experiment ID : 2410HT67	Test Type : HTS	Units : Molar
Report Date : December 4, 2024	Test Date : October 28, 2024	QNS :	MC :
COMI : SLT-8	Stain Reagent :	SSPL : 0ZVG	

Log10 Concentration

	Time		Mean Optical Densities					Percent Growth							
Panel/Cell Line	Zero	Ctrl	-8.0	-7.0	-6.0	-5.0	-4.0	-8.0	-7.0	-6.0	-5.0	-4.0	GI50	TGI	LC50
**Leukemia**															
CCRF-CEM	2.383	8.526	9.735	9.854	9.845	0.313	0.030	120	122	121	-87	-99	* 2.20E-6	* 3.83E-6	* 6.65E-6
HL-60(TB)	0.762	3.539	4.386	4.247	4.371	4.290	0.117	131	126	130	127	-85	* 2.31E-5	* 3.99E-5	* 6.86E-5
K-562	0.572	7.827	9.110	9.076	8.511	5.563	2.718	118	117	109	69	30	* 3.02E-5	> 1.00E-4	> 1.00E-4
MOLT-4	1.004	4.344	4.552	4.606	4.679	4.698	0.020	106	108	110	111	-98	* 1.95E-5	* 3.39E-5	* 5.88E-5
RPMI-8226	6.912	19.179	20.359	21.209	21.533	1.467	0.046	110	117	119	-79	-99	* 2.24E-6	* 4.00E-6	* 7.16E-6
SR	0.173	0.904	1.056	1.125	1.030	1.063	0.003	121	130	117	121	-98	* 2.11E-5	* 3.57E-5	* 6.03E-5
**Non-Small Cell Lung Cancer**															
A549/ATCC	0.672	5.520	5.387	5.475	5.549	0.379	0.121	97	99	101	-44	-82	* 2.24E-6	* 4.98E-6	* 1.47E-5
EKVX	5.546	11.035	10.249	10.498	10.234	10.675	1.368	86	90	85	93	-75	* 1.81E-5	* 3.58E-5	* 7.08E-5
HOP-62	1.310	3.447	3.549	3.592	2.004	0.128	0.166	105	107	32	-90	-87	* 5.81E-7	* 1.84E-6	* 4.70E-6
HOP-92	5.399	13.855	14.054	13.915	13.734	13.960	7.703	102	101	99	101	27	* 4.93E-5	> 1.00E-4	> 1.00E-4
NCI-H226	9.434	12.254	12.217	12.529	12.568	1.450	0.766	99	110	111	-85	-92	* 2.06E-6	* 3.70E-6	* 6.66E-6
NCI-H23	3.807	13.460	14.000	14.317	14.480	7.345	1.410	106	109	110	37	-63	* 6.58E-6	* 2.33E-5	* 7.41E-5
NCI-H322M	3.247	8.307	8.326	8.637	8.640	1.604	0.341	100	106	107	-51	-90	* 2.29E-6	* 4.76E-6	* 9.91E-6
NCI-H460	1.152	16.510	15.836	16.211	15.777	1.267	0.314	96	98	95	1	-73	* 3.01E-6	* 1.02E-5	* 4.90E-5
NCI-H522	5.459	11.960	11.218	11.343	11.774	11.237	2.215	89	90	97	89	-59	* 1.83E-5	* 3.97E-5	* 8.64E-5
**Colon Cancer**															
COLO 205	0.651	2.554	2.789	2.857	3.016	2.978	0.863	113	116	124	122	11	* 4.48E-5	> 1.00E-4	> 1.00E-4
HCC-2998	4.637	14.982	11.628	16.574	11.935	4.330	0.888	68	115	70	-7	-81	* 1.85E-6	* 8.20E-6	* 3.84E-5
HCT-116	0.718	4.642	4.706	4.701	4.877	1.338	0.293	102	101	106	16	-59	* 4.17E-6	* 1.62E-5	* 7.53E-5
HCT-15	2.224	20.033	18.233	19.803	19.126	11.786	4.778	90	99	94	54	14	* 1.26E-5	> 1.00E-4	> 1.00E-4
HT29	0.858	6.729	6.577	6.748	6.799	2.080	1.159	97	100	101	21	5	* 4.34E-6	> 1.00E-4	> 1.00E-4
KM12	0.515	2.623	2.670	2.620	2.660	0.469	0.085	102	100	102	-9	-84	* 2.94E-6	* 8.31E-6	* 3.56E-5
SW-620	0.447	2.511	2.811	2.640	2.805	2.856	0.503	115	106	114	117	3	* 3.85E-5	> 1.00E-4	> 1.00E-4
**CNS Cancer**															
SF-268	1.150	3.512	3.306	3.171	3.502	0.521	0.067	91	86	100	-55	-94	* 2.10E-6	* 4.42E-6	* 9.33E-6
SF-295	2.699	6.825	6.746	6.716	6.142	0.383	0.367	98	97	84	-86	-86	* 1.58E-6	* 3.11E-6	* 6.15E-6
SF-539	2.612	9.868	9.580	9.430	9.410	0.670	0.025	96	94	94	-74	-99	* 1.82E-6	* 3.61E-6	* 7.16E-6
SNB-19	0.764	1.537	1.658	1.698	1.383	0.343	0.050	116	121	80	-55	-94	* 1.67E-6	* 3.91E-6	* 9.16E-6
SNB-75	2.121	4.196	3.827	3.905	4.038	0.127	0.017	82	86	92	-94	-99	* 1.69E-6	* 3.13E-6	* 5.81E-6
U251	0.840	4.125	4.419	4.278	4.062	0.222	0.016	109	105	98	-74	-98	* 1.91E-6	* 3.73E-6	* 7.28E-6
**Melanoma**															
LOX IMVI	0.569	3.939	4.008	4.332	4.096	1.165	0.474	102	112	105	18	-17	* 4.25E-6	* 3.27E-5	> 1.00E-4
MALME-3M	7.908	14.227	13.855	14.028	14.161	14.168	10.918	94	97	99	99	48	* 9.12E-5	> 1.00E-4	> 1.00E-4
M14	3.018	11.834	12.362	13.243	13.007	3.289	0.878	106	116	113	3	-71	* 3.75E-6	* 1.10E-5	* 5.22E-5
MDA-MB-435	1.295	4.207	4.327	4.842	4.594	2.571	1.463	104	122	113	44	6	* 8.15E-6	> 1.00E-4	> 1.00E-4
SK-MEL-2	1.837	4.972	4.805	4.706	5.200	2.874	0.566	94	91	107	33	-69	* 5.88E-6	* 2.10E-5	* 6.49E-5
SK-MEL-28	3.945	7.060	7.087	7.279	7.188	2.331	0.524	101	107	104	-41	-87	* 2.36E-6	* 5.22E-6	* 1.58E-5
SK-MEL-5	3.148	12.690	12.109	11.004	12.103	2.568	0.868	94	82	94	-18	-72	* 2.46E-6	* 6.86E-6	* 3.84E-5
UACC-257	3.162	6.608	7.044	6.938	7.221	6.634	3.428	113	109	118	101	8	* 3.51E-5	> 1.00E-4	> 1.00E-4
UACC-62	0.869	3.284	3.521	3.665	3.384	0.757	0.324	110	116	105	-16	-63	* 2.84E-6	* 7.37E-6	* 5.34E-5
**Ovarian Cancer**															
IGROV1	1.064	4.205	4.273	4.277	4.444	1.230	0.224	102	102	108	5	-79	* 3.67E-6	* 1.16E-5	* 4.54E-5
OVCAR-3	3.350	10.365	10.680	10.840	10.253	7.564	1.265	104	107	98	60	-62	* 1.21E-5	* 3.10E-5	* 7.94E-5
OVCAR-4	4.819	9.106	8.223	8.749	8.092	8.157	1.820	80	92	76	77	-62	* 1.57E-5	* 3.58E-5	* 8.17E-5
OVCAR-5	4.584	14.398	14.359	14.353	14.436	2.489	0.757	100	100	100	-46	-84	* 2.21E-6	* 4.87E-6	* 1.30E-5
OVCAR-8	0.817	4.767	4.641	4.928	4.701	0.767	0.019	97	104	98	-11	-98	* 2.78E-6	* 7.98E-6	* 2.83E-5
NCI/ADR-RES	3.596	12.384	12.355	13.170	13.146	7.720	0.707	100	109	109	47	-80	* 8.92E-6	* 2.34E-5	* 5.78E-5
SK-OV-3	2.453	7.029	7.015	6.521	7.063	0.739	0.111	100	89	101	-70	-96	* 1.98E-6	* 3.90E-6	* 7.65E-6
**Renal Cancer**															
786-0	2.430	5.978	5.274	6.251	6.410	0.168	0.025	87	108	113	-93	-99	* 2.02E-6	* 3.53E-6	* 6.18E-6
A498	3.091	14.250	14.506	15.074	14.495	2.997	1.923	102	107	102	-3	-38	* 3.13E-6	* 9.36E-6	> 1.00E-4
ACHN	1.436	5.807	5.674	5.589	5.718	1.978	1.240	97	95	98	12	-14	* 3.64E-6	* 2.99E-5	> 1.00E-4
CAKI-1	0.927	5.749	5.415	5.565	5.379	1.536	0.177	93	96	92	13	-81	* 3.40E-6	* 1.37E-5	* 4.68E-5
RXF 393	2.153	.	.	.	.	.	.	.	.	.	.	.	.	.	.
SN12C	1.356	3.860	3.261	3.322	3.465	0.750	0.073	76	79	84	-45	-95	* 1.84E-6	* 4.50E-6	* 1.28E-5
TK-10	2.146	7.289	7.267	7.866	7.712	4.611	2.213	100	112	108	48	1	* 9.24E-6	> 1.00E-4	> 1.00E-4
UO-31	1.458	.	.	.	.	.	.	.	.	.	.	.	.	.	.
**Prostate Cancer**															
PC-3	5.569	.	.	.	.	.	.	.	.	.	.	.	.	.	.
DU-145	1.963	4.648	4.824	4.822	4.832	1.247	0.043	107	106	107	-36	-98	* 2.49E-6	* 5.57E-6	* 1.66E-5
**Breast Cancer**															
MCF7	2.853	14.614	14.685	13.953	13.145	11.649	3.391	101	94	88	75	5	* 2.25E-5	> 1.00E-4	> 1.00E-4
MDA-MB-231/ATCC	4.387	15.617	15.454	15.610	15.781	3.859	0.528	98	100	101	-12	-88	* 2.84E-6	* 7.83E-6	* 3.16E-5
HS 578T	1.737	3.359	2.707	2.720	2.601	0.363	0.089	60	61	53	-79	-95	* 1.06E-6	* 2.53E-6	* 6.03E-6
BT-549	8.085	.	.	.	.	.	.	.	.	.	.	.	.	.	.
T-47D	5.448	10.078	9.797	9.778	9.727	9.872	10.013	94	94	92	96	98	> 1.00E-4	> 1.00E-4	> 1.00E-4
MDA-MB-468	4.562	7.434	7.444	7.313	7.126	7.272	6.775	100	96	89	94	78	> 1.00E-4	> 1.00E-4	> 1.00E-4

Figure S49. NCI's DTP dose-response report for compound **SLT-5**. GI_{50}, TGI and LC_{50} values (expressed as molarity).

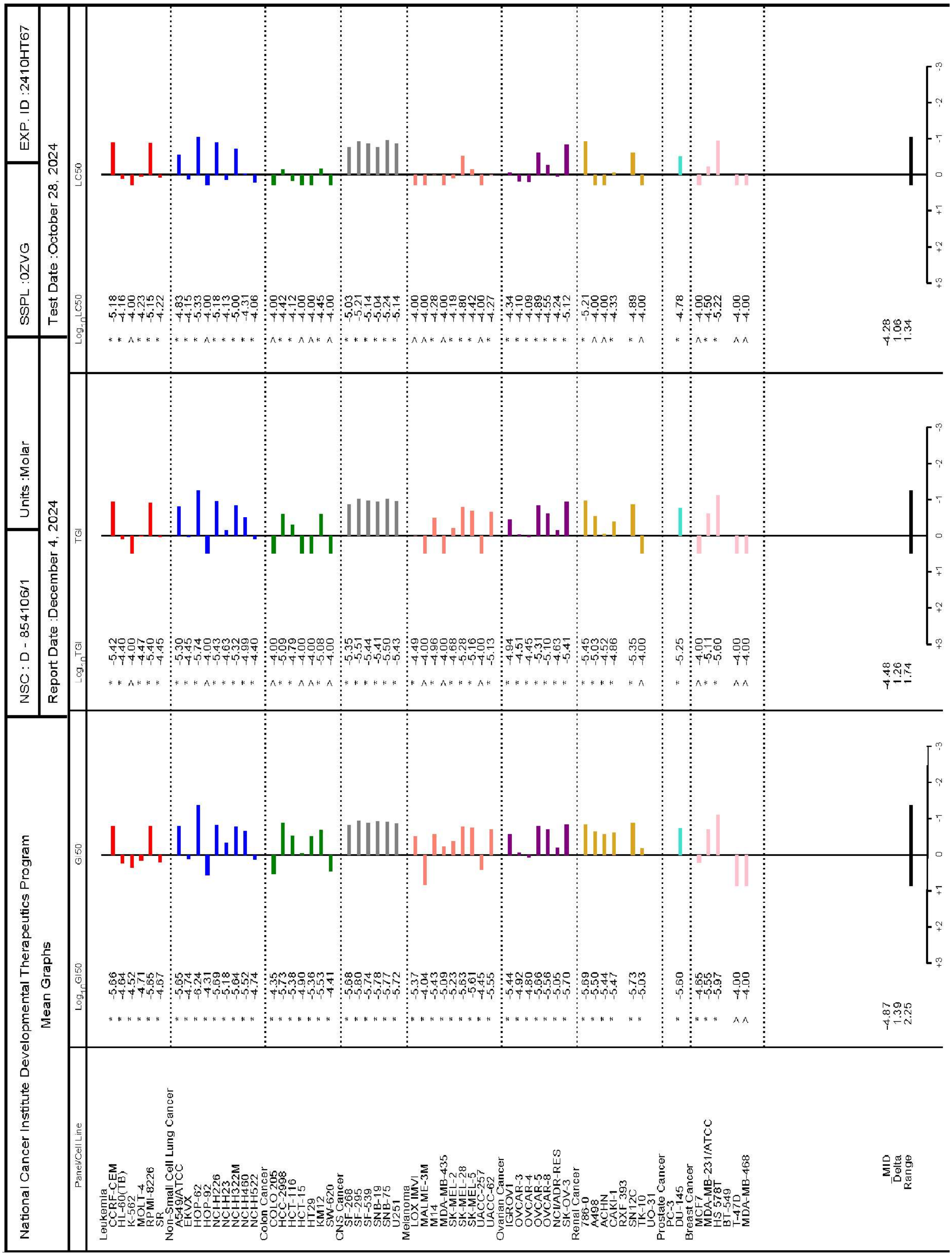

Figure S50. NCI's DTP dose-response report for compound **SLT-5**. Mean graphs of GI_{50}, TGI and LC_{50} values.

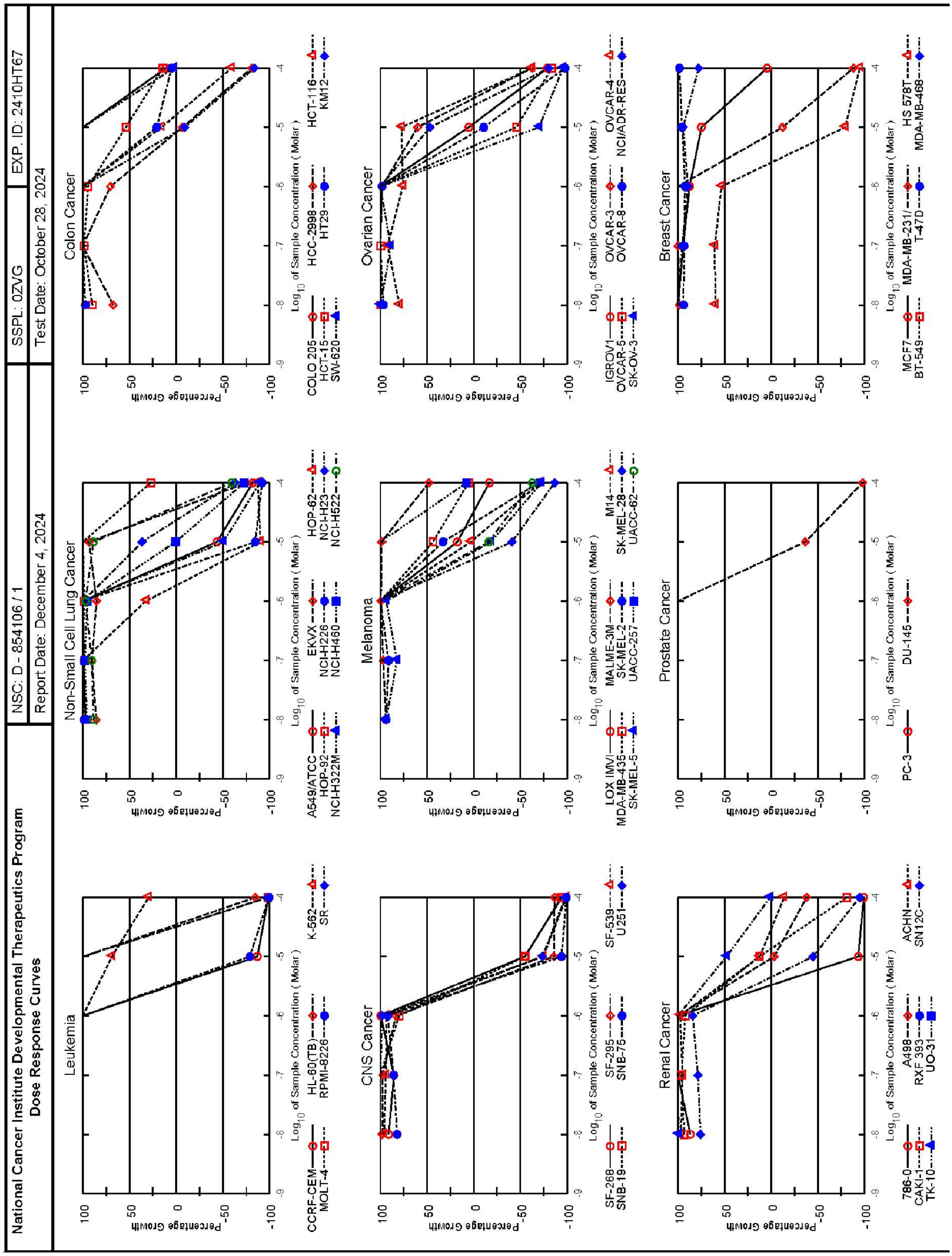

Figure S51. Dose-response curves for **SLT-5.**

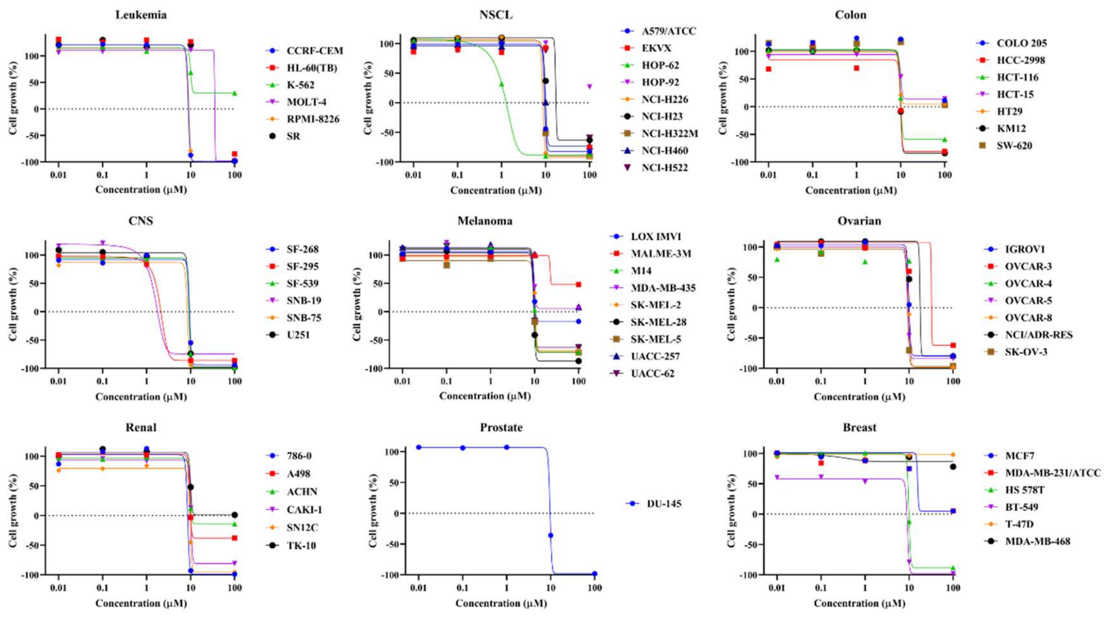

Figure S52. Dose-response curves of **SLT-5** in all the cell lines present in the NCI panel.

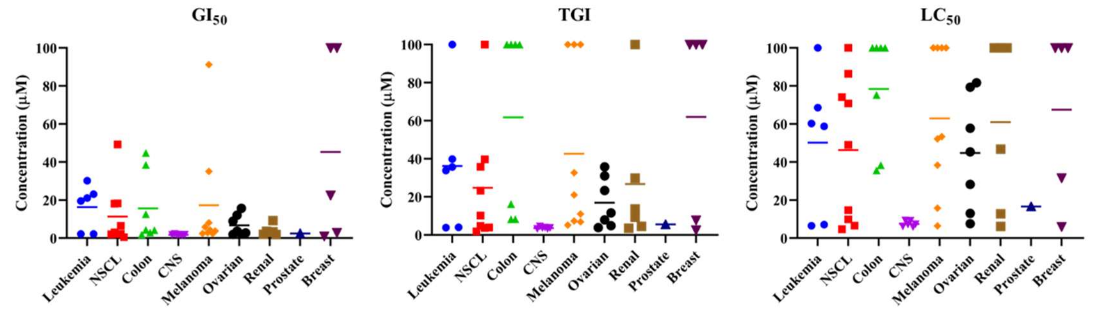

Figure S53. Mean graphs of the GI_{50}, TGI, and LC_{50} values of **SLT-5.**

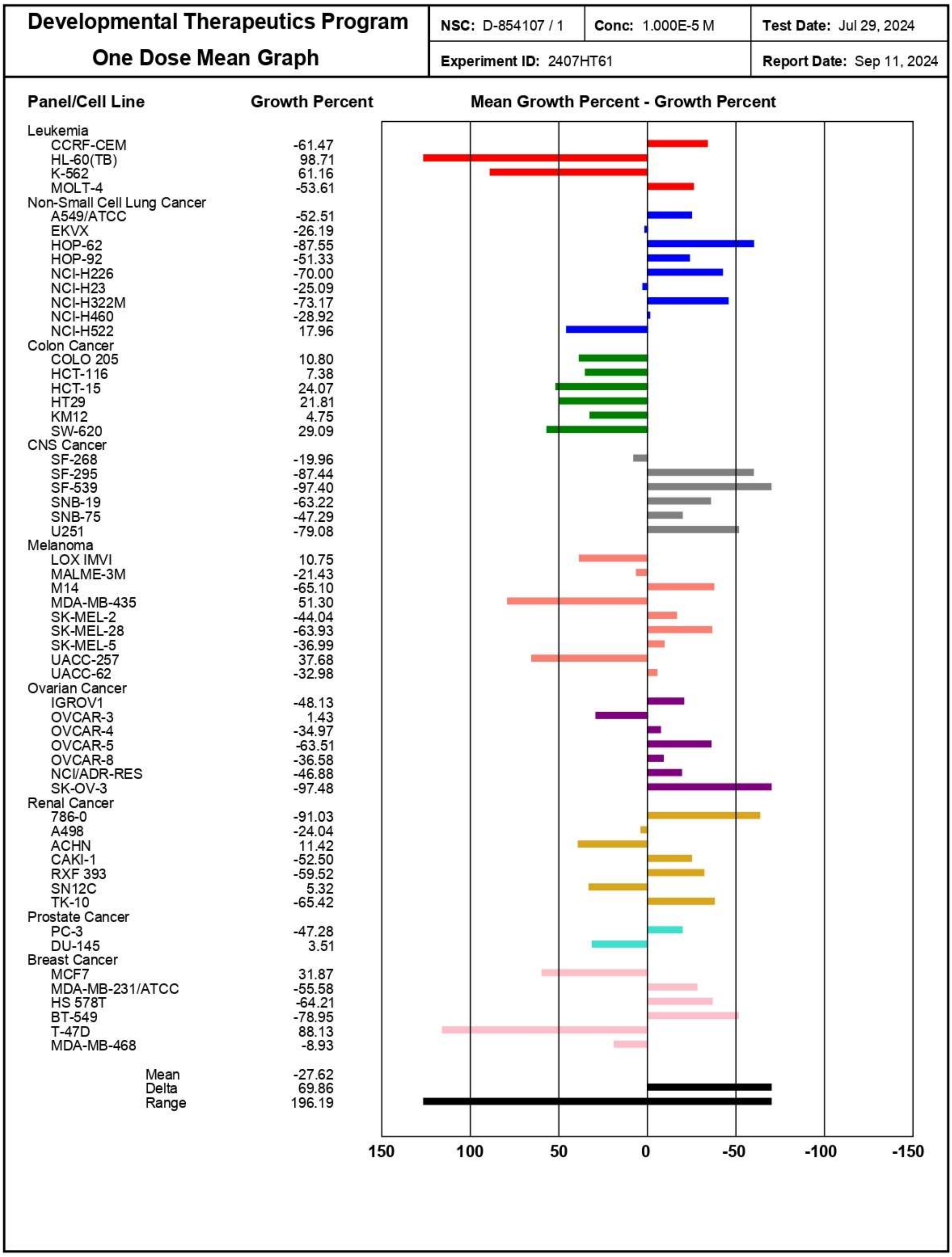

Figure S54. NCI-60 results at one dose (10 μM) of compound **SLT-6** (NSC 854107) after 48 h of treatment.

National Cancer Institute Developmental Therapeutics Program
In-Vitro Testing Results

NSC : D - 854107 / 1	Experiment ID : 2410HT67	Test Type : HTS	Units : Molar
Report Date : December 4, 2024	Test Date : October 28, 2024	QNS :	MC :
COMI : SLT-9	Stain Reagent :	SSPL : 0ZVG	

			Log10 Concentration												
	Time		Mean Optical Densities					Percent Growth							
Panel/Cell Line	Zero	Ctrl	-8.0	-7.0	-6.0	-5.0	-4.0	-8.0	-7.0	-6.0	-5.0	-4.0	GI50	TGI	LC50
**Leukemia**															
CCRF-CEM	2.383	8.526	9.782	9.663	9.677	0.026	0.068	120	119	119	-99	-97	* 2.07E-6	* 3.51E-6	* 5.96E-6
HL-60(TB)	0.762	3.539	4.221	4.088	4.175	4.201	0.020	125	120	123	124	-97	* 2.16E-5	* 3.63E-5	* 6.10E-5
K-562	0.572	7.827	8.814	8.621	8.211	5.480	6.446	114	111	105	68	81	> 1.00E-4	> 1.00E-4	> 1.00E-4
MOLT-4	1.004	4.344	4.526	4.483	4.615	0.540	0.010	105	104	108	-46	-99	* 2.38E-6	* 5.02E-6	* 1.18E-5
RPMI-8226	6.912	19.179	20.464	20.933	20.784	0.339	0.069	110	114	113	-95	-99	* 2.01E-6	* 3.49E-6	* 6.07E-6
SR	0.173	0.904	1.040	1.102	1.066	0.069	0.003	119	127	123	-60	-98	* 2.50E-6	* 4.69E-6	* 8.81E-6
**Non-Small Cell Lung Cancer**															
A549/ATCC	0.672	5.520	5.451	5.563	3.237	0.512	0.510	99	101	53	-24	-24	* 1.09E-6	* 4.90E-6	> 1.00E-4
EKVX	5.546	11.035	10.602	10.264	10.088	4.125	3.716	92	86	83	-26	-33	* 2.01E-6	* 5.80E-6	> 1.00E-4
HOP-62	1.310	3.447	3.371	3.161	1.201	0.176	0.177	96	87	-8	-87	-86	* 2.43E-7	* 8.17E-7	* 3.41E-6
HOP-92	5.399	13.855	13.405	13.644	13.816	2.397	7.110	95	98	100	-56	20	* 2.09E-6	.	.
NCI-H226	9.434	12.254	12.370	12.351	10.468	1.634	2.729	104	104	36	-83	-71	* 6.31E-7	* 2.02E-6	* 5.32E-6
NCI-H23	3.807	13.460	13.635	13.927	14.102	3.152	2.486	102	105	107	-17	-35	* 2.87E-6	* 7.27E-6	> 1.00E-4
NCI-H322M	3.247	8.307	8.798	8.426	3.172	1.312	0.822	110	102	-2	-60	-75	* 3.16E-7	* 9.50E-7	* 6.80E-6
NCI-H460	1.152	16.510	15.199	16.671	7.977	1.132	0.600	91	101	44	-3	-48	* 7.98E-7	* 8.49E-6	> 1.00E-4
NCI-H522	5.459	11.960	11.271	11.500	12.064	11.715	4.717	89	93	102	96	-14	* 2.64E-5	* 7.52E-5	> 1.00E-4
**Colon Cancer**															
COLO 205	0.651	2.554	2.751	2.794	2.848	1.957	0.772	110	113	116	69	6	* 2.00E-5	> 1.00E-4	> 1.00E-4
HCC-2998	4.637	14.982	12.479	11.703	12.177	5.065	3.128	76	68	73	4	-32	* 2.15E-6	* 1.30E-5	> 1.00E-4
HCT-116	0.718	4.642	4.751	4.644	3.849	0.930	0.543	103	100	80	5	-24	* 2.51E-6	* 1.51E-5	> 1.00E-4
HCT-15	2.224	20.033	17.363	20.972	20.267	9.133	8.699	85	105	101	39	36	* 6.65E-6	> 1.00E-4	> 1.00E-4
HT29	0.858	6.729	6.581	6.677	6.731	2.835	2.443	98	99	100	34	27	* 5.68E-6	> 1.00E-4	> 1.00E-4
KM12	0.515	2.623	2.575	2.899	2.382	0.631	0.329	98	113	89	5	-36	* 2.91E-6	* 1.35E-5	> 1.00E-4
SW-620	0.447	2.511	2.672	2.711	2.612	1.399	0.450	108	110	105	46	0	* 8.59E-6	> 1.00E-4	> 1.00E-4
**CNS Cancer**															
SF-268	1.150	3.512	3.485	3.162	3.329	0.790	0.800	99	85	92	-31	-30	* 2.20E-6	* 5.58E-6	> 1.00E-4
SF-295	2.699	6.825	6.669	6.717	2.539	0.378	0.322	96	97	-6	-86	-88	* 2.87E-7	* 8.76E-7	* 3.55E-6
SF-539	2.612	9.868	9.019	9.207	7.756	0.279	0.114	88	91	71	-89	-96	* 1.35E-6	* 2.77E-6	* 5.68E-6
SNB-19	0.764	1.537	1.609	1.497	1.067	0.317	0.599	109	95	39	-58	-22	* 6.41E-7	* 2.52E-6	.
SNB-75	2.121	4.196	3.919	3.854	3.731	0.248	0.835	87	84	78	-88	-61	* 1.47E-6	* 2.94E-6	* 5.88E-6
U251	0.840	4.125	4.224	4.352	1.869	0.218	0.455	103	107	31	-74	-46	* 5.66E-7	* 1.98E-6	.
**Melanoma**															
LOX IMVI	0.569	3.939	4.255	4.096	4.278	1.393	1.638	109	105	110	24	32	* 5.03E-6	> 1.00E-4	> 1.00E-4
MALME-3M	7.908	14.227	13.979	14.114	14.510	14.466	12.368	96	98	104	104	71	> 1.00E-4	> 1.00E-4	> 1.00E-4
M14	3.018	11.834	12.412	12.550	12.284	1.699	1.272	107	108	105	-44	-58	* 2.35E-6	* 5.08E-6	* 2.78E-5
MDA-MB-435	1.295	4.207	4.297	4.540	4.596	3.028	2.145	103	111	113	60	29	* 2.06E-5	> 1.00E-4	> 1.00E-4
SK-MEL-2	1.837	4.972	5.102	4.516	4.534	1.371	0.860	104	86	86	-25	-53	* 2.10E-6	* 5.91E-6	* 7.68E-5
SK-MEL-28	3.945	7.060	7.074	7.041	7.151	2.328	4.463	100	99	103	-41	17	* 2.33E-6	.	> 1.00E-4
SK-MEL-5	3.148	12.690	11.966	11.144	12.983	3.072	2.809	92	84	103	-7	-11	* 3.04E-6	* 8.69E-6	> 1.00E-4
UACC-257	3.162	6.608	6.559	7.037	6.472	5.119	3.487	98	112	96	57	9	* 1.39E-5	> 1.00E-4	> 1.00E-4
UACC-62	0.869	3.284	3.388	3.478	3.439	1.479	2.257	104	108	107	26	58	.	> 1.00E-4	> 1.00E-4
**Ovarian Cancer**															
IGROV1	1.064	4.205	4.470	4.292	4.211	1.023	1.202	109	103	100	-4	4	* 3.04E-6	.	> 1.00E-4
OVCAR-3	3.350	10.365	9.557	9.686	9.678	3.406	3.402	88	90	90	0	-1	* 2.79E-6	* 1.79E-5	> 1.00E-4
OVCAR-4	4.819	9.106	8.292	8.138	8.642	7.790	4.916	82	77	90	70	2	* 1.98E-5	> 1.00E-4	> 1.00E-4
OVCAR-5	4.584	14.398	14.084	14.455	8.357	1.810	1.793	97	101	38	-60	-61	* 6.51E-7	* 2.45E-6	* 7.83E-6
OVCAR-8	0.817	4.767	4.821	5.039	4.947	0.466	0.346	101	107	105	-43	-58	* 2.34E-6	* 5.11E-6	* 3.00E-5
NCI/ADR-RES	3.596	12.384	12.539	12.874	12.759	2.366	1.570	102	106	104	-34	-56	* 2.47E-6	* 5.66E-6	* 5.18E-5
SK-OV-3	2.453	7.029	6.894	6.569	5.841	1.431	1.906	97	90	74	-42	-22	* 1.61E-6	* 4.36E-6	> 1.00E-4
**Renal Cancer**															
786-0	2.430	5.978	6.262	6.406	5.762	0.301	0.292	109	112	98	-88	-88	* 1.81E-6	* 3.37E-6	* 6.27E-6
A498	3.091	14.250	14.299	14.615	8.513	2.889	2.734	100	103	49	-7	-12	* 9.42E-7	* 7.62E-6	> 1.00E-4
ACHN	1.436	5.807	5.844	5.595	5.725	2.716	1.854	101	95	98	29	10	* 5.00E-6	> 1.00E-4	> 1.00E-4
CAKI-1	0.927	5.749	5.295	5.267	5.537	0.483	1.197	91	90	96	-48	6	* 2.08E-6	.	> 1.00E-4
RXF 393	2.153	.	.	.	.	.	.	.	.	.	.	.	.	.	.
SN12C	1.356	3.860	3.335	3.321	3.505	0.428	0.608	79	78	86	-68	-55	* 1.70E-6	* 3.60E-6	* 7.59E-6
TK-10	2.146	7.289	7.700	7.846	7.347	3.961	3.253	108	111	101	35	22	* 5.97E-6	> 1.00E-4	> 1.00E-4
UO-31	1.458	.	.	.	.	.	.	.	.	.	.	.	.	.	.
**Prostate Cancer**															
PC-3	5.569	.	.	.	.	.	.	.	.	.	.	.	.	.	.
DU-145	1.963	4.648	4.792	4.766	5.341	0.713	1.408	105	104	126	-64	-28	* 2.51E-6	* 4.61E-6	.
**Breast Cancer**															
MCF7	2.853	14.614	12.948	13.536	13.224	7.354	6.161	86	91	88	38	28	* 5.82E-6	> 1.00E-4	> 1.00E-4
MDA-MB-231/ATCC	4.387	15.617	15.111	16.161	15.548	2.461	1.903	96	105	99	-44	-57	* 2.21E-6	* 4.94E-6	* 3.02E-5
HS 578T	1.737	3.359	2.619	2.551	2.198	0.595	0.476	54	50	28	-66	-73	* 8.70E-8	* 2.00E-6	* 6.80E-6
BT-549	8.085	.	.	.	.	.	.	.	.	.	.	.	.	.	.
T-47D	5.448	10.078	9.431	9.609	9.589	10.126	5.905	86	90	90	101	10	* 3.63E-5	> 1.00E-4	> 1.00E-4
MDA-MB-468	4.562	7.434	7.385	7.323	7.185	7.156	3.400	99	96	92	91	-26	* 2.24E-5	* 6.04E-5	> 1.00E-4

Figure S55. NCI's DTP dose-response report for compound **SLT-6**. GI_{50}, TGI and LC_{50} values (expressed as molarity).

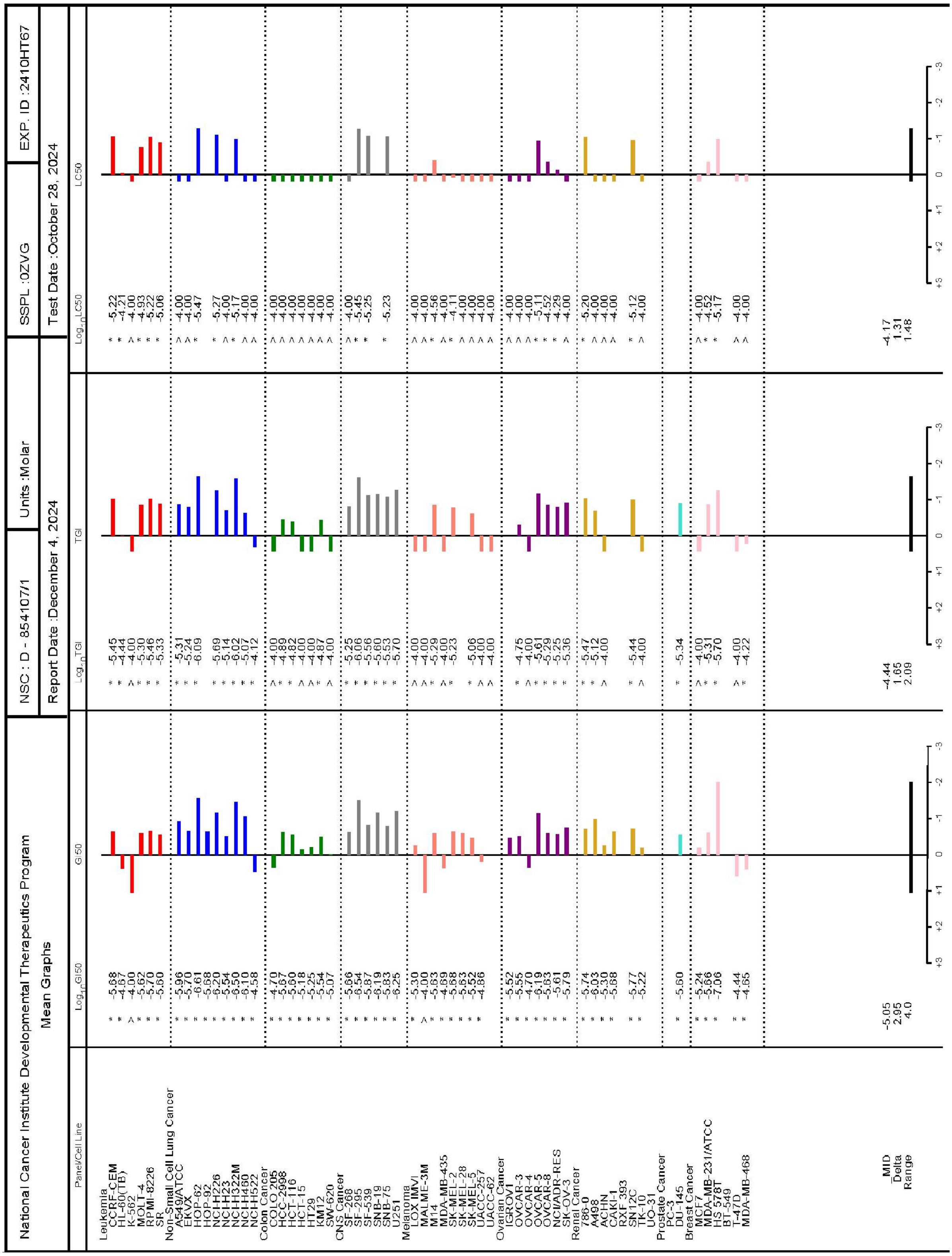

Figure S56. NCI's DTP dose-response report for compound **SLT-6**. Mean graphs of GI_{50}, TGI and LC_{50} values.

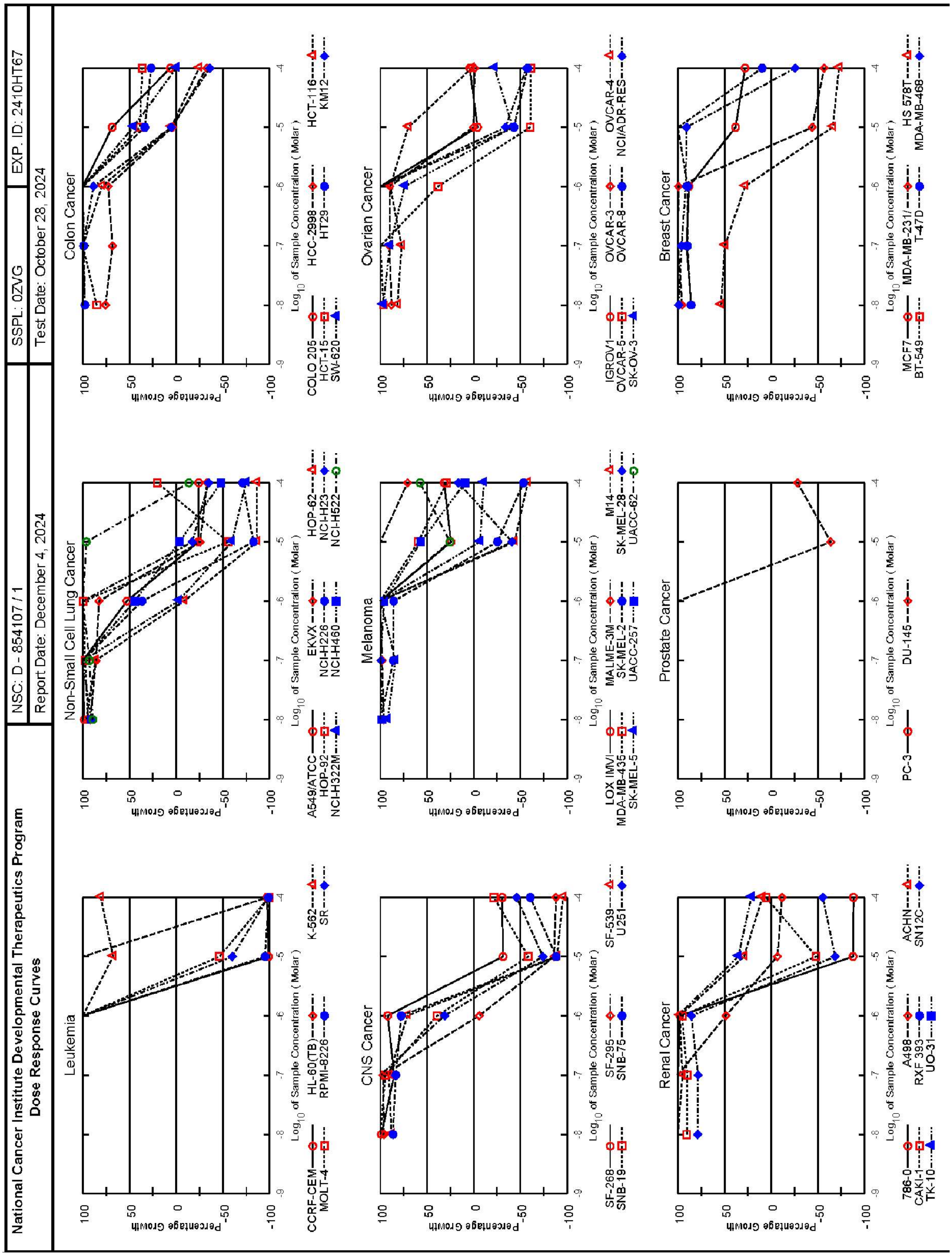

Figure S57. Dose-response curves for **SLT-6.**

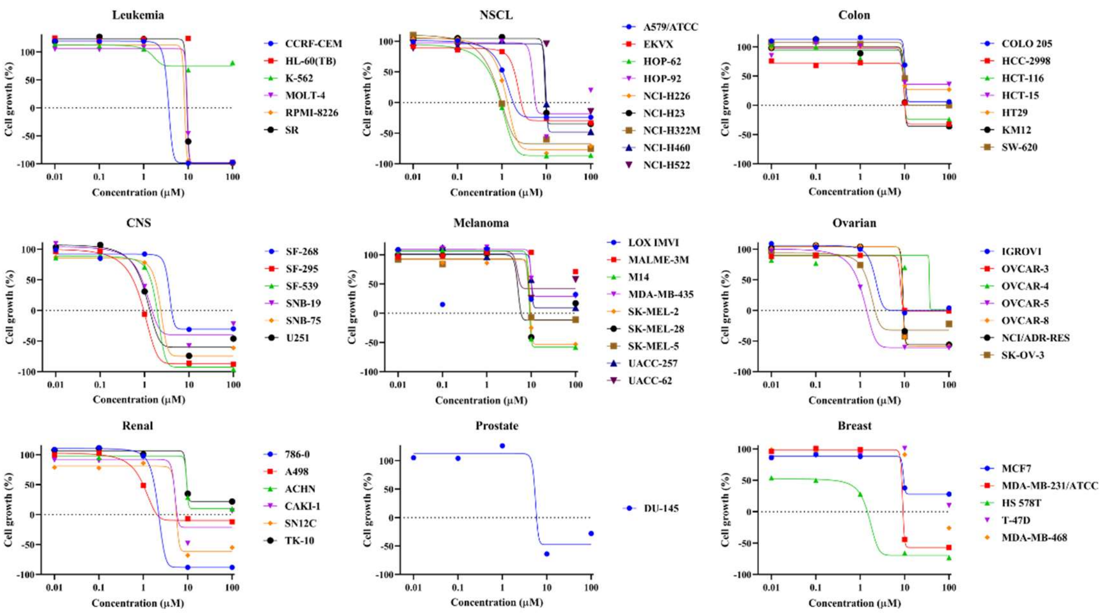

Figure S58. Dose-response curves of **SLT-6** in all the cell lines present in the NCI panel.

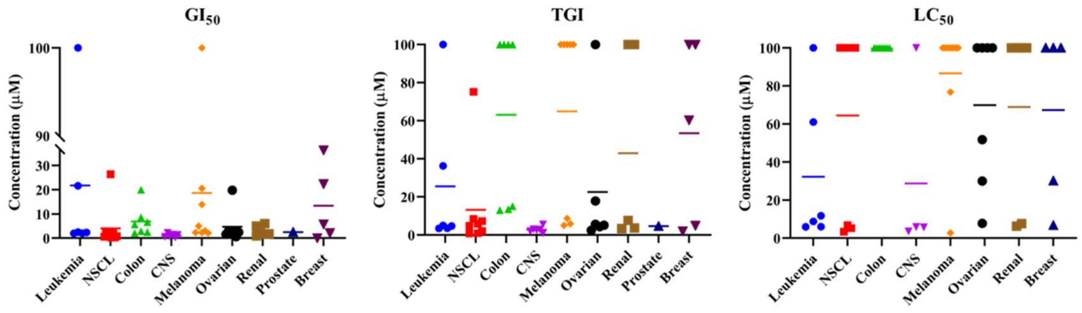

Figure S59. Mean graphs of the GI_{50}, TGI, and LC_{50} values of **SLT-6.**

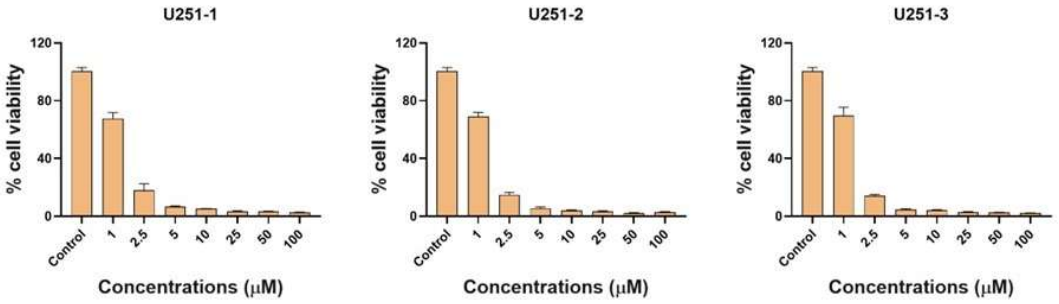

Figure S60. Dose-dependent cytotoxic effect of selenous acid on the U251 cell line after 48 hours of treatment. U251 cells were treated with increasing concentrations of selenous acid (1–100 µM) for 48 hours. Cell viability was assessed using an MTS assay. The cell viability data demonstrated a sharp decline in viability with increasing doses of selenous acid, with near-complete loss of viability observed at concentrations ≥10 µM. The IC_{50} values for selenous acid in U251 cells were calculated as 1.038 µM, indicating consistent potency across the sublines. Data are presented as mean ± SD from at least three independent experiments.

.
